# Correction: Direct and indirect impacts of the COVID-19 pandemic on life expectancy and person-years of life lost with and without disability: A systematic analysis for 18 European countries, 2020–2022

**DOI:** 10.1371/journal.pmed.1004757

**Published:** 2025-09-19

**Authors:** Sara Ahmadi-Abhari, Piotr Bandosz, Martin J. Shipley, Joni V. Lindbohm, Abbas Dehghan, Paul Elliott, Mika Kivimaki

There is an error in the caption of Fig 5. The “per 1,000 population” should not have been indicated. Please see the correct caption of Fig 5 here.

**Fig 5:**
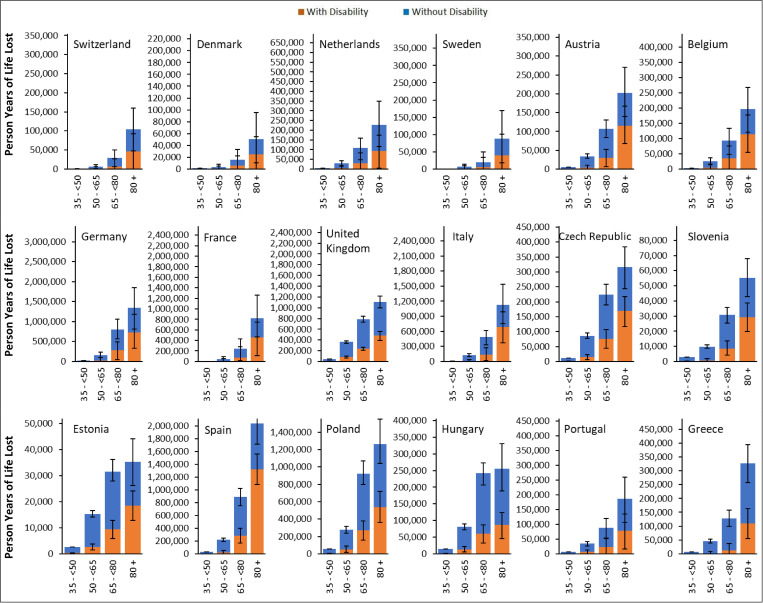
Person-years of life lost between ages 35–100 by age-group and sex, sorted by descending gross domestic product per capita. To enable comparability between countries, the range of the Y-axis is proportional to the total population aged 35+ in each country. Error bars represent 95% Uncertainty Intervals obtained from Monte-Carlo simulation.

Fig 6 was uploaded incorrectly. Please see the correct Fig 6 here.

**Fig 6:**
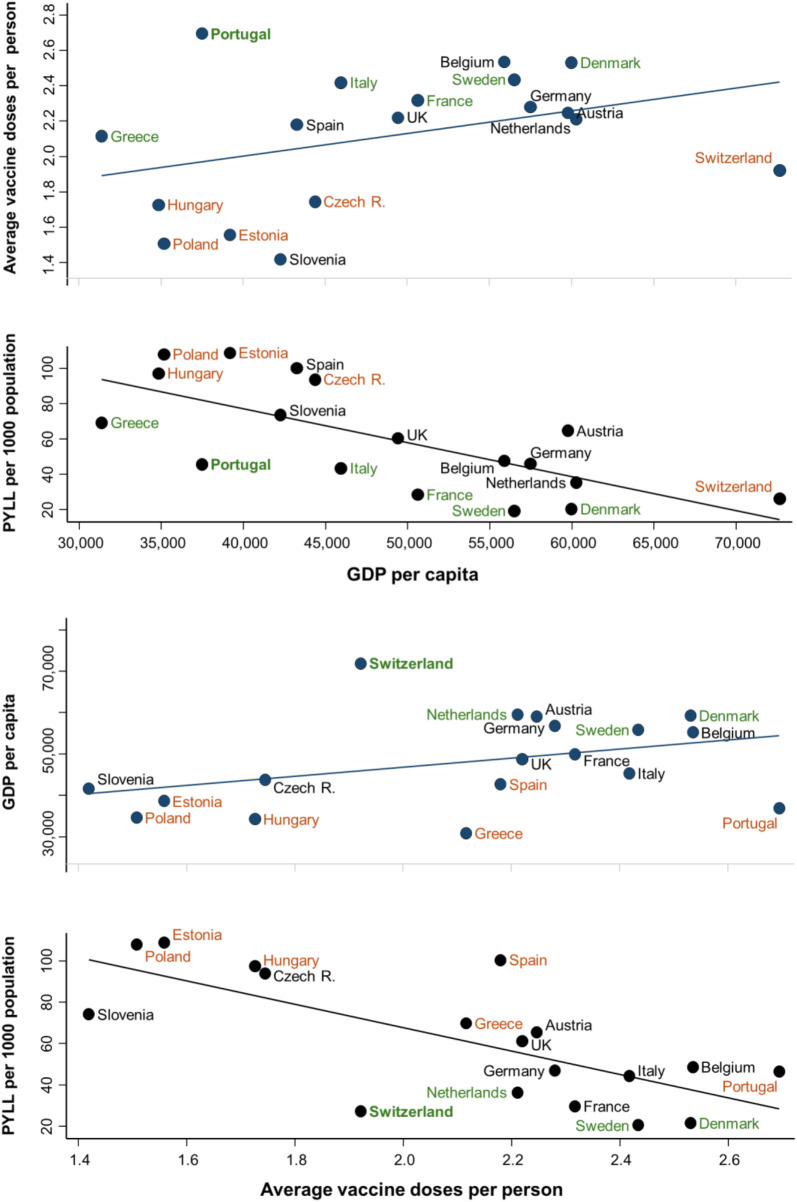
Associations between person-years of life lost per 1,000 population (PYLL) over the years 2020−2022 with Gross Domestic Product (GDP) per capita and COVID-19 vaccination coverage. In the top panel, deviations in countries’ PYLL and vaccination coverage from values expected based on GDP per capita tend to mirror one another. In the bottom panel, deviations in PYLL and GDP per capita from values expected based on vaccination coverage show a similar mirroring. Countries that follow this pattern are labelled in **green** (lower-than-expected PYLL paired with higher-than-expected vaccination coverage [top] or GDP per capita [bottom]), or **orange** (higher-than-expected PYLL paired with lower-than-expected vaccination coverage [top] or GDP per capita [bottom]). Countries not following this pattern are labelled in **black**.
