# Supplementary material for: Direct and indirect impacts of the COVID-19 pandemic on life expectancy and person-years of life lost with and without disability: A systematic analysis for 18 European countries, 2020–2022
Source: PLoS Med. 2025 Mar 11;22(3):e1004541. doi: 10.1371/journal.pmed.1004541 (PMC11896051; doi:10.1371/journal.pmed.1004541)
Supplement: S1 Appendix — Appendix Methods. Table A: Summary of assumptions underlying the Markov models. Table B: Numbers and characteristics of study participants in each country. Table C: Weighted average of age- and sex-specific baseline prevalence estimates for each health state in the model across all countries. Fig A: Weighted average of transition probabilities to cardiovascular disease, cognitive impairment, dementia, and functional impairment by age, across all countries. Fig B: Age- and sex-specific projections for mortality rates by year. Table D: COVID-19 mortality rates according to reported numbers of COVID-19 deaths 2020−2022. Table E: Relative risks of mortality from each state compared to the general population. Fig C: Comparison of mortality rates expected in the absence of the pandemic with estimated actual mortality rates with and without disability over 2020−2022. Table F: Proportion of excess deaths in 2020−2022 that was estimated to occur with disability by age group. Fig D: Age-standardized all-cause and COVID-19 mortality rates in men and women in 2020 and 2021 in the United Kingdom by disease and disability status, comparing the model outputs with those reported by the Office for National Statistics. Fig E: Age-standardized incidence of cardiovascular diseases and Alzheimer’s disease across European countries over time. Appendix Results. Fig F: Person-years of life lost per 1,000 population aged 35 and over, by country, explained and not explained by registered COVID deaths over the years 2020–2022. Table G: Person-years of life lost (PYLL) by cause in the population aged 35 and over during the years 2020–2022. Table H: Disabled and disability-free person-years of life lost (PYLL) in the population aged 35 and over during the years 2020–2022. Fig G: Person-years of life lost with and without disability in the population aged 35 and over, by age-group and sex. Fig H: Person-years of life lost per 1,000 population over the years 2020–2022 by vaccination coverage and [file pmed.1004541.s001.pdf]

S1 Appendix

Title: **Direct and indirect impacts of the COVID-19 pandemic on life-expectancy and person-years of life lost with and without disability: a systematic analysis for 18 European countries, 2020-2022**

Authors: *Sara Ahmadi-Abhari, Piotr Bandosz, Martin Shipley, Joni V Lindbohm, Abbas Dehghan*, Paul Elliott*, Mika Kivimaki**

** Joint Senior authors*

Table of Contents:

Page

Appendix Methods **1**

Survey for Health, Ageing, and Retirement in Europe (SHARE) ………………………………… 1

Assessment of Cardiovascular Disease …………………………………………………….. 1

Assessment of Functional Impairment (disability) ……………………………………… 1

Assessment of Cognitive Function ……………………………………………………….. 2

Case definition of Cognitive Impairment and Dementia ………………………………… 2

Statistical Methods ……………………………………………………………………….. 2

Table A: Summary of assumptions underlying the Markov models…………………….………. 15

Table B: Numbers and characteristics of study participants in each country ……………..…….. 17

Table C: Weighted average of age and sex specific baseline prevalence estimates for each health state in the model across all countries included in the study………………..……………... 18

Figure A: Weighted average of transition Probabilities to cardiovascular disease, cognitive impairment, dementia, and functional impairment by age, across all countries ……………..…... 19

Figure B: Age and sex specific projections for mortality rates by year…………………………... 20

Table D: COVID-19 mortality rates according to reported numbers of COVID-19 deaths 2020-2022. ….………………………………………………………………………….……………. 26

Table E: Relative risks of mortality from each state compared to the general population, a weighted average of all countries modelled in the study…………………………………..……... 27

Figure C: Comparison of mortality rates expected in the absence of the pandemic with estimated actual mortality rates with and without disability over 2020-2022 ………………….. 30

Table F: Proportion of excess deaths in 2020-2022 that was estimated to occur with disability by age-group …………………………………………………………………...…………… 31

Figure D: Age-standardized all-cause and COVID-19 mortality rates in men and women in 2020 and 2021 in the United Kingdom by disease and disability status, comparing the model outputs with those reported by the Office for National Statistics……………...…………………. 32

Figure E: Age-standardised incidence of cardiovascular diseases and Alzheimer’s disease across European countries over time ……….………………………...………………………… 33

Appendix Results **34**

Figure F: Person-years of life lost per 1000 population aged 35 and over, by country, explained and not explained by registered COVID deaths over the years 2020-2022. ……………………...… 35

Table G: Person-years of life lost (PYLL) in the population aged 35 and over during the years 2020-2022, estimated by comparing Markov models with projected and observed all-cause mortality rates in thousands (000) …..………………………………………………………... 37

Table H: Disabled and disability-free person-years of life lost (PYLL) in the population aged 35 and over during the years 2020-2022, estimated by comparing Markov models with projected and observed all-cause mortality rates, in thousands (000) ………………………………..…. 39

Figure G: Person-years of life lost in the population aged 35 and over, by age-group and sex, estimated by comparing Markov models with projected and observed all-cause mortality rates .. 41

Figure H: Person-years of life lost per 1000 population over the years 2020-2022 by vaccination coverage and 2019 Gross Domestic Product per capita ……………………………………….. 43

Figure I: Change in Life-Expectancy at 35 over time in the decade leading to the pandemic (2009-2019) ……………………………………………………………………….…………… 44

Table I: Life-expectancy at age 35 (LE) with and without disability by country and year. ……... 46

Figure J: Life-expectancy at age 35 by gross domestic product per capita, 2019-2022 ………… 53

References **54**

Appendix Methods

Survey for Health, Ageing, and Retirement in Europe (SHARE) and the English Longitudinal Study of Ageing (ELSA)

SHARE study participants were randomly drawn from those aged 50+ in population registers and their cohabiting partners [1-7], which was the year 2004-2005 (Wave 1) [1, 2] for Austria, Belgium, Denmark, France, Germany, Greece, Italy, The Netherlands, Spain, Sweden, and Switzerland; 2006-2007 (Wave 2) [1, 3] for Czech Republic, and Poland; and 2010-2011 (Wave 4) [1, 4] for Estonia, Hungary, Portugal, and Slovenia. With the exception of SHARE wave 3 (2008-2009, not included in analysis), at each biannual data collection wave extensive demographic, medical and lifestyle data were collected, with the latest data collection wave (wave 7) [7] used in the present analysis completed in 2017. The English Longitudinal Study of Ageing follows similar and comparable methods with SHARE. We used data collected at the first 8 biennial data collection waves of ELSA (2002-2017) [8] to calculate UK model inputs.

For each study in SHARE and ELSA, refreshment samples are recruited at biannual waves after baseline to maintain representativeness of the study population. With application of survey weights, therefore, estimates of disease incidence and prevalence from these studies represent a weighted average across strata of disease severity, associated comorbidities, and socioeconomic status of the population (Table A). Appropriate ethical approvals were obtained for each of the SHARE studies and written informed consent was obtained from all participants [1-7]. Numbers and baseline characteristics of participants recruited in the study in each country are presented in Table B.

Assessment of Cardiovascular Disease

Cardiovascular disease in SHARE and ELSA was ascertained by self-reported doctor diagnosis of myocardial infarction, stroke, angina, coronary artery bypass grafting (CABG), or death from cardiovascular causes [1-8]. Incidence of cardiovascular disease was defined as first ever record of disease or attributable intervention for each participant.

Assessment of Cognitive Function, cognitive impairment, and dementia

Cognitive function assessments administered at SHARE and ELSA include i) orientation to time, day, month, year; ii) immediate and delayed memory: a list of ten nouns, one every two seconds, were presented and the participant was asked to recall as many words as possible immediately and after a short delay; iii) verbal fluency: participants were asked to name as many animals as they could in one

minute; iv) numeracy function: participants were asked to solve four simple mathematics problems [1-8]. Orientation to time was used to assess concentration, scores on the immediate and delayed recall were used as a measure of memory function, and scores on the animal naming and numeracy test were used to measure verbal fluency and executive function.

An operational case definition for cognitive impairment was adapted from the criteria for cognitive impairment no dementia (CIND) [9, 10]. Cognitive impairment was defined as impairment in 2 or more domains of cognitive function. Impairment in each domain of cognitive function is defined as a score of 1.5 standard deviations (SD) below the mean or lower compared to the population aged 50-80 with same level of education in each country [11]. Education level was categorized in three levels: no qualification; high school level; and higher (university) education. We did not find considerable differences between men and women in the distribution of cognitive function scores after adjustment for age and education. We did not find evidence of large learning effects in consecutive cognitive function tests. The annual age- and sex-specific decline observed among participants who conducted the tests 4 or more years apart was similar to the decline observed in the participants who conducted the tests two years apart. The cognitive assessment was considered invalid if the participant had responded to less than three tests on the cognitive battery. To avoid the effect of transient cognitive decline, resulting from delirium or other mental disorders, if the participant improved by 1-SD or more on cognitive tests at the consecutive waves, they were considered to not have had cognitive impairment. A self-reported doctor diagnosis of cognitive impairment was also considered as cognitive impairment for the purpose of this study [11].

Dementia was defined as co-existence of cognitive and functional impairment (as defined below), or a participant or proxy informant's self-report of a doctor diagnosis of dementia [11].

Assessment of Functional Impairment (disability)

SHARE and ELSA participants or proxy informants were asked about the ability of the participant to independently conduct basic activities of daily living (ADL) [1-8]. The ADLs are key tasks related to self-care and consist of getting in or out of bed, walking across a room, bathing or showering, using the toilet, dressing, cutting food and eating. Impairment in independently performing one or more activities of daily living lasting for more than three months was defined as functional impairment. Impairment in conducting ADLs reported once, where the participant fully recovered at all further waves of data collection, was considered transient and not categorised as functional impairment [11].

Markov Models: Statistical Methods

Transitions of the population aged 35+ through health states to death were modelled using a Markov method. Initially populated using age-sex-specific population numbers and prevalence estimates, calendar-time specific transition probabilities between states of the Markov model are applied at each 1-year iteration to predict numbers of deaths and prevalence of each of the health state at subsequent calendar years.

Below we provide the Markov model calculations. The statistical methods applied to derive model inputs are described in the signposted subsections.

Note 1: *All Markov models are run separately for men and women in each country. All parameters are age, sex, and country specific*

Note 2: *Elements of Matrices and vectors are displayed in italics*

Formula	Description
$\text{Prevalences}_{a,0} = [V_{1,a,0}, V_{2,a,0}, \ldots, V_{10,a,0}]$<br><br>$V_{s,a,0} \sim N(\mu_{s,a,0}, (\sigma_{s,a,0})^2)$<br><br>$V_{s,a,0}$: prevalence of State s for age a at time 0	Prevalences $_{a,0}$: Vector containing sex-specific prevalence of each state (S_i) for age a at the baseline time=0<br><br>S_1: Disease-free state<br>S_2: Cardiovascular disease, no disability<br>S_3: Cardiovascular disease and cognitive impairment, no disability<br>S_4: Cognitive impairment, no disability<br>S_5: Cardiovascular disease (CVD) and disability<br>S_6: CVD and cognitive impairment with disability (CVD+dementia+disability)<br>S_7: Cognitive impairment with disability (dementia+disability)<br>S_8: Disability, no cardiovascular disease or cognitive impairment<br>S_9 : COVID deaths ($V_{9,a,0}$=0 at baseline)<br>S_{10}: Non-COVID deaths ($V_{10,a,0}$=0 at baseline)<br><br>* Input data to the Markov models: Methods described in subsection SM1 below

$M_{a,0}$	$M_{a,0}$: Vector containing sex-specific initial population for age a at time=0<br><br>* Input data to the Markov models, obtained from official population statistics
For t=0<br><br>$N_{a,0} = M_{a,0} * \text{Prevalences}_{a,0}$	$N_{a,0}$: Vector containing sex-specific numbers of people in each state for age a at baseline (time=0)<br><br>* Markov model calculations: Details in subsection SM1
For persons reaching age 35 and entering the model at time t:<br><br>$N_{35,t} = [N_{1,35,t}, 0, 0, 0, 0, 0, 0, 0, 0, 0]$	$N_{35,t}$: Numbers of persons who reach age 35 at time t are calculated from life-tables using data for observed population numbers and mortality rates for persons aged 0-35. Persons aged 35 who enter the model are assumed to be free of disease and disability, in other words enter the model from state 1.<br><br>* Input data: Methods described in the last paragraph of subsection SM1
$T_{a,0}=\begin{bmatrix} P1,1,a,0 & \cdots & P1,10,a,0 \\ \vdots & \ddots & \vdots \\ P10,1,a,0 & \cdots & P10,10,a,0 \end{bmatrix}$<br><br>$P_{i,j,a,0} \sim N(\omega_{i,j,a,0}, (\delta_{i,j,a,0})^2)$<br><br>$P_{i,j,a,0}$ is the probability of transition from state i to state j for age a at time 0	$T_{a,0}$: Matrix of sex-specific transition probabilities between each pair of states for persons aged a at baseline (time=0)<br><br>* Input data: Methods described in subsection SM2 for transition probabilities between States 1-8 and in subsection SM3 for transition probabilities to mortality

$C=\begin{bmatrix} C1,1 & \cdots & C1,10 \\ \vdots & \ddots & \vdots \\ C10,1 & \cdots & C10,10 \end{bmatrix}$<br><br>$C_{i,j} \sim N(\theta_{i,j}, (\nu_{i,j})^2)$<br><br>$C_{i,j}$ is the calendar effect for the probability of transition from state i to state j	C: Matrix of calendar effects for transition probabilities between each pair of states<br><br>The calendar effect is the factor by which a transition probability increases/decreases from one year to the next<br><br>* Input data: Methods described in subsection SM3 for trends in mortality rates. Methods applied to derive calendar effects for transition probabilities between States 1-8 are described in subsection SM4.
$T_{a,t} = T_{a,t-1} * C$<br><br>Note: Since $P_{i,j,a,t} \sim N(\omega_{i,j,a,t}, (\delta_{i,j,a,t})^2)$ and $C_{i,j} \sim N(\theta_{i,j}, (\nu_{i,j})^2)$<br><br>The standard error for $P_{i,j,a,t}$ is approximately $\sqrt{(\delta_{i,j,a-1,t-1})^2*(\nu_{i,j})^2+(\delta_{i,j,a-1,t-1})^2*(C_{i,j})^2+(P_{i,j,a-1,t-1})^2*(\nu_{i,j})^2}$	$T_{a,t}$: Matrix of transition probabilities at age a for time t<br><br>To simplify, the standard errors (SE) of the transition probabilities ($P_{i,j,a,t}$) at each subsequent year increase proportionately to the magnitude and SE of the calendar effect plus the magnitude and standard error of the transition probability. This roughly translates to an increase in SE by a factor of $\sqrt{years\ since\ baseline + \varepsilon}$.<br><br>* Markov model calculations: Details are provided in subsection SM4.<br>Details on the factors affecting standard errors and the impact on uncertainty intervals are described in subsection SM6
$N_{a,t} = N_{a-1,t-1} * T_{a-1,t-1}$	$N_{,a,t}$: Vector of the number of people aged a in each state at time t is the product of the vector of the number of people, and the matrix of transition probabilities, at age a-1 and time t-1<br><br>* Markov model calculations

$L_{a,t} = [N_{1,a,t}, N_{2,a,t}, \ldots, N_{8,a,0}, 0, 0]$	$L_{a,t}$: Number of people aged a who are alive at time t. Note states 9 and 10 represent the death states in the model.<br><br>* Markov model calculations
$PYL_{a,t} = L_{a+1,t+1} + L_{a+2,t+2} + \ldots + L_{100,t+100-a} + (L_{a,t} - L_{100,t+100-a})/2$	$PYL_{a,t}$: Vector of person-years of life that persons aged a at time t expect to live in each state until death or age 100. Note, persons who died from year t to year t+1 contribute half a year to PYL<br><br>* Markov model calculations

Note: $PYL_{a,t} = [PYL_{1,a,t}, PYL_{2,a,t}, \ldots, PYL_{8,a,t}, 0, 0]$

$$\text{Total PYL } (TPYL_{a,t}) = PYL_{1,a,t} + PYL_{2,a,t} + PYL_{3,a,t} + PYL_{4,a,t} + PYL_{5,a,t} + PYL_{6,a,t} + PYL_{7,a,t} + PYL_{8,a,t}$$

$$\text{Disability PYL } (DPYL_{a,t}) = PYL_{5,a,t} + PYL_{6,a,t} + PYL_{7,a,t} + PYL_{8,a,t}$$

$$\text{Disability-Free PYL } (DFPYL_{a,t}) = PYL_{1,a,t} + PYL_{2,a,t} + PYL_{3,a,t} + PYL_{4,a,t}$$

* Markov model calculations

For each country, 3 Markov models were run as described below:

Model 1: Person-years of life that would have been expected to be lived in the absence of the pandemic. Model inputs include transition probabilities, and mortality rates expected following pre-pandemic trends.

Model 2: Expected mortality rates from Model 1 were adjusted for the additional registered deaths due to COVID-19 by age and sex for the years 2020-2022

Model 3: Mortality rates from each state derived from observed all-cause mortality rates by country, age group, and sex for the years 2020 to 2022 were applied.

To calculate person-years of life lost (PYLL) by each state for persons aged a who died at time t:

Total PYLL $_{a,t}$ (TPYLL $_{a,t}$) = Model 1_ TPYL $_{a,t}$ – Model 3_ TPYL $_{a,t}$

PYLL $_{a,t}$ attributable to registered COVID deaths (COVID_ PYLL $_{a,t}$) = Model 1_ TPYL $_{a,t}$ – Model 2_ TPYL $_{a,t}$

PYLL $_{a,t}$ attributable to non-COVID deaths (nonCOVID_ PYLL $_{a,t}$) = Model 2_ TPYL $_{a,t}$ – Model 3_ TPYL $_{a,t}$

PYLL due to disability $_{a,t}$ (DPYLL $_{a,t}$)= Model 1_ DPYL $_{a,t}$ – Model 3_ DPYL $_{a,t}$

Disability-Free PYLL $_{a,t}$ (DFPYLL $_{a,t}$)= Model 1_ DFPYL $_{a,t}$ – Model 3_ DFPYL $_{a,t}$

PYLL with and without disability were calculated as below:

Total PYLL for men (or women) aged i to j who died at time t = $\sum_{a=i}^{j} TPYLL_{a,t}$

PYLL due to disability = $\sum_{a=i}^{j} DPYLL_{a,t}$

PYLL without disability = $\sum_{a=i}^{j} DFPYLL_{a,t}$

PYLL attributable to COVID-19 deaths = $\sum_{a=i}^{j} COVID\_PYLL_{a,t}$

PYLL attributable to nonCOVID deaths = $\sum_{a=i}^{j} nonCOVID\_PYLL_{a,t}$

Total PYLL for persons (men and women) of all ages who died at time t = ($\sum_{a=35}^{100} TPYLL_{a,t}$ for men) + ($\sum_{a=35}^{100} TPYLL_{a,t}$ for women)

* Markov model calculations: Details are provided in subsection SM5 and the Methods section in the manuscript.

Probabilistic sensitivity analysis: Monte Carlo simulation with 500 iterations:

In each iteration, each parameter is chosen at random from the relevant distribution for prevalences [$V_{s,a,0} \sim N(\mu_{s,a,0}, (\sigma_{s,a,0})^2)$], transition probabilities [$P_{i,j,a,t} \sim N(\omega_{i,j,a,0}, (\delta_{i,j,a,0})^2)$], and calendar effects [$C_{i,j} \sim N(\theta_{i,j}, (\nu_{i,j})^2)$]

The 2.5 and 97.5 percentile of values generated from 500 iterations of the model provide the 95% Uncertainty Intervals.

* Monte Carlo simulation: Explained in detail in subsection SM6 and in the Methods section in the Manuscript under subtitle “Probabilistic sensitivity analysis”

The assumptions underlying the Markov models are presented in Table A.

SM1: Country-specific prevalence of each state by age and sex

The baseline age and sex specific prevalence of each health state of the model was calculated using country specific waves of ELSA or SHARE. The prevalence of each state by age and sex is the proportion of persons by age and sex that fulfil the definition for each state (S1 – S8) as described above. Data from all waves of ELSA or SHARE for each country were pooled together and attributed to mid-point of the data collection timeframe. To evaluate the validity of this method, obtained values were compared with, and corresponded to, values observed at the data collection wave closest in time to the mid-point of the data collection timeframe. For illustration purposes only, the weighted average of the prevalence estimates across all countries included in the study is presented in Table C. Prevalence estimates entered in the models were all country specific.

Life tables were constructed to calculate numbers of people who reach age 35 at each calendar year and enter the model. The entering cohort of people aged 35 is assumed to be free of cardiovascular disease, cognitive and functional impairment.

SM2: Country-specific transition probabilities between states representing CVD, cognitive impairment, dementia and disability by age and sex

The baseline age-sex-specific probability of transition from $state_i$ to $state_j$ in the model (hereafter referred to as transition probability (P_{ij})) for the mid-point of the data collection timeframe of each SHARE (/ELSA) study were obtained by fitting a Cox proportional hazards regression model with $state_j$ as outcome and terms for age, sex, interaction of age and sex, country, interaction terms for country with age and sex, and a variable defining the initial state ($state_i$). Transition probabilities to CIND (states 3, and 4 in figure 1) additionally included terms for age-squared and its interaction with sex. Transitions from wave n to wave n+1 in ELSA or SHARE were pooled together so that each individual contributed as many observations as corresponded to the number of 2-, 4- or 6-year epochs in which they participated in the study until being censored. Transition probabilities over a two year period were obtained from the formula $P=1-(S_0(2)^{exp(LP)})$ where "$S_0(2)$" denotes the baseline survival function at two years, "exp" denotes the exponential function, and "LP" is the linear predictor obtained from the Cox-regression model for each country and each year of age, sex, and baseline health state. The two-year probability (P) was then translated into one-year transition probability using the formulae TP=1-exp((ln(1-P))/2) (Figure A).

Since all prevalence estimates and transition probabilities calculated from pooling epochs of data collection waves were attributed to the mid-point of the data collection time-frame, these time points were the baseline for the iterative Markov model.

SM3: Country-specific transition probabilities to mortality by age and sex

Projections for expected mortality rates

To obtain expected all-cause mortality rates in the absence of the pandemic in each country, observed mortality rates over 1998-2019 were projected to 2080 by sex and five-year age bands. To project mortality rates, P-splines smoothed lines were fitted to the natural logarithm (ln) of mortality rates from 1998 to 2019 by sex- and five-year age groups. P-splines smoothed ln-mortality rates declined over two decades (1998-2019) and the magnitude of decline in the fitted P-splines smoothed curves also consistently declined. To estimate the magnitude of change in mortality rates by age and sex, linear regression models with change in ln-mortality rates in consecutive years as the outcome, and age, sex, calendar year, and interactions of the terms as the predictors were fitted. The magnitude of decline in mortality rates by age and sex were projected to 2080 (Figure B). Mortality rates were projected to 2080 to ensure PYLL for each person in the population aged 35 and older in 2020 is calculated to be able to reach age 95 or older in 2080. Projected mortality rates from 2006 to 2016, derived from data spanning 1995-2005, closely matched observed mortality rates, as previously shown [11-13], demonstrating the reliability and validity of our methods. Projections for mortality rates are displayed in Figure B. Mortality rates for the years 2020-2022 were adjusted according to the additional registered deaths due to COVID-19 (Table D).

COVID-19 mortality rates by age and sex

Age, and sex specific COVID-19 mortality rates were available for the UK from the UK Office for National Statistics, and for other countries from the demography of COVID-19 deaths database [14]. This database managed by the French Institute for Demographic Studies (INED) obtained and documented COVID-19 deaths by age and sex from each country's national authorities and official statistics bureaus between 2020 and April 2022. Overall COVID-19 mortality rates were obtained for each country, separately for 2020, 2021, and 2022 by dividing numbers of COVID-19 deaths to mid-year population numbers. In countries where age-sex-specific COVID-19 death rates were documented to April 2022, the age-sex-specific to overall mortality rate ratios observed between January to April 2022 were applied to overall COVID-19 death rates in 2022 in each country to obtain age-sex-specific COVID-19 mortality rates for 2022. COVID-19 death rates by age and sex were not available in publicly accessible databases for Czech Republic, Estonia, Greece, Hungary, Poland, and Slovenia by

the time this analysis was conducted. Among countries with available data, the age and sex specific to overall COVID-19 mortality rates were similar between countries. To calculate age and sex specific COVID-19 mortality rates in countries where this data were not available, we assumed the ratio of the age-sex-specific COVID-19 mortality rates to overall COVID-19 mortality rates in each country is similar to the corresponding rate ratio observed in the UK where granular data was available to end 2022. We compared the numbers of deaths calculated from this method with observed numbers of COVID-19 deaths to ensure internal validity of the calculated age-sex-specific COVID-19 mortality rates.

Observed mortality rates

Observed all-cause mortality rates by country, age group (0-14, 15-64, 65-74, 85+), and sex for the years 2020 to 2022 were obtained from STMF [15]. Mortality rates for 5-year age groups incorporated in the model were obtained using the "ungroup" R package which applies the penalized composite link method ungroup age-specific distributions from the coarsely grouped data [16].

Mortality rates by disease and disability status

To estimate age, sex, and calendar-year specific mortality rates from each health-state in the model, we assumed that the hazard of death by health, disease, and disability status remain proportional to the baseline hazard of death over time by age and sex, as observed during the data collection period. We used the observed all-cause mortality by age and sex as baseline hazard. For each country, the hazard ratios of death from each health state in the Markov model to overall mortality rates by age and sex were calculated using country-specific SHARE and ELSA data. Health state specific transition probabilities to mortality were calculated by applying the hazard ratios to overall projected and observed mortality rates to obtain health state specific mortality rates for each country.

For illustration purposes, the weighted average of the health state specific to all-cause mortality hazard ratios across all countries included in the study is presented in Table E.

Assessment of the reliability of the method for estimation of mortality rates by disability status

Increased all-cause mortality due to the pandemic or other causes, are likely to disproportionately affect older age groups, or persons with disease or disability. In this study we used the hazard ratio of mortality with disability to all-cause mortality by age and sex to estimate mortality rates with disability over 2020-2022. The hazard ratios are derived from data collected prior to the pandemic. We assumed the

relative risk, or the hazard of mortality with disability to the hazard of all-cause mortality remains proportional over time. This assumption translates to increases in mortality rates with disability being a multiplication of the hazard ratio larger than increases observed in all-cause mortality. The method therefore is likely to account for older persons and those with disability being disproportionately affected over the pandemic.

To test this assumption and for illustration purposes (Figure C), we present the estimated and expected mortality rates with and without disability for men and women aged 60 and 80 in Poland, the United Kingdom, and Switzerland where excess mortality during 2020-2022 was higher, average levels, or lower among the 18 studied countries. Our estimates of the increase in mortality rates from 2017 to the 2020-2022 pandemic years were substantially higher for mortality with disability than mortality without disability, and in Poland compared to the United Kingdom and Switzerland. The increase in mortality rates with disability were larger for persons aged 80 than the younger age-group, and larger for men than for women.

We defined excess mortality as the difference between observed all-cause mortality rates than those expected if the pandemic had not happened, and pre-pandemic trends had continued. We examined the proportion of excess deaths that would be associated with disability in our calculations. In all 18 countries combined, we estimated 65% of excess deaths in the 80+ age group to be with disability. Disability accounted for 44 % of excess mortality in the 65-79 age group, 24% of excess mortality in the 50-64 age group, and 11% in 35-49 age group. The proportion of excess deaths by disability status by age-group and country are presented in Table F.

To evaluate the reliability of this assumption with independent data, we compared model outputs with observed all-cause and COVID-19 mortality rates by disability status over the pandemic years in the UK where such data were available. A report from the Office for National Statistics provides age-standardized all-cause and COVID-19 mortality rates by disability status between January 2020 and March 2022 [17]. Disability status was defined using the self-reported responses to the 2011 Census question; "Are your day-to-day activities limited because of a health problem or disability which has lasted, or is expected to last, at least 12 months? - Include problems related to old age" (Yes, limited a lot; Yes, limited a little; and No). We compared these estimates with age-standardized mortality rates by disease and disability status obtained from our model for the UK. Given the differences in definitions and ascertainment, we did not expect the estimates for mortality to be an exact match. Comparison of the estimates indicate our mortality estimates by disability status over the pandemic years are unlikely to be underestimated (Figure C).

SM4: Calendar effects for incidence of cardiovascular disease, dementia, and disability

To account for cohort effects, and to obtain calendar year specific transition probabilities at each age by sex and country, the effect of calendar time was imposed on the transition probabilities for each year since baseline to obtain transition probabilities for that age at the next calendar year.

The calendar decline in age-specific cardiovascular incidence, mortality, and incidence of dementia observed in ELSA and SHARE data was parallel across the 18 countries modelled in the present study. The parallel calendar declines were similarly observed in data obtained from Institute of Health Metrics and Evaluation [18] (Figure E). Cox proportional hazard models with age, sex, and calendar year with interaction terms with country were fitted to obtain the calendar effect for the transition probabilities to each of the health states. Calendar effects were assumed to become smaller in magnitude over time, similar to the calendar effects observed for the decline in mortality rates.

SM5: Calculation of Person-Years of Life Lost by disability and cause of deaths (COVID/Non COVID)

For each country, we developed and compared three Markov models as explained in the methods section of the manuscript and in the table of calculation formula presented above. The difference in Person-Years of Life for each age at time t provide an estimate of the loss in person-years of life with and without disability attributable to the pandemic, defined as the years of life by disability status that would have been expected to be lived in the absence of the pandemic (Model 1), that were "lost" due to deaths that would not have been expected to occur at that time in 2020-2022 if the pandemic had not happened (Models 2 and 3).

SM6: Probabilistic Sensitivity Analysis (Monte Carlo simulation)

To derive uncertainty intervals for all outputs we ran Monte Carlo simulations with 500 iterations. To account for the uncertainty in model inputs, each input parameter of the Markov models, including baseline prevalence estimates, transition probabilities, and the calendar effects for transition probabilities by country, sex, single year of age, and where applicable, calendar year, is entered as a normal distribution with the standard errors derived from the methods described above. In each iteration of the Monte Carlo simulation, each input parameter is randomly selected from these normal distributions. The 2.5 and 97.5 percentile of the distribution of outputs obtained from 500 iterations constitute the 95% uncertainty intervals. For further details, please see mathematical calculations presented in the Table above under the "Statistical Methods" section.

The width of the 95% uncertainty intervals depend on the standard error of the input values. The standard errors of the transition probabilities increases with calendar years since baseline, due to multiplication of transition probabilities by calendar effects in each year (mathematical calculations presented above). This roughly translates to standard errors for transition probabilities increasing by a factor of the square root of years since baseline. All input data and the standard errors for the input parameters were derived from country specific data. The standard errors for input estimates were larger in countries where data is derived from smaller sample sizes. Also, in countries or age-groups where there is larger year-on-year fluctuations in the incidence of disease, disability, or mortality rates (i.e., there is larger deviation in observations from the line of best fit) the standard errors from the transition probabilities obtained from the Cox-regression models were larger, leading to wider uncertainty intervals.

Table A: Summary of assumptions underlying the Markov models.

Assumption	Justification
The Markov models track the health transitions of the population aged 35 and over through health states to death. The input data for the probabilistic Markov model are the population size in each age and sex stratum, initial health state prevalence values, and transition probabilities by age, sex, and calendar year.	
**Population numbers by age and sex**	
Estimates for population numbers by sex and 5-year age-groups at model baseline were obtained from the EU Official statistics. At each 1-calendar-year iteration of the model, men and women reaching age 35 were entered. The predictions for number of people aged 35 by year were obtained from life-tables.<br>Assumption 1: predictions for numbers of people reaching age 35 are realistic;<br>Assumption 2: migration is not a major source of bias.	
**Starting prevalence values**	
Initial prevalence of health states in the model by age and sex were obtained from the Survey for Health Ageing and Retirement in Europe (SHARE) and the English Longitudinal Study of Ageing (ELSA)<br>Assumption 3: Each SHARE (/ELSA) study is a representative of the population of the respective country.	Accuracy of prevalence values depends on how well SHARE and ELSA represent the target population. SHARE and ELSA study participants aged 50 and over were selected at random. The core participant's cohabiting partners, including adults aged below 50, were also enrolled in the study. To ensure study participants form a representative sample, survey weights are applied. To maintain representativeness at every phase of data collection, refreshment samples are recruited to the study periodically. Comparisons of the socio-demographic characteristics of participants against results from the national census indicated that the SHARE and ELSA samples were broadly representative of the population.
To improve statistical power, all waves of SHARE (/ELSA) data for each country were pooled. Prevalence estimates of CVD, cognitive, and functional impairment that define the health states were obtained from pooled data and attributed to the mid-point of the data collection time-frame as the baseline of the model.<br>Assumption 4: Prevalence estimates from pooled waves of data provide a precise and accurate estimate of prevalence at mid-point of the data collection time-frame.	The prevalence values obtained from the pooled SHARE and ELSA data matched the prevalence values obtained at the mid-point.<br>The model is run for several iterations with the baseline input data to ensure starting prevalence estimates are consistent with the TPs and mortality rates.
Assumption 5: The prevalence of each health state at each calendar year from the starting point onwards, equals number of persons who were in that health state in the previous year, plus new incident cases, minus those who made the transition to another health state or died from any cause. Number of new incident cases and numbers of death were determined by transition probabilities to and from that condition.	Epidemiologic concept applied to Markov models.
**Transition probabilities**	
Transition probabilities were obtained as a function of age and sex from incident cases between wave n and n+1 in SHARE and ELSA. As with estimates of prevalence values, the transition probabilities obtained from	Incidence of cardiovascular disease and dementia by age and sex were consistent with age, and sex specific incidence values obtained from independent external sources for the corresponding calendar year. Deaths predicted by the Markov model using input data of earlier years matched observed mortality rates

pooling SHARE and ELSA epochs were attributed to the mid-point of the data collection period.<br>Assumption 6: Transition probabilities, (equivalent to incidence by age/sex/calendar year) for cardiovascular disease, dementia, functional impairment, and mortality in SHARE and ELSA are similar to those for the target population.	
Assumption 7: Transition probabilities are equivalent to a weighted average across the spectrum of the severity of each condition thus varying severities among people in each health state is accounted for.<br>Similarly, survival of persons with each condition is assumed to be equivalent to the weighted average of survival of persons with different levels of severity.	Modelling is based on a single transition probability for each age/sex/calendar-year strata and health transition. The probability of death or development of functional impairment among those with cardiovascular disease or cognitive impairment is dependent on the severity of cardiovascular disease or cognitive impairment. Under the assumption that SHARE and ELSA participants are a representative sample of the population (see above), the spectrum of the severity of conditions (e.g. cardiovascular disease, or cognitive impairment) observed in SHARE and ELSA is proportionate to that at population level. As such, transition probabilities obtained from SHARE and ELSA are a weighted average of the transition probabilities across the spectrum of the severity of the conditions.<br>The weighted average transition probability multiplied by the total number of people in a health state is mathematically equivalent to the sum of the product of severity specific transition probabilities and severity specific numbers of people in that health state.
Assumption 8: The effects of comorbidity (such as diabetes) are accounted for in the model.	Since SHARE and ELSA participants are assumed to be a representative sample of the population after weighting (see above), estimates for risks of dementia, cardiovascular disease, functional impairment and death obtained from SHARE and ELSA reasonably represent a weighted average of risk levels across the spectrum of the severity of these conditions and comorbidities.
**Calendar trends:**<br>Transition probabilities (mortality rates and incidence of cardiovascular disease, dementia, and functional impairment) change over time.	
Assumption 9: For models involving expected mortality rates following pre-pandemic trends, it is assumed the observed downward calendar trend in mortality rates over the past two decades to 2019 would have continued to the future in the absence of the pandemic.	Data obtained from the EU statistics show that log-mortality rates followed steady and log-linear downward trends over the past two decades. We assumed the most likely scenario would be that these trends would have continued in the absence of the pandemic (Figure C).
Assumption 10: Age- and sex-specific to general population risk ratio of COVID-19 mortality across European countries are similar to that for the UK.	Age- and sex-specific COVID-19 mortality rates for 2020-2022 were not available for all countries. Increased case fatality rate due to age is likely to have a similar relative risk compared to general population rates across countries.
Assumption 11: Trends in incidence of cardiovascular disease and dementia across the different European countries are parallel	Age and sex standardised cardiovascular incidence and mortality rates, as well as incidence of Alzheimer's disease as a proxy for dementia, declined in parallel (Figure E) between countries.
Assumption 13: The most likely net effect of future changes in risk factors would have been the continuation of calendar trends in mortality rates and incidence of dementia and cardiovascular disease observed over the past two decades prior to the pandemic, had the pandemic not happened.	Population levels of risk factors affecting incidence of cardiovascular disease and dementia such as diabetes, smoking, diet, and physical activity have changed over time. The net effect of changes in risk factors on changes in mortality rates and incidence of cardiovascular disease and dementia has been steady and linear declining calendar trends in the years leading to the pandemic.

Table B: Numbers and characteristics of study participants in each country

	Total number of study participants	Baseline					
		Survey Year	N	Age Mean (SD)	Sex N Men (%)	Current smokers N (%)	Cardiovascular Disease N (%)
**Austria**	8,642	2004	2,081	64.7 (10.0)	644 (41.2%)	275 (17.6%)	202 (12.9%)
**Belgium**	14,818	2004	5,049	63.8 (10.6)	1734 (45.5%)	682 (17.9%)	613 (16.1%)
**Czech Republic**	12,021	2006	3,723	63.4 (10.0)	1681 (45.2%)	1,621 (59.8%)	483 (17.7%)
**Denmark**	7,959	2004	2,025	63.2 (11.2)	940 (46.4%)	538 (26.6%)	221 (13.0%)
**Estonia**	10,868	2010	9,044	65.8 (10.3)	3,927 (43.4%)	1,377 (20.1%)	1,890 (20.9%)
**France**	11,829	2004	4,126	63.7 (11.2)	1,903 (46.1%)	446 (14.6%)	475 (15.2%)
**Germany**	11,795	2004	3,920	63.6 (9.8)	1,858 (47.4%)	524 (17.5%)	414 (13.8%)
**Greece**	9,956	2004	4,303	62.8 (11.4)	1,947 (45.3%)	761 (26.3%)	405 (14.0%)
**Hungary**	4,610	2010	4,422	64.2 (9.8)	2,022 (45.7%)	689 (22.6%)	783 (25.5%)
**Italy**	13,812	2004	4,417	64.0 (9.3)	2,167 (49.1%)	453 (17.8%)	321 (12.6%)
**Netherlands**	9,020	2004	3,953	62.7 (10.1)	1,906 (48.2%)	712 (24.1%)	406 (13.7%)
**Poland**	11,773	2006	5,193	63.5 (10.2)	2,568 (49.5%)	629 (25.7%)	592 (24.2%)
**Portugal**	3,547	2010	3,246	64.3 (10.0)	1,533 (47.2%)	209 (10.5%)	288 (14.3%)
**Slovenia**	8,871	2010	4,849	65.0 (10.2)	2,317 (47.8%)	393 (14.4%)	458 (16.7%)
**Spain**	14,182	2004	4,432	66.0 (10.9)	2,159 (48.7%)	361 (15.7%)	311 (13.4%)
**Sweden**	8,761	2004	4,006	64.5 (10.5)	1,926 (48.1%)	509 (16.7%)	569 (18.7%)
**Switzerland**	6,610	2004	1,303	63.9 (11.3)	608 (46.7%)	203 (20.4%)	93 (9.3%)
**United Kingdom**	17,906	2002	12,085	64.2 (11.0)	5,332 (44%)	2,159 (18.2%)	1,804 (14.9 %)

Table C: Weighted average of age and sex specific baseline prevalence estimates for each health state in the model across all countries included in the study for illustration purposes. Actual inputs to the model were country-specific.

Age group	No CVD, FI, CI, or Dementia State 1	CVD State 2	CVD+CI State 3	CI State 4	CVD+FI State 5	Dementia + CVD (CVD+CI+FI) State 6	Dementia (CI+FI) State 7	FI (no CVD or Dementia) State 8
**Men**								
35 - 39	97.8%	1.8%	-	-	0.0%	-	-	0.4%
40 - 44	94.2%	3.5%	-	-	0.3%	-	-	2.1%
45 - 49	94.2%	3.2%	-	-	0.4%	-	-	2.2%
50 - 54	78.0%	5.8%	1.0%	10.9%	0.8%	0.3%	0.9%	2.3%
55 - 59	75.4%	9.4%	1.2%	8.6%	1.0%	0.5%	0.8%	2.9%
60 - 64	70.2%	12.9%	1.8%	8.3%	1.7%	0.7%	1.1%	3.3%
65 - 69	64.2%	16.9%	2.4%	8.5%	2.6%	1.2%	1.2%	3.1%
70 - 74	55.9%	20.2%	3.7%	9.1%	3.3%	2.3%	2.0%	3.5%
75 - 79	44.7%	23.2%	6.0%	10.0%	4.6%	4.1%	3.3%	4.0%
80 - 84	34.4%	23.1%	7.7%	10.7%	6.0%	7.5%	5.4%	5.0%
85 - 89	27.0%	20.6%	9.7%	11.4%	7.6%	11.0%	8.1%	4.7%
90+	19.7%	17.8%	10.4%	11.8%	7.6%	14.3%	11.8%	6.5%
**Women**								
35 - 39	97.8%	1.8%	-	-	0.0%	-	-	0.4%
40 - 44	94.2%	3.5%	-	-	0.3%	-	-	2.1%
45 - 49	94.2%	3.2%	-	-	0.4%	-	-	2.2%
50 - 54	85.1%	3.7%	0.4%	6.4%	0.5%	0.1%	0.6%	3.2%
55 - 59	82.8%	6.0%	0.5%	5.4%	1.0%	0.3%	0.7%	3.4%
60 - 64	79.1%	8.3%	0.8%	5.4%	1.5%	0.4%	1.0%	3.6%
65 - 69	74.0%	11.3%	1.1%	5.7%	1.9%	0.8%	1.3%	3.9%
70 - 74	63.9%	15.5%	1.7%	6.7%	3.2%	1.8%	2.2%	5.0%
75 - 79	51.1%	17.9%	3.3%	8.7%	5.1%	3.6%	4.6%	5.8%
80 - 84	36.7%	17.9%	5.2%	10.6%	7.2%	6.8%	8.0%	7.6%
85 - 89	26.2%	17.3%	5.9%	10.4%	8.1%	11.7%	12.1%	8.3%
90+	17.5%	13.2%	8.3%	10.6%	8.9%	15.1%	17.3%	9.1%

*CVD: Cardiovascular disease; CI: Cognitive impairment; FI: Functional impairment

Figure A: Weighted average of transition probabilities to cardiovascular disease, cognitive impairment, dementia, and functional impairment by age, across all countries for illustration purposes. Actual inputs to the model were country-specific. Red lines represent values in Women and blue lines represent values in Men. Dashed lines represent point estimates ± standard errors.

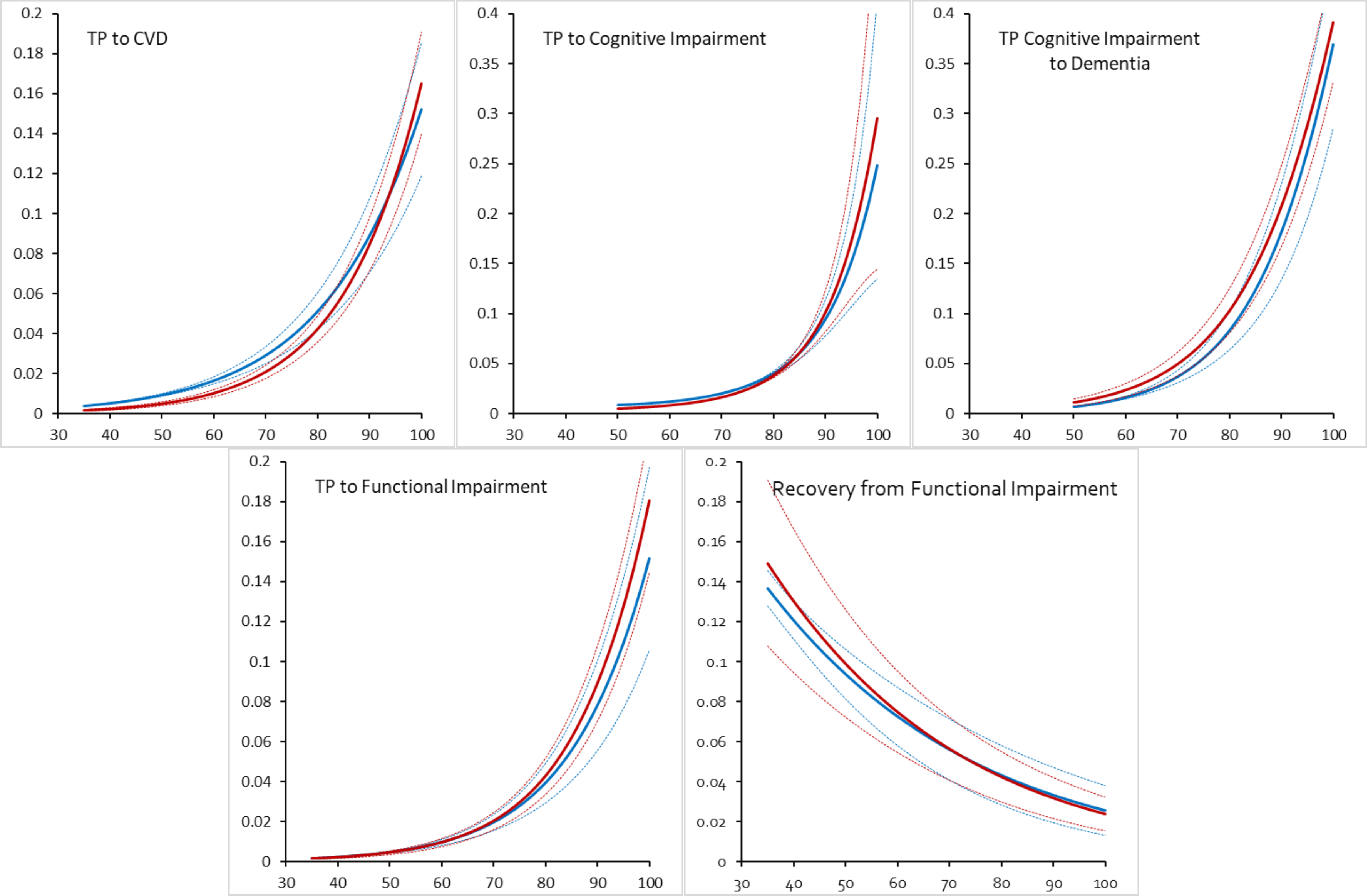

Figure B: Age and sex specific projections for mortality rates by year. Solid lines represent observed and dashed lines represent predicted rates. Y axis is on the logarithmic scale.

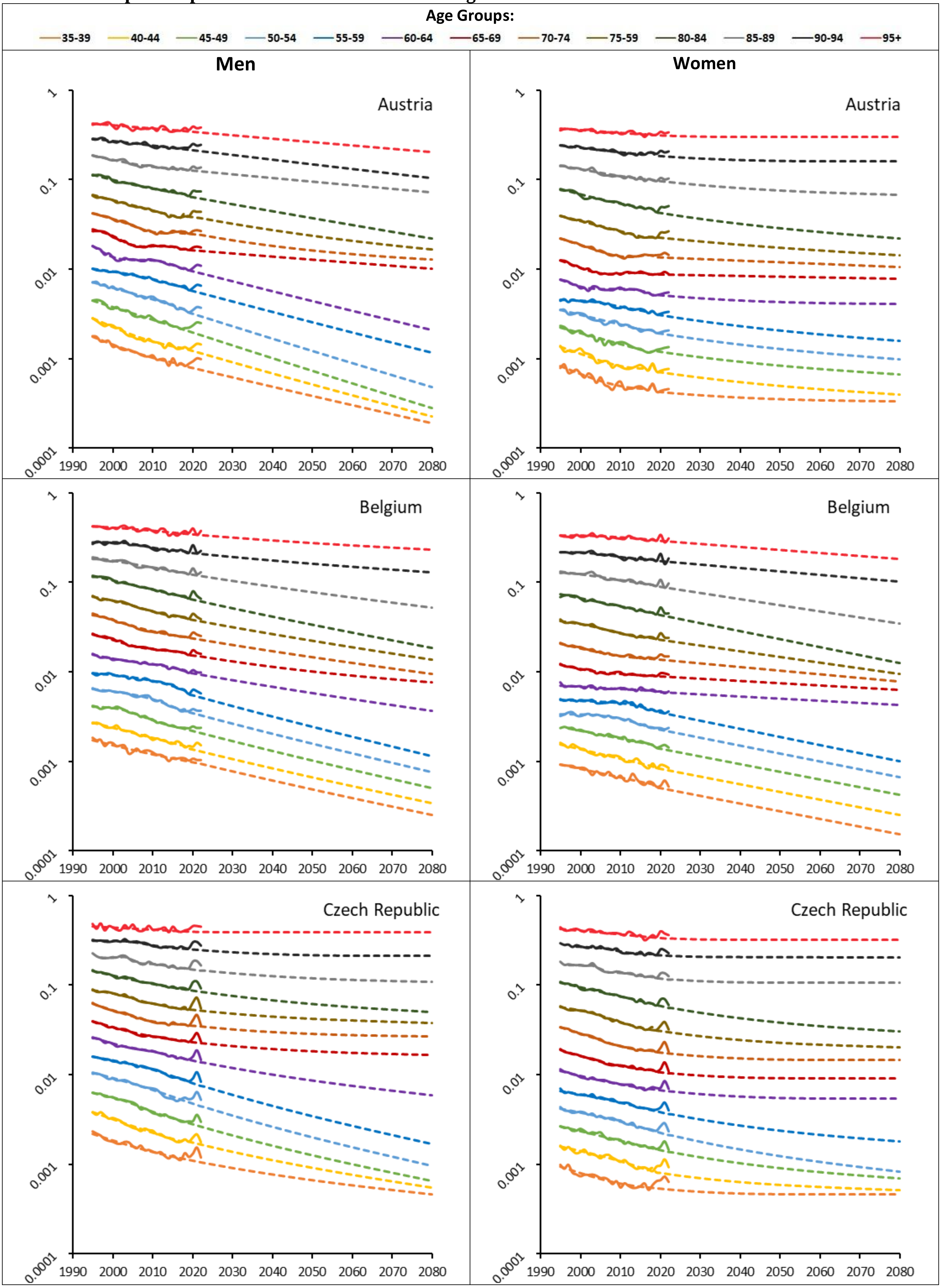

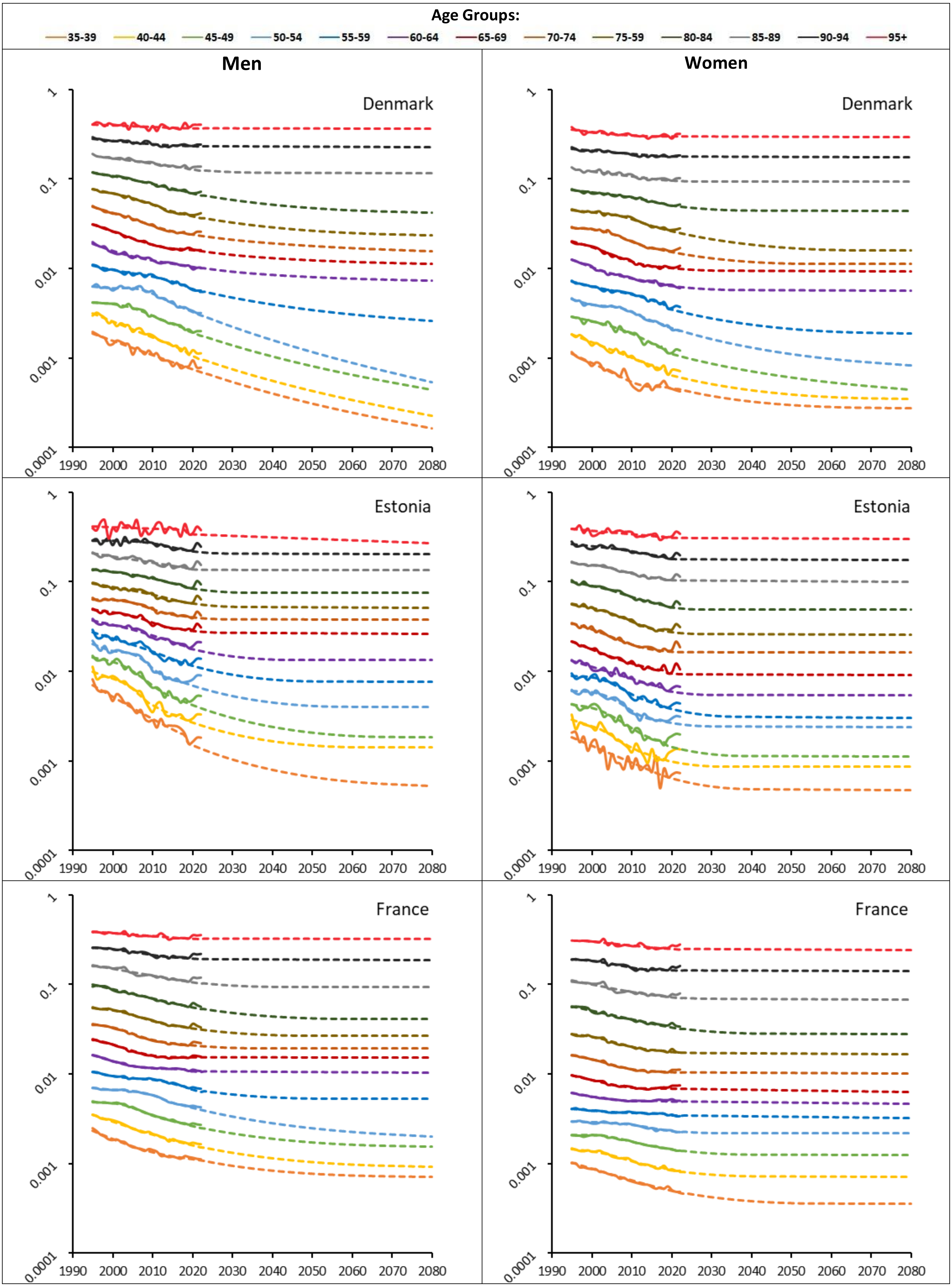

Age Groups:
35-39
40-44
45-49
50-54
55-59
60-64
65-69
70-74
75-59
80-84
85-89
90-94
95+
Men
Women
Denmark
Estonia
France
1
0.1
0.01
0.001
0.0001
1990
2000
2010
2020
2030
2040
2050
2060
2070
2080

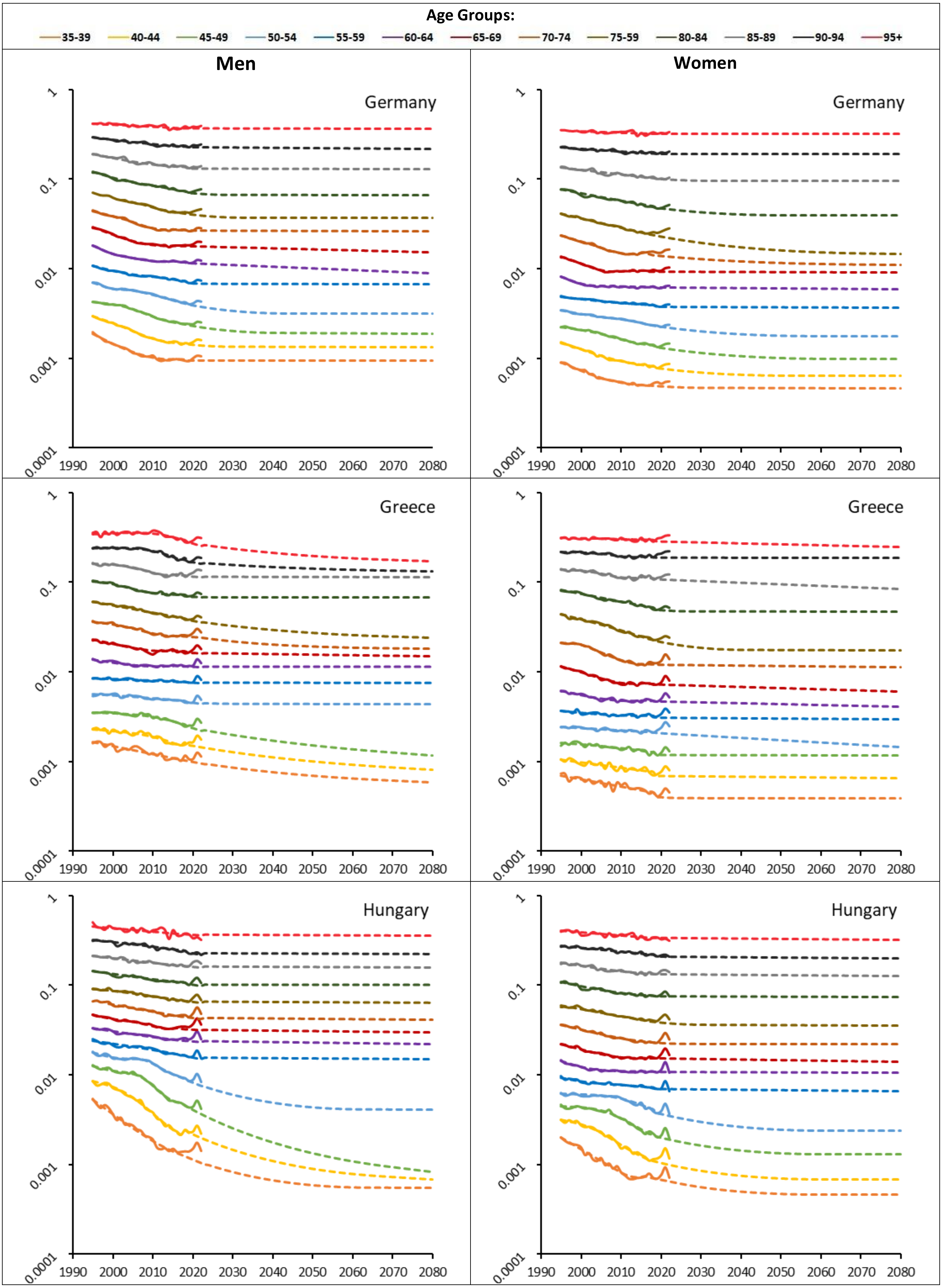
Age Groups:
35-39
40-44
45-49
50-54
55-59
60-64
65-69
70-74
75-59
80-84
85-89
90-94
95+
Men
Women
Germany
Germany
Greece
Greece
Hungary
Hungary
1
0.1
0.01
0.001
0.0001
1990
2000
2010
2020
2030
2040
2050
2060
2070
2080

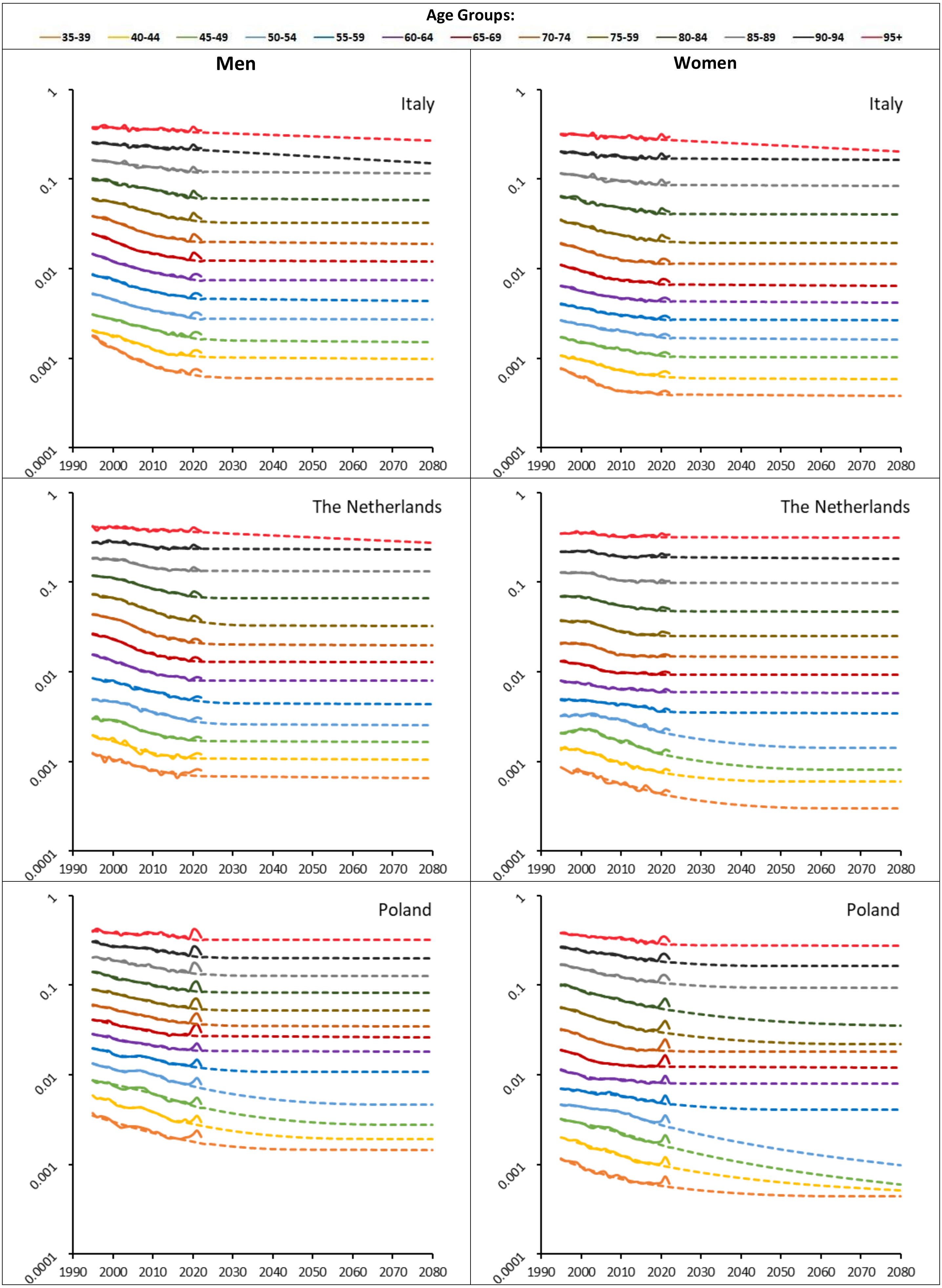
Age Groups:
35-39
40-44
45-49
50-54
55-59
60-64
65-69
70-74
75-59
80-84
85-89
90-94
95+
Men
Women
Italy
Italy
The Netherlands
The Netherlands
Poland
Poland
1
0.1
0.01
0.001
0.0001
1990
2000
2010
2020
2030
2040
2050
2060
2070
2080

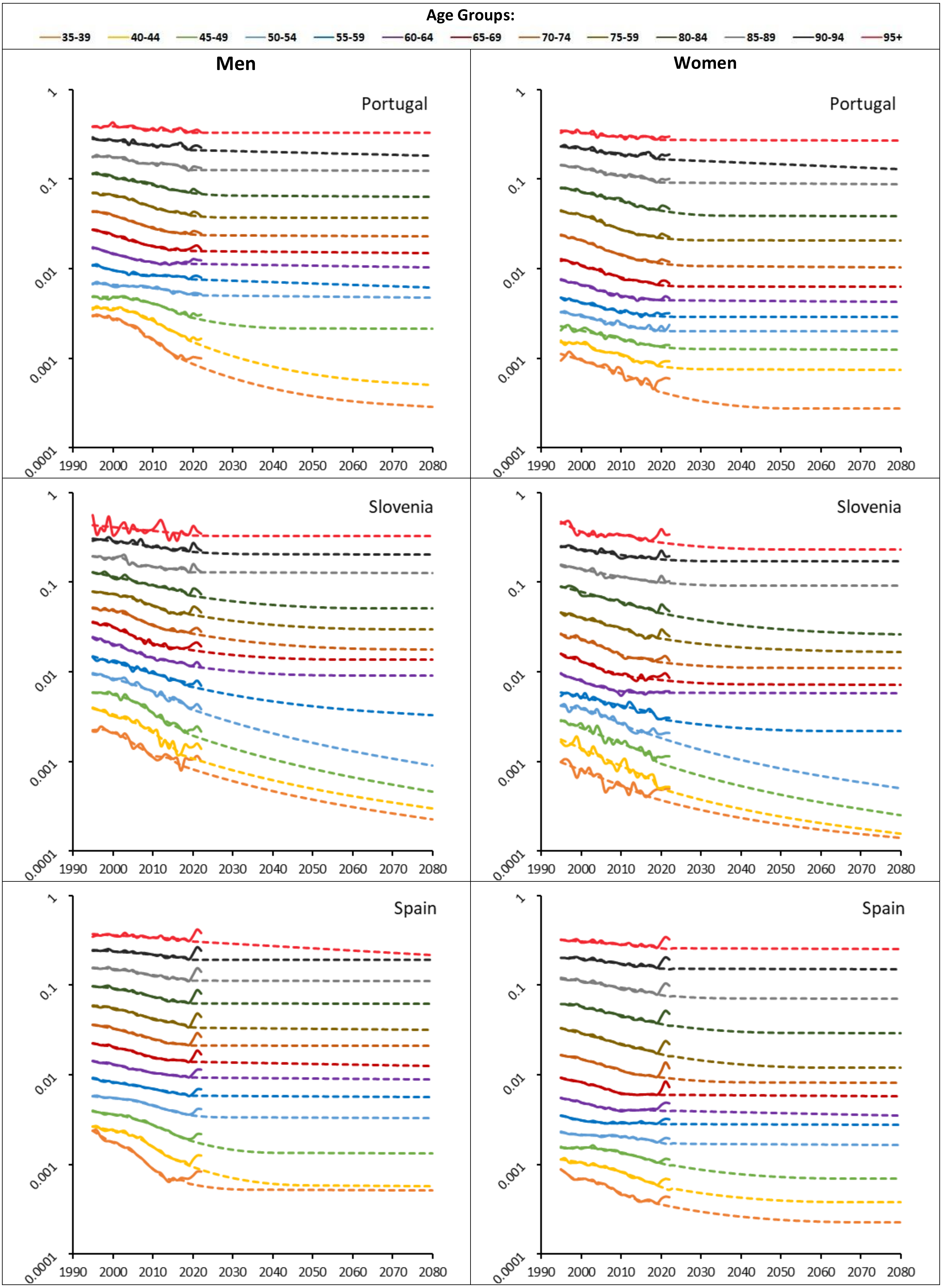
Age Groups:
35-39
40-44
45-49
50-54
55-59
60-64
65-69
70-74
75-59
80-84
85-89
90-94
95+
Men
Women
Portugal
Slovenia
Spain
1
0.1
0.01
0.001
0.0001
1990
2000
2010
2020
2030
2040
2050
2060
2070
2080

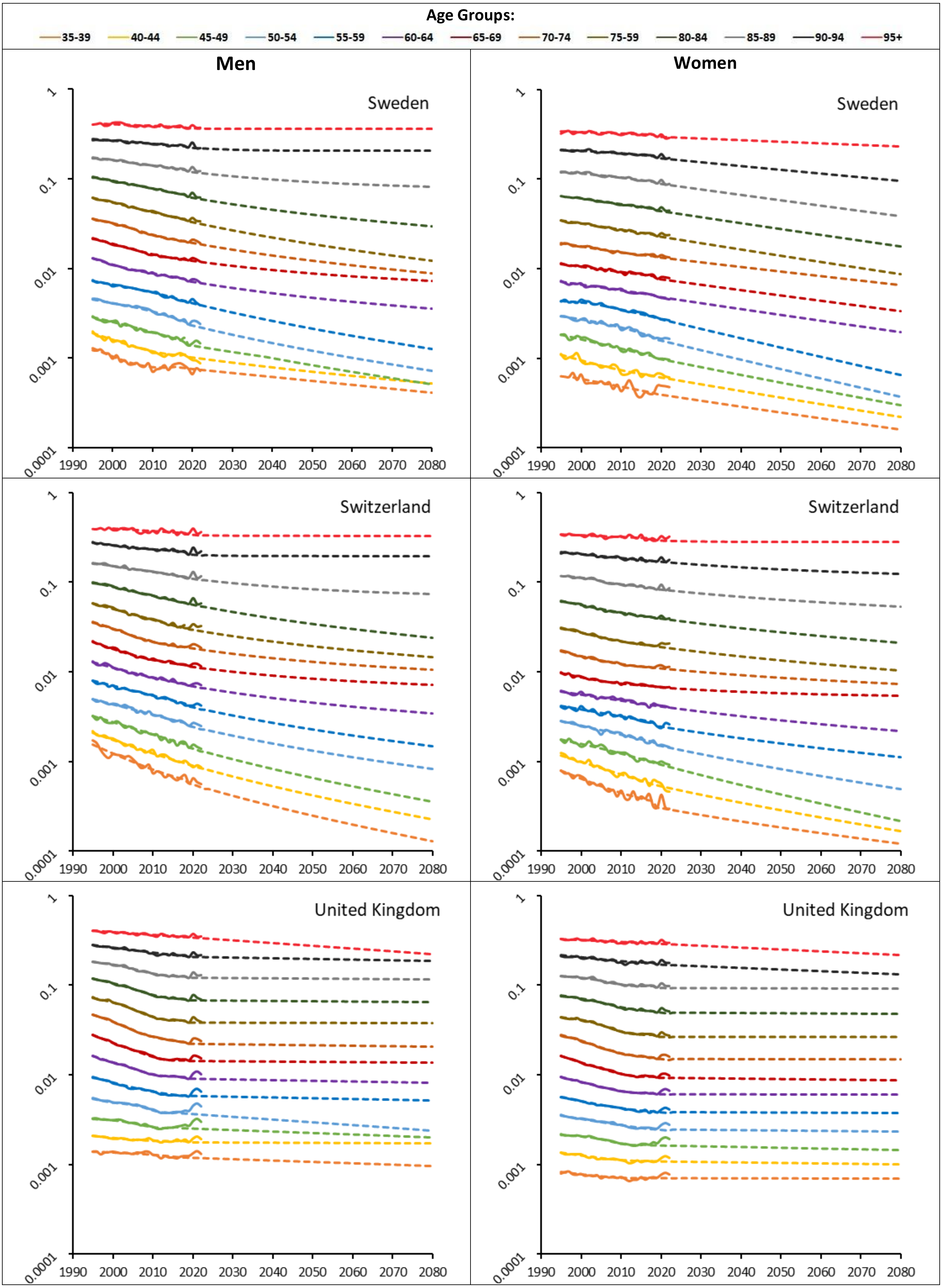
Age Groups:
35-39
40-44
45-49
50-54
55-59
60-64
65-69
70-74
75-59
80-84
85-89
90-94
95+
Men
Women
Sweden
Sweden
Switzerland
Switzerland
United Kingdom
United Kingdom
1
0.1
0.01
0.001
0.0001
1990
2000
2010
2020
2030
2040
2050
2060
2070
2080

Table D: COVID-19 mortality rates according to reported numbers of COVID-19 deaths 2020-2022 [19].

	COVID-19 Mortality Rates Per 100,000 Population		
	2020	2021	2022
Austria	84.8	104.1	51.6
Belgium	171.5	76.6	42.9
Czech Republic	110.0	229.9	55.9
Denmark	22.8	33.6	77.3
Estonia	17.8	129.8	69.8
France	97.8	89.3	57.6
Germany	38.9	92.5	58.7
Greece	47.2	154.5	134.8
Hungary	99.9	305.1	96.2
Italy	123.7	104.3	78.1
The Netherlands	67.4	55.2	12.0
Poland	75.3	178.5	54.6
Portugal	65.5	112.7	63.3
Slovenia	131.5	138.3	68.2
Spain	110.5	83.8	60.2
Sweden	85.4	64.4	63.8
Switzerland	90.6	48.7	22.3
United Kingdom	142.5	121.6	57.6

Table E: Relative risks of mortality from each state compared to the general population, a weighted average of all countries modelled in the study for illustration purposes only. Actual inputs to the Markov models were country-specific.

Age	No CVD, FI, CI, or Dementia S1	CVD S2	CVD+CI S3	CI S4	CVD+FI S5	Dementia + CVD (CVD+CI+FI) S6	Dementia (CI+FI) S7	FI (no CVD or Dementia) S8
**Men**								
35	0.999	2.97	3.76	2.72	11.59	13.27	11.80	12.21
36	0.999	2.87	3.64	2.63	11.21	12.84	11.41	11.80
37	0.999	2.77	3.52	2.54	10.84	12.41	11.03	11.41
38	0.998	2.68	3.40	2.46	10.48	12.00	10.67	11.04
39	0.998	2.60	3.29	2.38	10.13	11.60	10.31	10.67
40	0.998	2.51	3.18	2.30	9.80	11.22	9.97	10.32
41	0.997	2.43	3.08	2.23	9.47	10.85	9.64	9.98
42	0.996	2.35	2.98	2.15	9.16	10.49	9.32	9.65
43	0.995	2.27	2.88	2.08	8.86	10.14	9.01	9.33
44	0.994	2.20	2.79	2.01	8.56	9.80	8.71	9.02
45	0.993	2.12	2.70	1.95	8.28	9.48	8.43	8.72
46	0.991	2.06	2.61	1.88	8.00	9.16	8.14	8.43
47	0.989	1.99	2.52	1.82	7.74	8.85	7.87	8.15
48	0.986	1.92	2.44	1.76	7.48	8.56	7.61	7.87
49	0.98	1.86	2.36	1.70	7.23	8.27	7.36	7.61
50	0.98	1.80	2.28	1.65	6.99	7.99	7.11	7.36
51	0.97	1.74	2.21	1.59	6.75	7.73	6.87	7.11
52	0.97	1.68	2.13	1.54	6.53	7.47	6.64	6.87
53	0.96	1.63	2.06	1.49	6.31	7.21	6.42	6.64
54	0.95	1.57	2.00	1.44	6.10	6.97	6.20	6.42
55	0.94	1.52	1.93	1.40	5.89	6.73	5.99	6.20
56	0.91	1.47	1.87	1.35	5.69	6.50	5.79	5.99
57	0.88	1.42	1.81	1.31	5.50	6.28	5.60	5.79
58	0.85	1.38	1.75	1.26	5.31	6.07	5.41	5.59
59	0.82	1.33	1.69	1.22	5.13	5.86	5.22	5.40
60	0.80	1.29	1.63	1.18	4.96	5.66	5.04	5.21
61	0.77	1.25	1.58	1.14	4.79	5.46	4.87	5.04
62	0.74	1.21	1.53	1.11	4.62	5.28	4.70	4.86
63	0.72	1.17	1.48	1.07	4.46	5.09	4.54	4.70
64	0.70	1.13	1.43	1.04	4.31	4.92	4.39	4.53
65	0.67	1.09	1.38	1.00	4.16	4.74	4.23	4.38
66	0.65	1.06	1.34	0.97	4.02	4.58	4.09	4.22
67	0.63	1.02	1.29	0.94	3.88	4.42	3.94	4.08
68	0.61	0.99	1.25	0.91	3.74	4.26	3.81	3.93
69	0.59	0.96	1.21	0.88	3.61	4.11	3.67	3.79
70	0.57	0.93	1.17	0.85	3.48	3.96	3.54	3.66
71	0.56	0.90	1.13	0.82	3.36	3.82	3.42	3.53
72	0.54	0.87	1.10	0.80	3.24	3.68	3.30	3.40
73	0.52	0.84	1.06	0.77	3.13	3.55	3.18	3.28
74	0.50	0.81	1.03	0.75	3.02	3.42	3.06	3.16
75	0.49	0.79	0.99	0.72	2.91	3.29	2.95	3.05
76	0.47	0.76	0.96	0.70	2.80	3.17	2.85	2.94
77	0.46	0.74	0.93	0.68	2.70	3.05	2.74	2.83
78	0.45	0.72	0.90	0.66	2.60	2.94	2.64	2.73

79	0.43	0.70	0.88	0.64	2.51	2.83	2.55	2.63
80	0.42	0.67	0.85	0.62	2.42	2.72	2.45	2.53
81	0.41	0.65	0.82	0.60	2.33	2.62	2.36	2.44
82	0.40	0.63	0.80	0.58	2.24	2.52	2.28	2.34
83	0.39	0.62	0.77	0.57	2.16	2.42	2.19	2.26
84	0.37	0.60	0.75	0.55	2.08	2.33	2.11	2.17
85	0.36	0.58	0.73	0.54	2.00	2.24	2.03	2.09
86	0.36	0.57	0.71	0.52	1.93	2.15	1.96	2.01
87	0.35	0.55	0.69	0.51	1.86	2.07	1.88	1.94
88	0.34	0.54	0.67	0.49	1.79	1.99	1.81	1.86
89	0.33	0.52	0.65	0.48	1.72	1.91	1.75	1.79
90	0.32	0.51	0.64	0.47	1.66	1.84	1.68	1.73
91	0.32	0.50	0.62	0.46	1.60	1.77	1.62	1.66
92	0.31	0.49	0.61	0.45	1.54	1.70	1.56	1.60
93	0.31	0.48	0.60	0.44	1.49	1.63	1.51	1.54
94	0.30	0.47	0.58	0.44	1.44	1.57	1.45	1.49
95	0.30	0.46	0.57	0.43	1.39	1.51	1.40	1.43
96	0.29	0.46	0.56	0.42	1.34	1.46	1.35	1.38
97	0.29	0.45	0.56	0.42	1.30	1.40	1.31	1.34
98	0.29	0.45	0.55	0.41	1.26	1.35	1.27	1.29
99	0.29	0.44	0.54	0.41	1.22	1.31	1.23	1.25
100	0.29	0.44	0.54	0.41	1.18	1.27	1.19	1.21

Age	S1	S2	S3	S4	S5	S6	S7	S10
**Women**								
35	0.999	2.75	3.49	2.52	10.77	12.33	10.96	11.34
36	0.998	2.66	3.38	2.44	10.41	11.93	10.60	10.97
37	0.998	2.57	3.27	2.36	10.07	11.54	10.25	10.61
38	0.997	2.49	3.16	2.28	9.74	11.16	9.91	10.26
39	0.997	2.41	3.06	2.21	9.42	10.79	9.59	9.92
40	0.996	2.33	2.96	2.14	9.11	10.43	9.27	9.59
41	0.995	2.25	2.86	2.07	8.81	10.09	8.96	9.28
42	0.993	2.18	2.77	2.00	8.52	9.76	8.67	8.97
43	0.992	2.11	2.67	1.93	8.24	9.43	8.38	8.67
44	0.990	2.04	2.59	1.87	7.96	9.12	8.11	8.39
45	0.987	1.97	2.50	1.81	7.70	8.82	7.84	8.11
46	0.984	1.91	2.42	1.75	7.45	8.53	7.58	7.84
47	0.980	1.85	2.34	1.69	7.20	8.25	7.33	7.58
48	0.975	1.78	2.26	1.64	6.96	7.97	7.09	7.33
49	0.97	1.73	2.19	1.58	6.73	7.71	6.85	7.09
50	0.96	1.67	2.12	1.53	6.51	7.45	6.62	6.85
51	0.95	1.62	2.05	1.48	6.29	7.20	6.40	6.63
52	0.94	1.56	1.98	1.43	6.08	6.96	6.19	6.41
53	0.92	1.51	1.92	1.38	5.88	6.73	5.99	6.19
54	0.90	1.46	1.85	1.34	5.69	6.51	5.79	5.99
55	0.87	1.41	1.79	1.30	5.50	6.29	5.59	5.79
56	0.84	1.37	1.73	1.25	5.31	6.08	5.41	5.59
57	0.82	1.32	1.68	1.21	5.14	5.87	5.23	5.41
58	0.79	1.28	1.62	1.17	4.96	5.68	5.05	5.23
59	0.76	1.24	1.57	1.13	4.80	5.49	4.88	5.05
60	0.74	1.20	1.52	1.10	4.64	5.30	4.72	4.88
61	0.71	1.16	1.47	1.06	4.48	5.12	4.56	4.72

62	0.69	1.12	1.42	1.03	4.33	4.95	4.41	4.56
63	0.67	1.08	1.37	0.99	4.18	4.78	4.26	4.40
64	0.65	1.05	1.33	0.96	4.04	4.62	4.11	4.25
65	0.63	1.01	1.28	0.93	3.91	4.46	3.97	4.11
66	0.61	0.98	1.24	0.90	3.77	4.31	3.84	3.97
67	0.59	0.95	1.20	0.87	3.65	4.16	3.71	3.83
68	0.57	0.92	1.16	0.84	3.52	4.02	3.58	3.70
69	0.55	0.89	1.13	0.81	3.40	3.88	3.46	3.58
70	0.53	0.86	1.09	0.79	3.29	3.75	3.34	3.45
71	0.51	0.83	1.05	0.76	3.17	3.62	3.23	3.34
72	0.50	0.81	1.02	0.74	3.06	3.49	3.12	3.22
73	0.48	0.78	0.99	0.72	2.96	3.37	3.01	3.11
74	0.47	0.75	0.95	0.69	2.86	3.25	2.90	3.00
75	0.45	0.73	0.92	0.67	2.76	3.14	2.80	2.90
76	0.44	0.71	0.89	0.65	2.66	3.03	2.71	2.80
77	0.42	0.69	0.87	0.63	2.57	2.92	2.61	2.70
78	0.41	0.66	0.84	0.61	2.48	2.82	2.52	2.60
79	0.40	0.64	0.81	0.59	2.39	2.72	2.43	2.51
80	0.39	0.62	0.79	0.57	2.31	2.62	2.35	2.42
81	0.37	0.60	0.76	0.55	2.23	2.53	2.27	2.34
82	0.36	0.59	0.74	0.54	2.15	2.43	2.19	2.26
83	0.35	0.57	0.72	0.52	2.08	2.35	2.11	2.18
84	0.34	0.55	0.69	0.51	2.00	2.26	2.04	2.10
85	0.33	0.53	0.67	0.49	1.93	2.18	1.96	2.02
86	0.32	0.52	0.65	0.48	1.86	2.10	1.89	1.95
87	0.31	0.50	0.63	0.46	1.80	2.03	1.83	1.88
88	0.31	0.49	0.62	0.45	1.74	1.95	1.76	1.82
89	0.30	0.48	0.60	0.44	1.68	1.88	1.70	1.75
90	0.29	0.46	0.58	0.43	1.62	1.81	1.64	1.69
91	0.28	0.45	0.57	0.42	1.56	1.75	1.59	1.63
92	0.28	0.44	0.55	0.41	1.51	1.68	1.53	1.57
93	0.27	0.43	0.54	0.40	1.46	1.62	1.48	1.52
94	0.27	0.42	0.53	0.39	1.41	1.57	1.43	1.47
95	0.26	0.41	0.52	0.38	1.36	1.51	1.38	1.42
96	0.26	0.41	0.51	0.37	1.32	1.46	1.34	1.37
97	0.25	0.40	0.50	0.37	1.28	1.41	1.29	1.33
98	0.25	0.39	0.49	0.36	1.24	1.36	1.25	1.28
99	0.25	0.39	0.48	0.36	1.20	1.32	1.21	1.24
100	0.24	0.38	0.47	0.35	1.17	1.28	1.18	1.21

*CVD: Cardiovascular disease; CI: Cognitive impairment; FI: Functional impairment

Figure C: Comparison of mortality rates expected in the absence of the pandemic with estimated actual mortality rates* with and without disability over 2020-2022. Data for ages 60 and 80 for three countries with higher, average, and lower mortality rates among the studied countries are presented as examples.

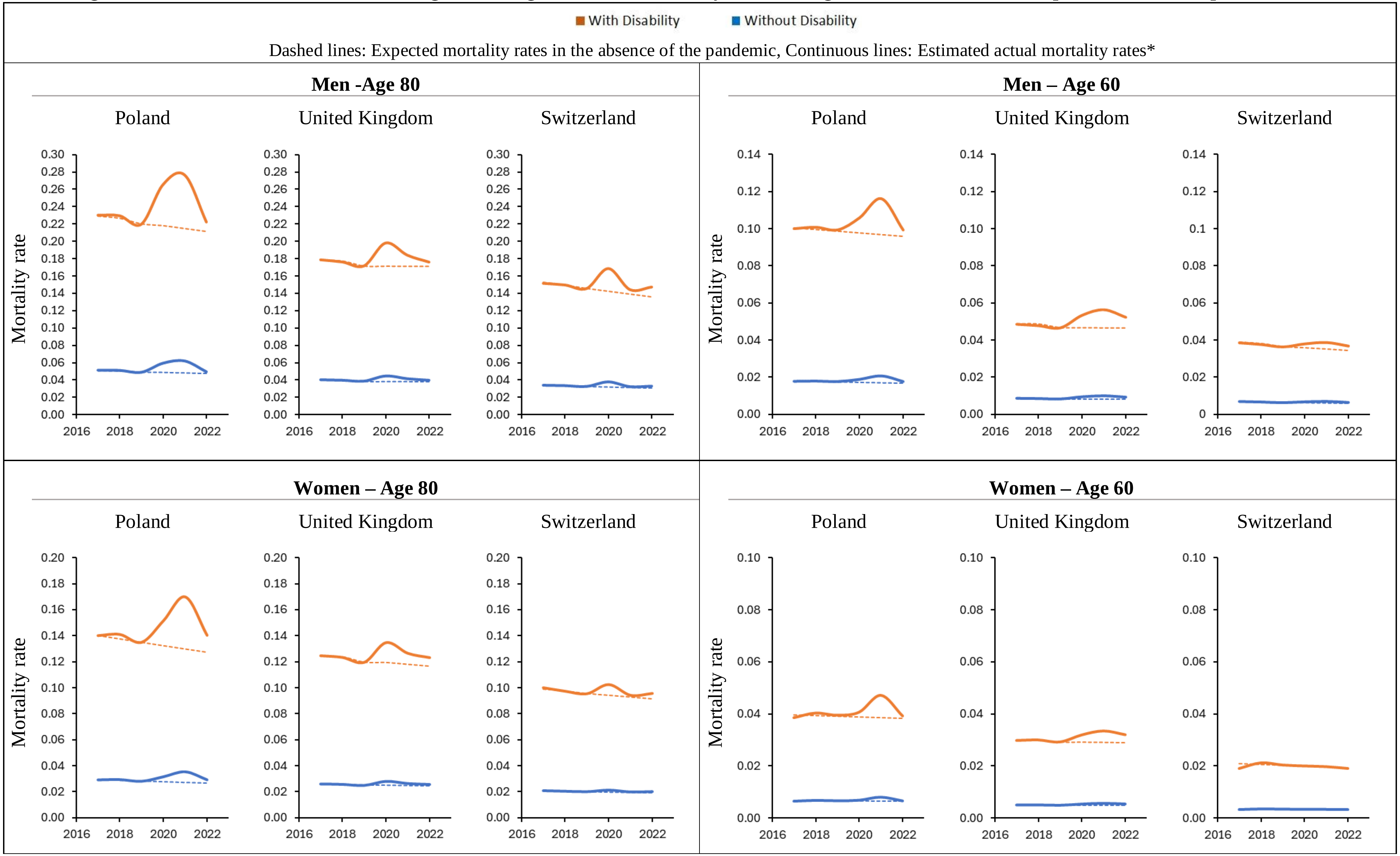

* We used observed all-cause mortality rates by age and sex and the hazard ratio of death with disability over all-cause mortality by age, sex, and country for the calculations. This method assumes the hazard of death by disability remains proportional to the baseline hazard of death (i.e., all-cause mortality by age and sex) over time.

Table F: Proportion of excess deaths in 2020-2022 that was estimated to occur with disability by age-group.

Excess mortality is defined as the difference between observed all-cause mortality and expected mortality by age-group. Expected mortality rates were estimated by projecting pre-pandemic mortality trends to the future.

	**Proportion of excess mortality associated with disability by age-group**			
	**35-49**	**50-64**	**65-79**	**80-100**
**All 18 countries**	11 %	24 %	44 %	65 %
**Switzerland**	5 %	13 %	31 %	54 %
**Sweden**	7 %	17 %	38 %	56 %
**Denmark**	9 %	21 %	42 %	61 %
**France**	9 %	21 %	43 %	66 %
**The Netherlands**	7 %	18 %	38 %	54 %
**Italy**	5 %	15 %	41 %	71 %
**Portugal**	14 %	27 %	45 %	55 %
**Germany**	13 %	27 %	47 %	68 %
**Belgium**	16 %	32 %	52 %	69 %
**United Kingdom**	18 %	35 %	50 %	60 %
**Austria**	6 %	19 %	43 %	68 %
**Greece**	2 %	16 %	39 %	55 %
**Slovenia**	7 %	17 %	41 %	67 %
**Czech Republic**	13 %	26 %	49 %	67 %
**Hungary**	19 %	26 %	38 %	63 %
**Spain**	5 %	16 %	45 %	75 %
**Poland**	18 %	30 %	42 %	67 %
**Estonia**	15 %	29 %	50 %	69 %

Figure D: Age-standardized all-cause and COVID-19 mortality rates in men and women in 2020 and 2021 in the United Kingdom by disease and disability status, comparing the model outputs with those reported by the Office for National Statistics*

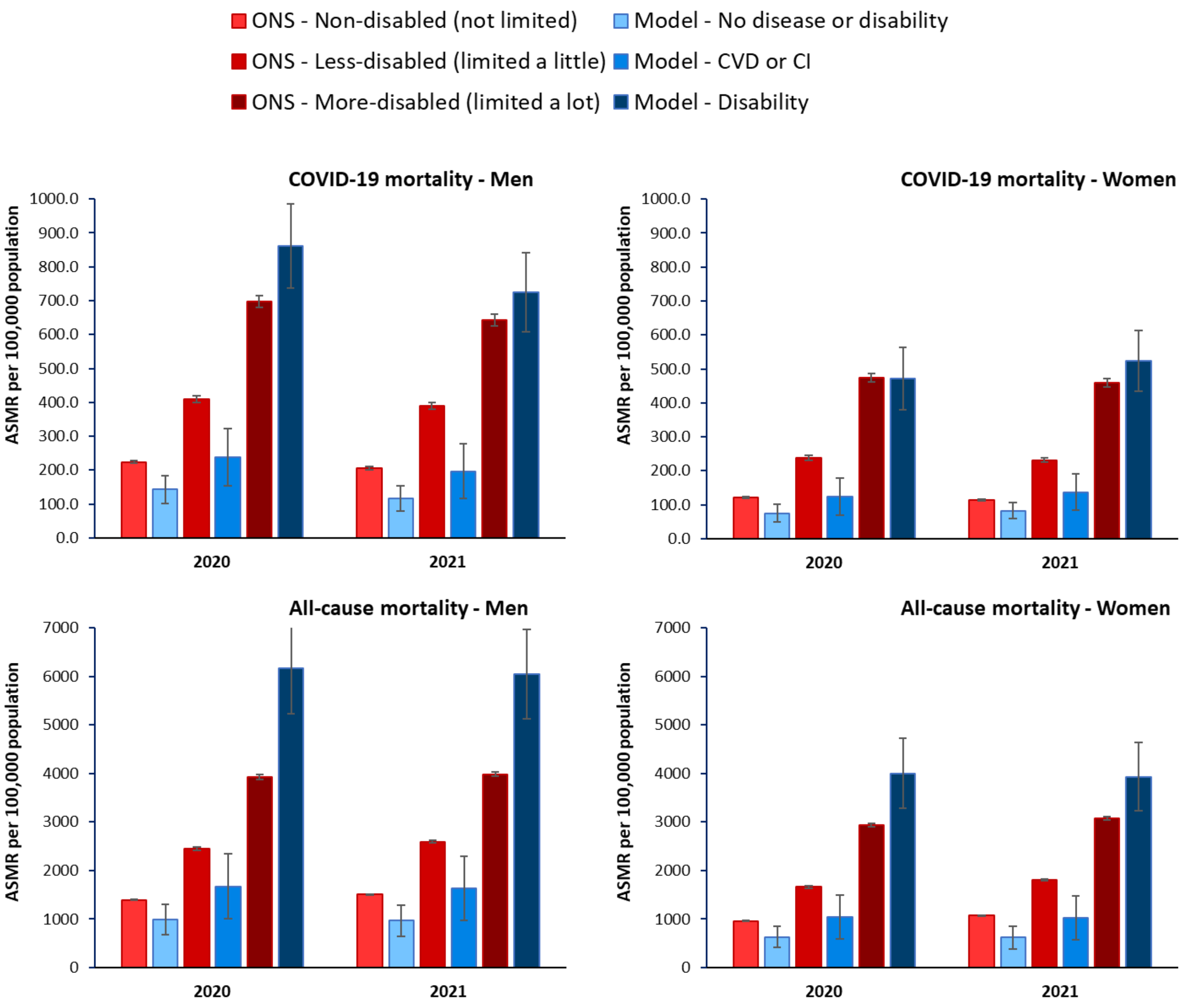

CVD: Cardiovascular disease. CI: Cognitive Impairment

* Disability is defined differently in the report by the Office for National Statistics **[17]** and in this study. In the report by the Office for National Statistics disability status was defined using the self-reported responses to the 2011 Census question; "Are your day-to-day activities limited because of a health problem or disability which has lasted, or is expected to last, at least 12 months? - Include problems related to old age" (Yes, limited a lot; Yes, limited a little; and No) **[17].** Disability in this study was defined based on the inability to independently perform one or more basic activities of daily living (ADLs).

Figure E: Age-standardised incidence of cardiovascular diseases and Alzheimer's disease across European countries over time. Source of data: Institute of Health Metrics and Evaluation [18]

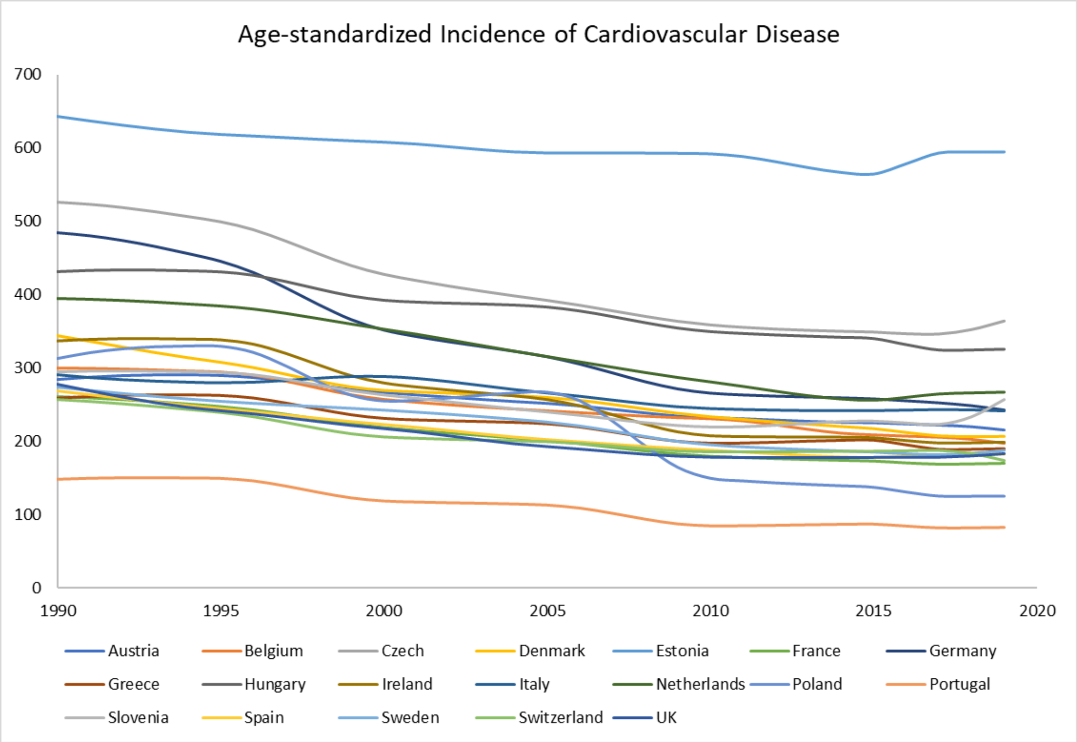

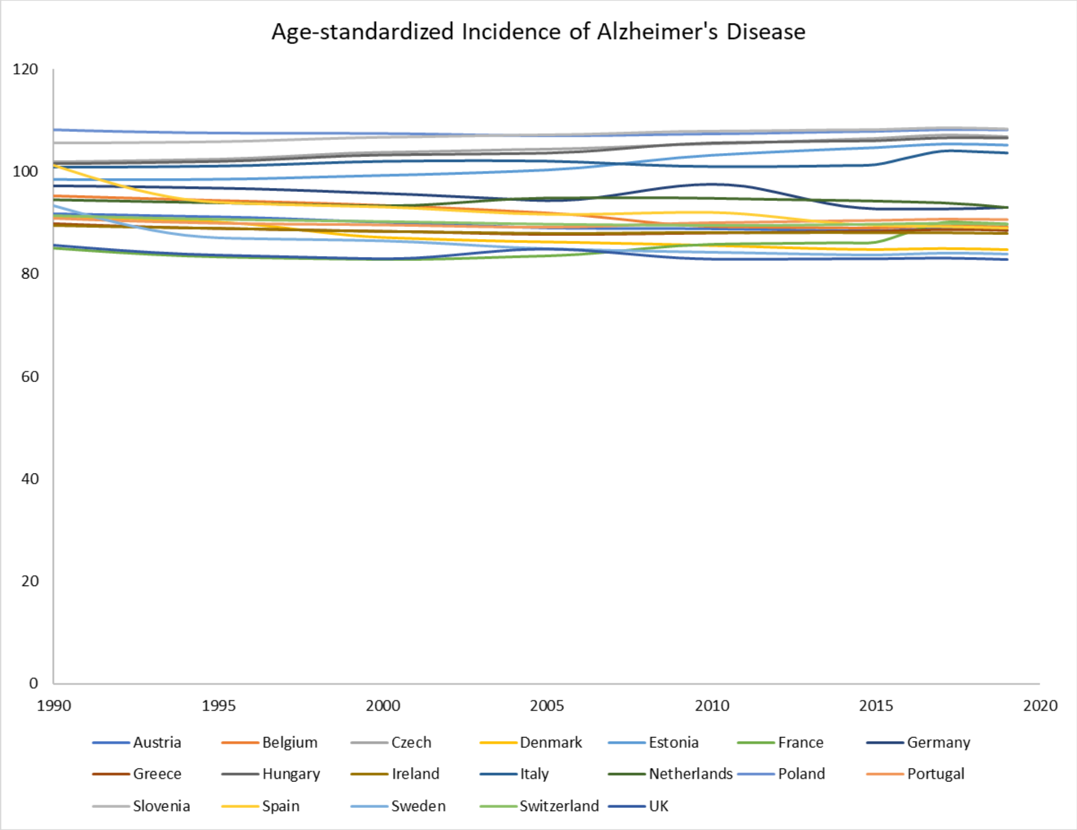

Appendix Results

Figure F: Person-years of life lost per 1000 population aged 35 and over, by country, explained and not explained by registered COVID deaths over the years 2020-2022 *

Error bars represent 95% Uncertainty Intervals obtained from Monte-Carlo simulation.

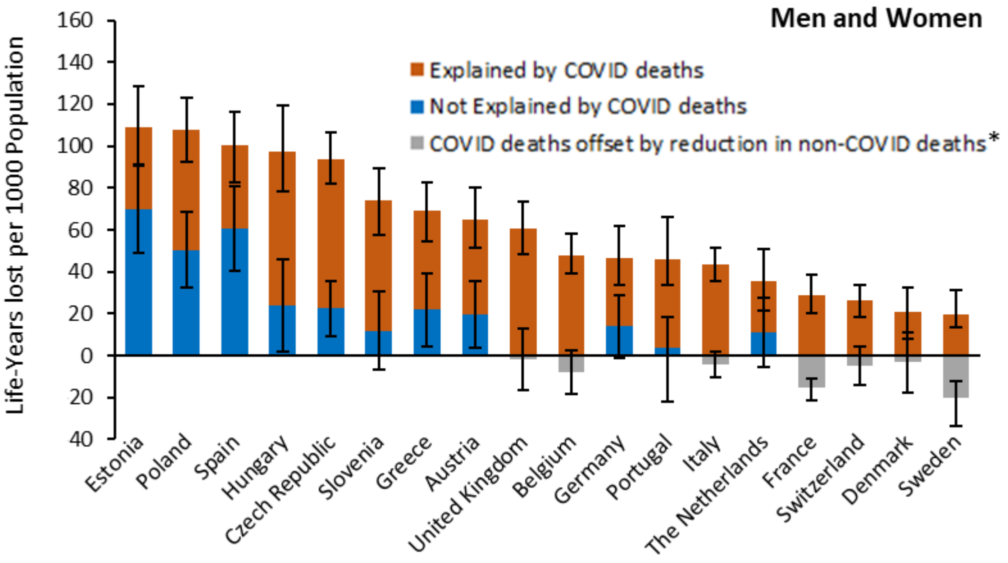

* **Grey bars** represent the opposing effect of loss in person-years of life attributed to COVID-19 deaths that are compensated by the observed lower than expected deaths from non-COVID causes in several countries at several timepoints, resulting in no net loss in person-years of life. There are two scenarios that explain this finding:

- If the observed lower than expected mortality rates from non-COVID causes is due to a true reduction in non-COVID deaths during the pandemic (Scenario 1), the PYLL due to COVID-19 would be the sum of the orange and grey bars. In this case a portion of PYLL due to COVID-19 is compensated by the lower than expected non-COVID mortality (the portion displayed by the grey bars), reducing the total PYLL.
- If the observed lower than expected mortality rates from non-COVID causes are due to replacement of expected non-COVID mortality by COVID-19 as registered cause of death in case of co-occurrence of morbidities (Scenario 2), the PYLL due to COVID-19 are represented by the orange bars. In this case the grey bars are mere representations of substitution of causes for “expected” deaths and does not represent PYLL.
- In both cases, PYLL due to non-COVID deaths are represented by the blue bars.

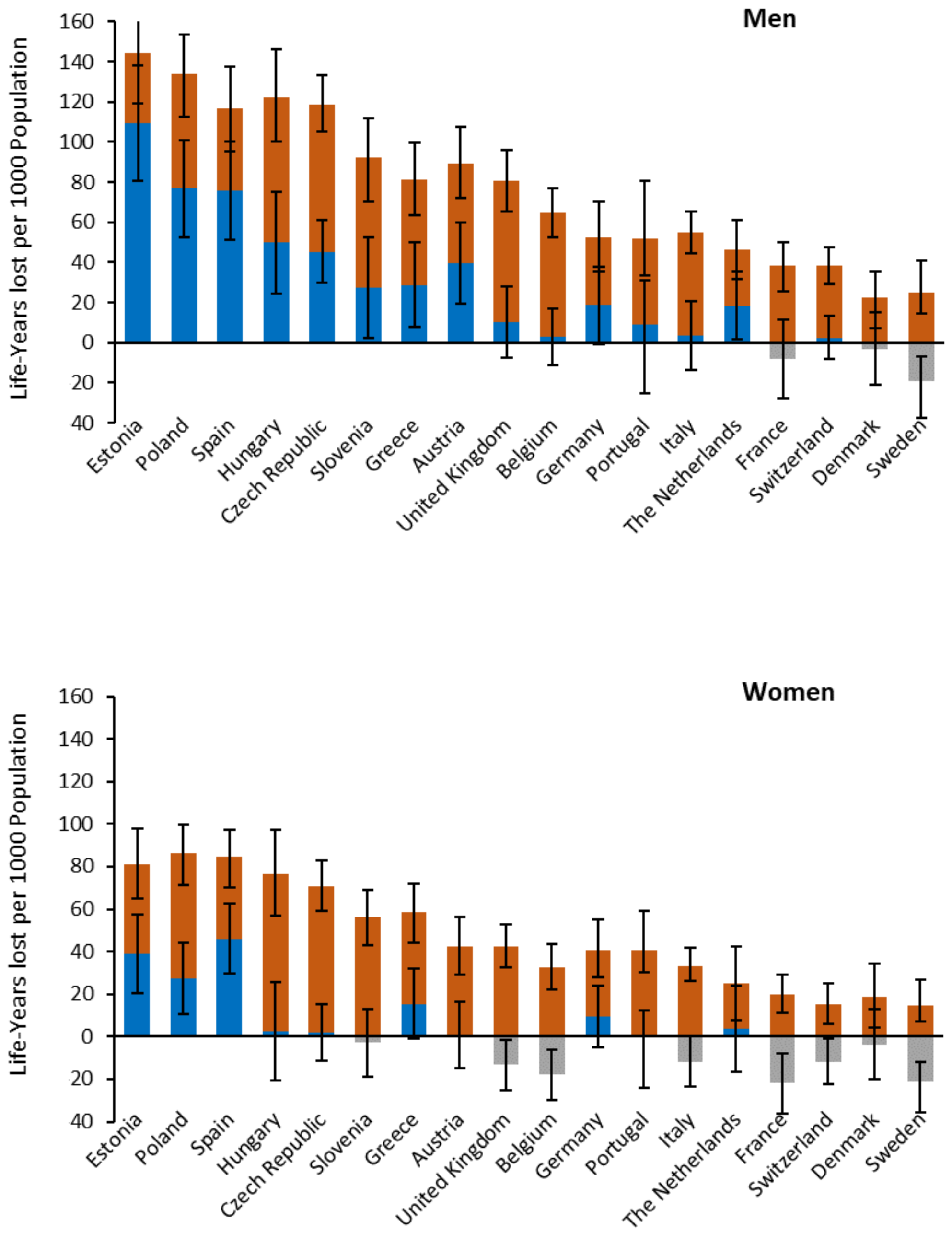
Men
Women
Life-Years lost per 1000 Population
160
140
120
100
80
60
40
20
0
20
40
Estonia
Poland
Spain
Hungary
Czech Republic
Slovenia
Greece
Austria
United Kingdom
Belgium
Germany
Portugal
Italy
The Netherlands
France
Switzerland
Denmark
Sweden

Table G: Person-years of life lost (PYLL) in the population aged 35 and over during the years 2020-2022, estimated by comparing Markov models with projected and observed all-cause mortality rates in thousands (000).

Numbers in parenthesis represent 95% Uncertainty Intervals obtained from Markov model Monte-Carlo simulations. Minus sign indicates life-years lost.

	Year		
	**2020 (000)**	**2021 (000)**	**2022 (000)**
Austria			
PYLL attributed to COVID-19	-81 (-114; -47)	-117 (-149; -96)	-48 (-94; -15)
PYLL attributed to other causes of death	-8 (-41; 26)	-7 (-29; 16)	-90 (-129; -50)
Belgium			
PYLL attributed to COVID-19	-210 (-239; -180)	-111 (-164; -68)	-52 (-119; 4)
PYLL attributed to other causes of death	63 (33; 92)	37 (2; 72)	-46 (-96; 4)
Czech Republic			
PYLL attributed to COVID-19	-119 (-155; -83)	-304 (-365; -255)	-60 (-147; 14)
PYLL attributed to other causes of death	-61 (-98; -24)	-66 (-124; -8)	-27 (-113; 60)
Denmark			
PYLL attributed to COVID-19	-13 (-33; 6)	-23 (-31; -21)	-46 (-60; -37)
PYLL attributed to other causes of death	9 (-11; 29)	-0.1 (-2; 2)	2 (-5; 10)
Estonia			
PYLL attributed to COVID-19	-2 (-9; 4)	-19 (-23; -17)	-9 (-15; -5)
PYLL attributed to other causes of death	-10 (-16; -4)	-21 (-24; -17)	-24 (-31; -16)
France			
PYLL attributed to COVID-19	-642 (-831; -452)	-681 (-896; -528)	-380 (-683; -144)
PYLL attributed to other causes of death	224 (130; 319)	356 (236; 475)	14 (-171; 199)
Germany			
PYLL attributed to COVID-19	-314 (-638; 9)	-869 (-1,073; -761)	-467 (-777; -269)
PYLL attributed to other causes of death	-24 (-348; 300)	-7 (-117; 102)	-670 (-907; -432)
Greece			
PYLL attributed to COVID-19	-45 (-91; 1)	-173 (-207; -151)	-130 (-190; -87)
PYLL attributed to other causes of death	-54 (-100; -8)	-72 (-102; -43)	-34 (-90; 22)
Hungary			
PYLL attributed to COVID-19	-78 (-133; -23)	-294 (-349; -254)	-75 (-157; -12)
PYLL attributed to other causes of death	-57 (-116; 1)	-62 (-109; -14)	-27 (-101; 48)
Italy			
PYLL attributed to COVID-19	-704 (-879; -529)	-684 (-909; -519)	-438 (-756; -182)
PYLL attributed to other causes of death	-27 (-207; 154)	61 (-103; 225)	31 (-220; 283)
Netherlands			
PYLL attributed to COVID-19	-121 (-188; -53)	-116 (-161; -94)	-21 (-77; 11)
PYLL attributed to other causes of death	20 (-48; 87)	-43 (-67; -20)	-90 (-128; -51)
Poland			
PYLL attributed to COVID-19	-287 (-441; -133)	-846 (-1,016; -722)	-214 (-461; -21)
PYLL attributed to other causes of death	-476 (-637; -315)	-529 (-720; -337)	-168 (-457; 121)
Portugal			
PYLL attributed to COVID-19	-71 (-121; -22)	-147 (-183; -125)	-70 (-124; -33)
PYLL attributed to other causes of death	-37 (-87; 13)	36 (14; 58)	-26 (-64; 12)
Slovenia			
PYLL attributed to COVID-19	-30 (-39; -21)	-38 (-48; -30)	-15 (-30; -4)
PYLL attributed to other causes of death	-9 (-18; 1)	-1 (-9; 8)	-7 (-19; 6)
Spain			
PYLL attributed to COVID-19	-512 (-739; -286)	-465 (-642; -365)	-283 (-522; -123)

PYLL attributed to other causes of death	-71 (-301; 158)	-968 (-1,133; -804)	-879 (-1,175; -583)
Sweden			
PYLL attributed to COVID-19	-89 (-113; -64)	-78 (-105; -58)	-66 (-106; -35)
PYLL attributed to other causes of death	32 (8; 57)	47 (33; 61)	38 (15; 60)
Switzerland			
PYLL attributed to COVID-19	-88 (-108; -69)	-56 (-80; -38)	-22 (-53; 2)
PYLL attributed to other causes of death	27 (8; 46)	21 (7; 36)	-22 (-43; -1)
United Kingdom			
PYLL attributed to COVID-19	-989 (-1,188; -790)	-990 (-1,300; -746)	-392 (-813; -41)
PYLL attributed to other causes of death	160 (60; 259)	113 (-117; 342)	-201 (-538; 136)

Table H: Disabled and disability-free person-years of life lost (PYLL) in the population aged 35 and over during the years 2020-2022, estimated by comparing Markov models with projected and observed all-cause mortality rates, in thousands (000).

Numbers in parenthesis represent 95% Uncertainty Intervals obtained from Monte-Carlo simulation. Minus sign indicates life-years lost.

			Person-Years of Life lost comparing projected and observed mortality in thousands (000)	Proportion of Person-Years of Life lost without disability
All 18 countries				
	Men and Women			
		Total PYLL	-16,819 (-21,781; -11,689)	
		PYLL *without* disability	-9,795 (-15,137; -4,665)	58%
		PYLL *with* disability	-7,024 (-10,627; -3,189)	
By Sex				
	Men			
		Total PYLL	-9,872 (-12,863; -6,851)	
		PYLL *without* disability	-6,369 (-9,601; -3,048)	65%
		PYLL *with* disability	-3,503 (-5,379; -1,552)	
	Women			
		Total PYLL	-6,946 (-9,410; -4,461)	
		PYLL *without* disability	-3,426 (-7,241; 231)	49%
		PYLL *with* disability	-3,520 (-6,002; -883)	
By Country				
	Austria			
		Total PYLL	-350 (-444; -244)	
		PYLL *without* disability	-201 (-307; -103)	57%
		PYLL *with* disability	-149 (-235; -73)	
	Belgium			
		Total PYLL	-319 (-403; -241)	
		PYLL *without* disability	-162 (-298; -22)	51%
		PYLL *with* disability	-156 (-258; -69)	
	Czech Republic			
		Total PYLL	-637 (-749; -540)	
		PYLL *without* disability	-376 (-513; -251)	59%
		PYLL *with* disability	-261 (-348; -185)	
	Denmark			
		Total PYLL	-71 (-140; -19)	
		PYLL *without* disability	-40 (-113; 36)	56%
		PYLL *with* disability	-31 (-81; 28)	
	Estonia			
		Total PYLL	-85 (-105; -67)	
		PYLL *without* disability	-54 (-70; -40)	64%
		PYLL *with* disability	-31 (-41; -19)	
	France			
		Total PYLL	-1,108 (-1,647; -538)	
		PYLL *without* disability	-569 (-1,270; 176)	51%
		PYLL *with* disability	-540 (-1,054; 25)	
	Germany			
		Total PYLL	-2,351 (-3,293; -1,320)	
		PYLL *without* disability	-1,284 (-2,239; -338)	55%
		PYLL *with* disability	-1,066 (-1,723; -432)	

Greece			
	Total PYLL	-508 (-648; -375)	
	PYLL *without* disability	-384 (-538; -221)	76%
	PYLL *with* disability	-124 (-227; -24)	
Hungary			
	Total PYLL	-593 (-764; -446)	
	PYLL *without* disability	-431 (-590; -288)	73%
	PYLL *with* disability	-162 (-234; -85)	
Italy			
	Total PYLL	-1,760 (-2,353; -1,219)	
	PYLL *without* disability	-929 (-1,687; -312)	53%
	PYLL *with* disability	-832 (-1,321; -284)	
Netherlands			
	Total PYLL	-371 (-581; -169)	
	PYLL *without* disability	-244 (-498; -23)	66%
	PYLL *with* disability	-127 (-282; 21)	
Poland			
	Total PYLL	-2,519 (-2,992; -2,054)	
	PYLL *without* disability	-1,651 (-2,162; -1,125)	66%
	PYLL *with* disability	-868 (-1,171; -546)	
Portugal			
	Total PYLL	-315 (-450; -149)	
	PYLL *without* disability	-206 (-366; -72)	65%
	PYLL *with* disability	-109 (-205; -6)	
Slovenia			
	Total PYLL	-99 (-128; -66)	
	PYLL *without* disability	-60 (-82; -37)	61%
	PYLL *with* disability	-38 (-60; -22)	
Spain			
	Total PYLL	-3,179 (-3,832; -2,456)	
	PYLL *without* disability	-1,552 (-2,166; -996)	49%
	PYLL *with* disability	-1,627 (-2,045; -1,084)	
Sweden			
	Total PYLL	-115 (-184; -47)	
	PYLL *without* disability	-69 (-220; 62)	60%
	PYLL *with* disability	-46 (-134; 49)	
Switzerland			
	Total PYLL	-140 (-188; -91)	
	PYLL *without* disability	-88 (-199; 19)	63%
	PYLL *with* disability	-53 (-122; 23)	
United Kingdom			
	Total PYLL	-2,299 (-2,879; -1,648)	
	PYLL *without* disability	-1,493 (-1,820; -1,132)	68%
	PYLL *with* disability	-806 (-1,086; -505)	

Figure G: Person-years of life lost in the population aged 35 and over, by age-group and sex, estimated by comparing Markov models with projected and observed all-cause mortality rates. To enable comparability between countries, the range of the Y-axis is proportional to the total population aged 35+ in each country. Panels are sorted by descending gross domestic product per capita.

Error bars represent 95% Uncertainty Intervals obtained from Monte-Carlo simulation.

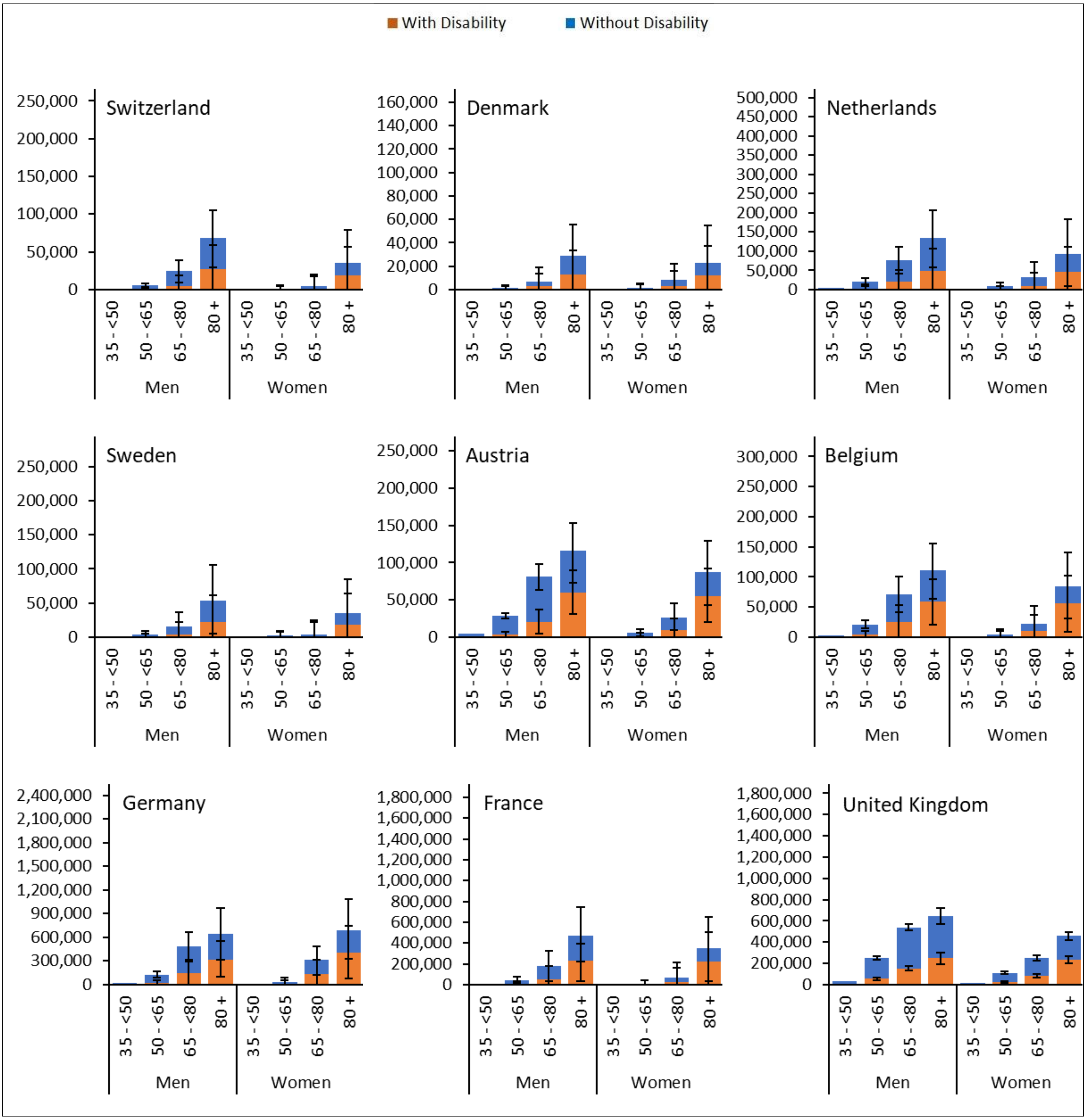

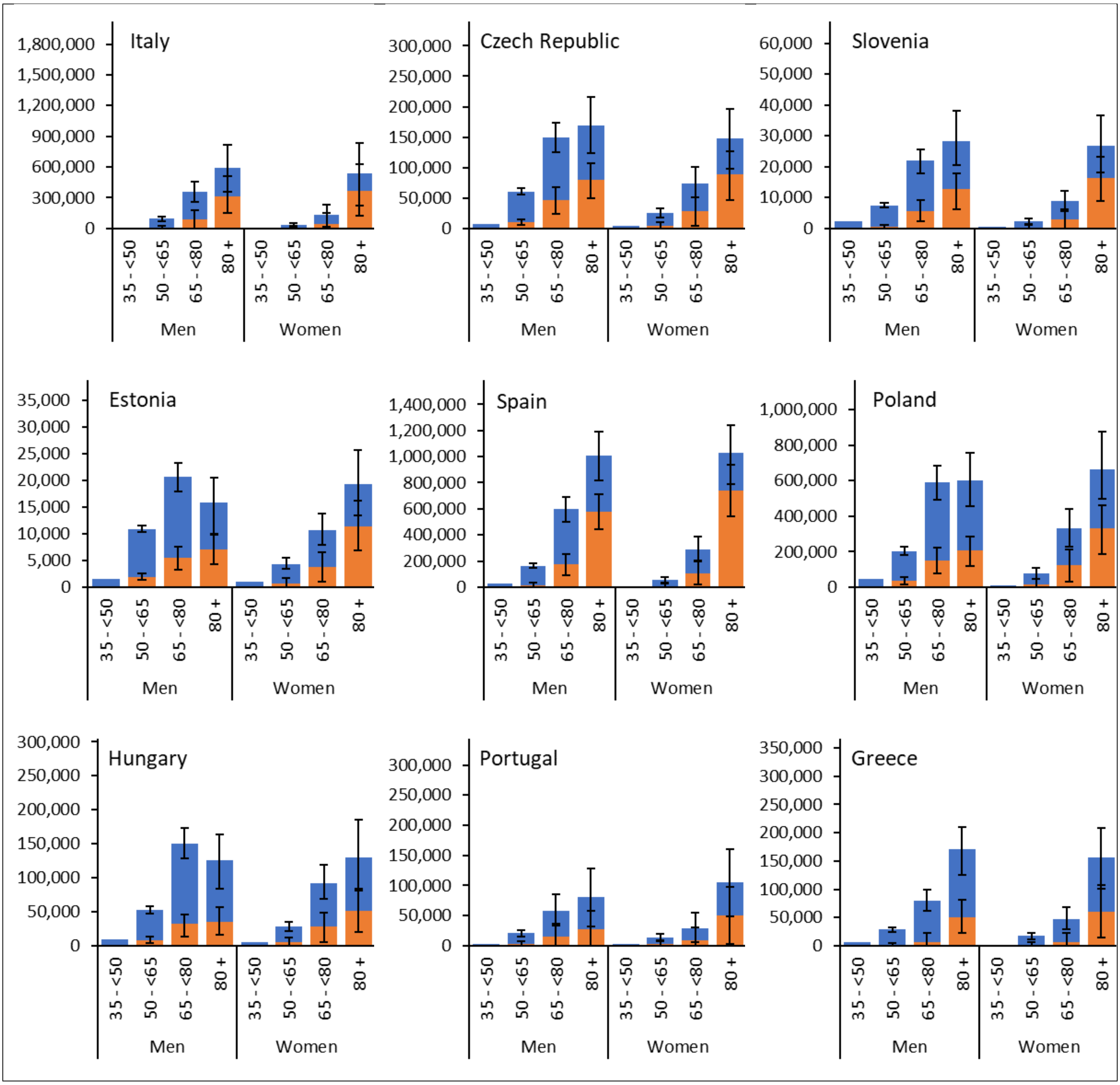
Italy
1,800,000
1,500,000
1,200,000
900,000
600,000
300,000
0
Czech Republic
300,000
250,000
200,000
150,000
100,000
50,000
0
Slovenia
60,000
50,000
40,000
30,000
20,000
10,000
0
Estonia
35,000
30,000
25,000
20,000
15,000
10,000
5,000
0
Spain
1,400,000
1,200,000
1,000,000
800,000
600,000
400,000
200,000
0
Poland
1,000,000
800,000
600,000
400,000
200,000
0
Hungary
300,000
250,000
200,000
150,000
100,000
50,000
0
Portugal
300,000
250,000
200,000
150,000
100,000
50,000
0
Greece
350,000
300,000
250,000
200,000
150,000
100,000
50,000
0
35 - <50
50 - <65
65 - <80
80 +
Men
Women

Figure H: Person-years of life lost per 1000 population over the years 2020-2022 by vaccination coverage and 2019 Gross Domestic Product per capita

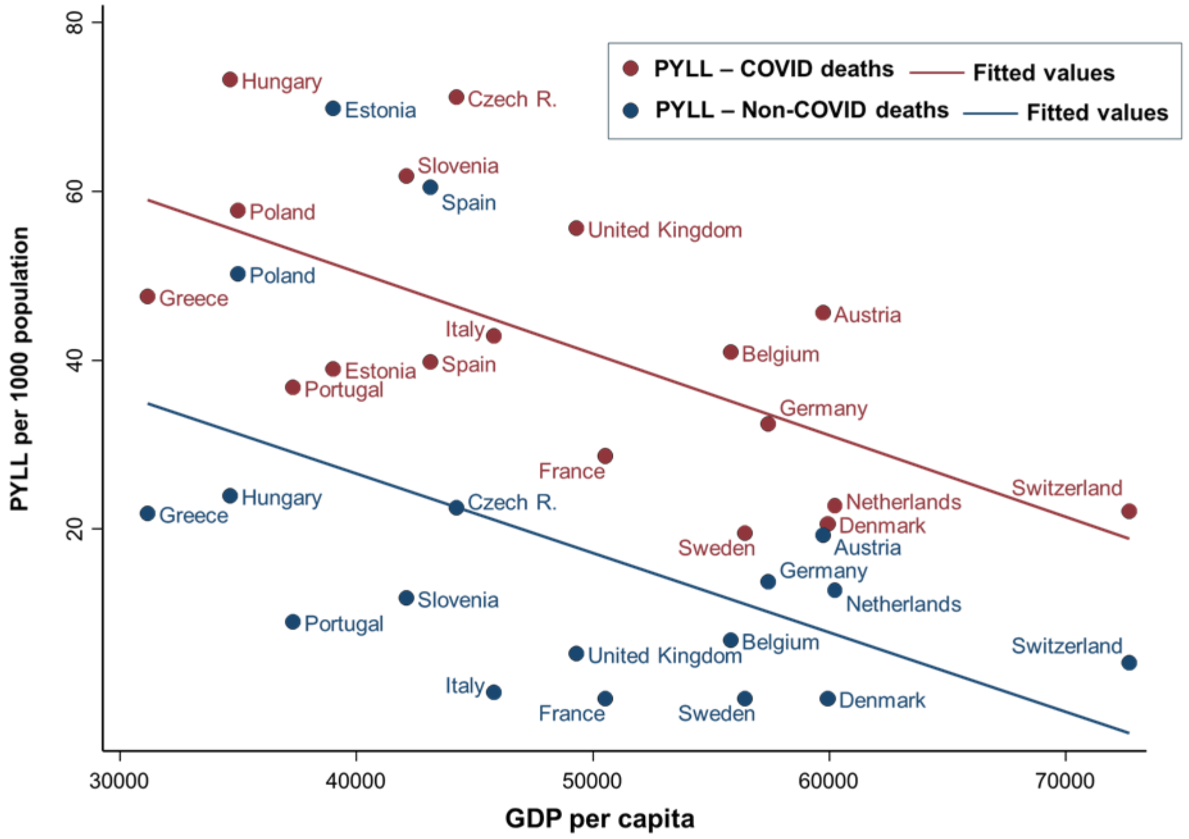

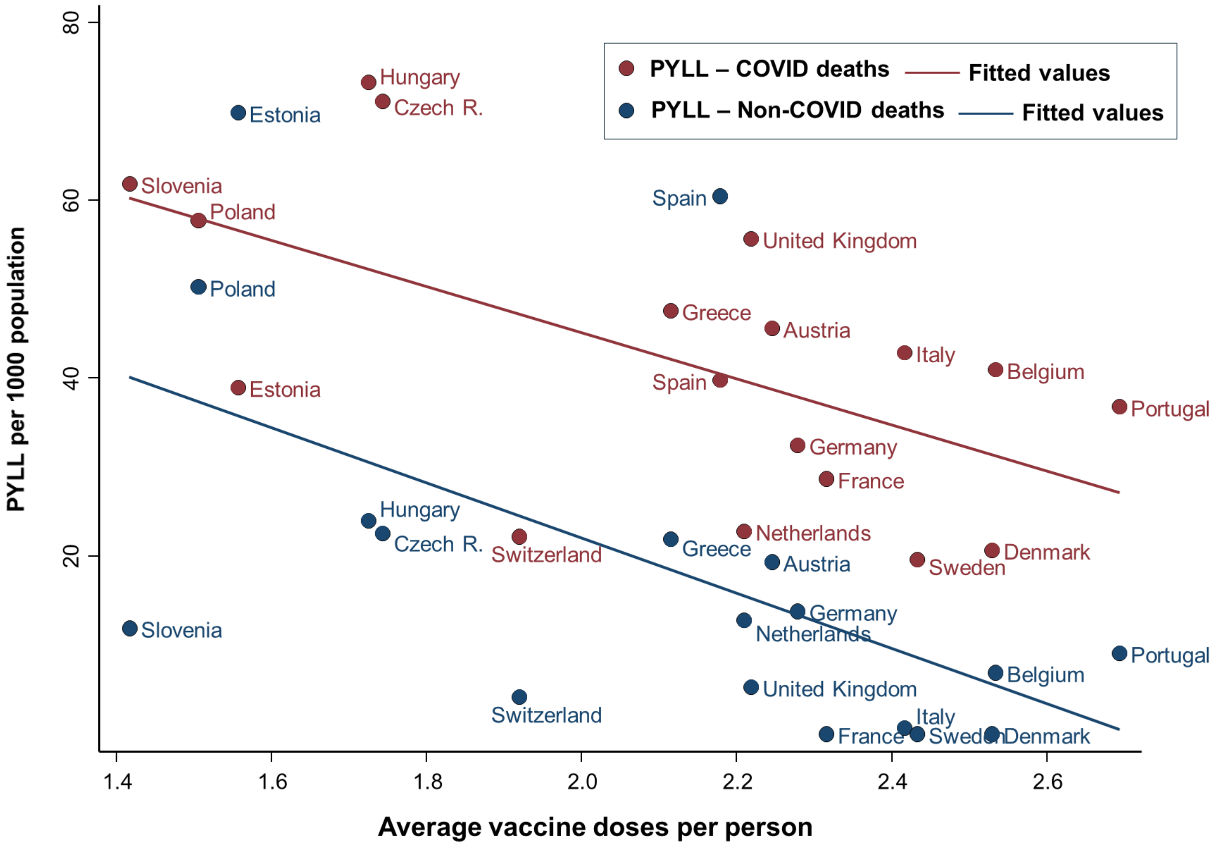

Figure I: Change in Life-Expectancy at 35 (LE-35) over time. Numbers represent average change in LE-35 per year in the decade leading to the pandemic (2009-2019).

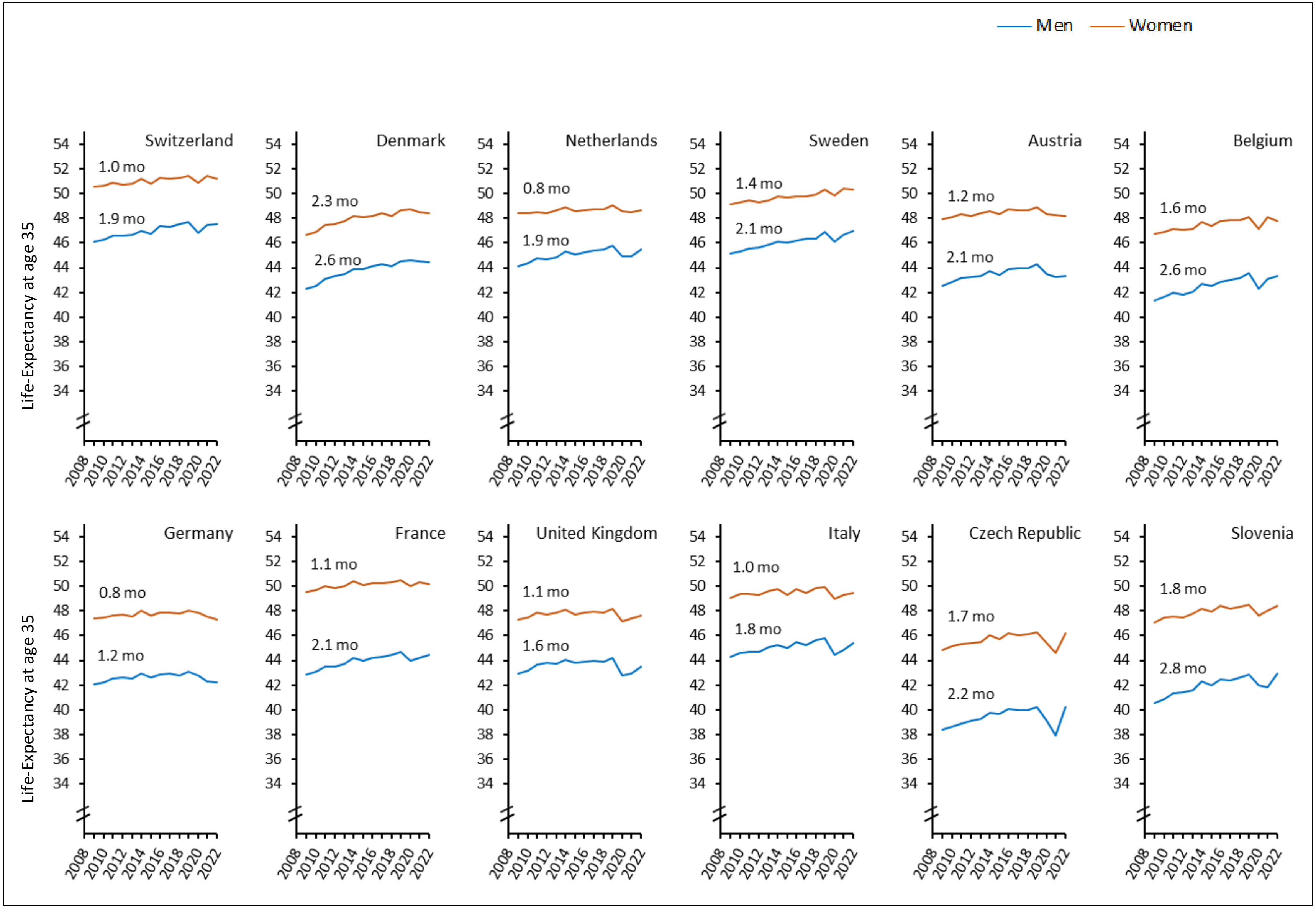

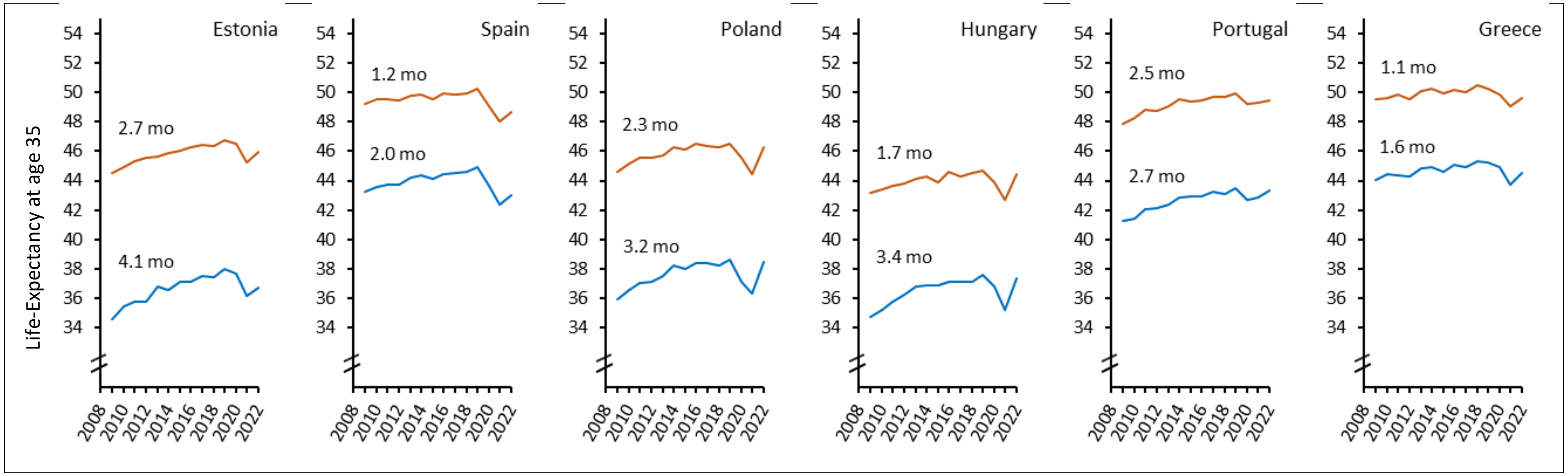

Life-Expectancy at age 35
Estonia
2.7 mo
4.1 mo
Spain
1.2 mo
2.0 mo
Poland
2.3 mo
3.2 mo
Hungary
1.7 mo
3.4 mo
Portugal
2.5 mo
2.7 mo
Greece
1.1 mo
1.6 mo

Table I: Life-expectancy at age 35 (LE) with and without disability by country and year. Numbers in parenthesis are 95% uncertainty intervals from Markov model Monte Carlo simulations.

	Men				Women			
	Observed	Expected	Difference explained by Covid deaths	Difference explained by non-Covid deaths	Observed	Expected	Difference explained by Covid deaths	Difference explained by non-Covid deaths
**Austria**								
2019								
Total	45.4 (44.9, 45.9)				49.9 (49.4, 50.3)			
With Disability	3.6 (3.2, 4.1)				5.5 (5.0, 6.2)			
2020								
Total	44.4 (44.0, 44.9)	45.5 (45.0, 46.1)	-0.9 (-1.4, -0.4)	-0.2 (-0.6, 0.3)	49.3 (48.8, 49.8)	50.0 (49.5, 50.4)	-0.7 (-1.2, -0.2)	0.1 (-0.4, 0.5)
With Disability	3.4 (3.0, 3.8)	3.7 (3.2, 4.2)	-0.2 (-0.7, 0.2)	-0.1 (-0.5, 0.4)	5.3 (4.7, 6.0)	5.6 (5.0, 6.3)	-0.3 (-0.9, 0.3)	0.0 (-0.6, 0.6)
2021								
Total	44.2 (43.8, 44.7)	45.7 (45.2, 46.2)	-1.1 (-1.6, -0.6)	-0.4 (-0.9, 0.1)	49.3 (48.8, 49.8)	50.0 (49.6, 50.5)	-1.1 (-1.6, -0.6)	0.3 (-0.2, 0.8)
With Disability	3.4 (3.0, 3.8)	3.8 (3.3, 4.3)	-0.3 (-0.7, 0.2)	-0.1 (-0.5, 0.4)	5.4 (4.7, 6.0)	5.7 (5.1, 6.4)	-0.4 (-1.0, 0.2)	0.1 (-0.5, 0.7)
2022								
Total	44.3 (43.8, 44.8)	45.9 (45.3, 46.4)	-0.5 (-1.0, 0.1)	-1.1 (-1.6, -0.5)	49.2 (48.7, 49.7)	50.1 (49.7, 50.6)	-0.5 (-1.0, 0.0)	-0.5 (-1.0, 0.0)
With Disability	3.4 (3.0, 3.9)	3.8 (3.3, 4.4)	-0.1 (-0.6, 0.3)	-0.3 (-0.7, 0.2)	5.3 (4.7, 6.0)	5.7 (5.1, 6.5)	-0.2 (-0.8, 0.4)	-0.2 (-0.8, 0.4)
**Belgium**								
2019								
Total	44.5 (44.1, 45.0)				49.0 (48.5, 49.5)			
With Disability	4.7 (4.2, 5.2)				6.7 (6.1, 7.4)			
2020								
Total	43.3 (42.8, 43.8)	44.7 (44.2, 45.1)	-1.8 (-2.2, -1.3)	0.4 (-0.1, 0.9)	48.1 (47.5, 48.6)	49.1 (48.6, 49.6)	-1.4 (-2.0, -0.9)	0.4 (-0.1, 1.0)
With Disability	4.3 (3.8, 4.8)	4.8 (4.3, 5.3)	-0.5 (-1.0, -0.1)	0.1 (-0.3, 0.5)	6.3 (5.7, 7.0)	6.8 (6.1, 7.5)	-0.6 (-1.2, 0.0)	0.1 (-0.5, 0.7)
2021								
Total	44.1 (43.6, 44.6)	44.9 (44.4, 45.3)	-0.8 (-1.3, -0.3)	0.0 (-0.5, 0.5)	49.1 (48.5, 49.6)	49.2 (48.7, 49.7)	-0.8 (-1.4, -0.3)	0.7 (0.1, 1.2)
With Disability	4.6 (4.1, 5.1)	4.9 (4.4, 5.4)	-0.3 (-0.7, 0.2)	0.0 (-0.4, 0.5)	6.8 (6.1, 7.5)	6.9 (6.2, 7.6)	-0.4 (-1.0, 0.2)	0.3 (-0.3, 0.9)
2022								
Total	44.3 (43.8, 44.8)	45.1 (44.6, 45.5)	-0.4 (-0.9, 0.1)	-0.4 (-0.9, 0.1)	48.8 (48.2, 49.3)	49.4 (48.9, 49.8)	-0.4 (-1.0, 0.1)	-0.2 (-0.8, 0.4)
With Disability	4.7 (4.2, 5.2)	5.0 (4.5, 5.5)	-0.2 (-0.6, 0.3)	-0.1 (-0.6, 0.4)	6.6 (6.0, 7.4)	7.0 (6.3, 7.7)	-0.2 (-0.9, 0.4)	-0.1 (-0.7, 0.6)
**Czech Republic**								
2019								

Total	41.4 (40.9, 41.8)				47.3 (46.8, 47.8)			
With Disability	3.4 (3.1, 3.8)				5.5 (4.9, 6.1)			
2020								
Total	40.1 (39.7, 40.6)	41.5 (41.0, 41.9)	-0.9 (-1.3, -0.4)	-0.6 (-1.0, -0.1)	46.4 (46.0, 46.9)	47.4 (46.9, 47.9)	-0.8 (-1.2, -0.3)	-0.3 (-0.7, 0.2)
With Disability	3.1 (2.8, 3.5)	3.5 (3.1, 3.8)	-0.2 (-0.5, 0.1)	-0.2 (-0.5, 0.2)	5.1 (4.6, 5.8)	5.5 (5.0, 6.2)	-0.3 (-0.8, 0.3)	-0.1 (-0.7, 0.4)
2021								
Total	38.9 (38.4, 39.4)	41.7 (41.2, 42.1)	-1.8 (-2.2, -1.3)	-1.0 (-1.5, -0.6)	45.6 (45.1, 46.1)	47.5 (47.0, 48.0)	-1.9 (-2.4, -1.5)	0.0 (-0.5, 0.5)
With Disability	2.9 (2.6, 3.2)	3.5 (3.2, 3.9)	-0.4 (-0.7, -0.1)	-0.2 (-0.6, 0.1)	5.0 (4.4, 5.6)	5.6 (5.1, 6.3)	-0.7 (-1.2, -0.1)	0.0 (-0.5, 0.6)
2022								
Total	41.2 (40.8, 41.7)	41.9 (41.3, 42.3)	-0.3 (-0.8, 0.2)	-0.3 (-0.8, 0.2)	47.2 (46.7, 47.7)	47.6 (47.1, 48.2)	-0.4 (-0.9, 0.1)	-0.1 (-0.6, 0.5)
With Disability	3.4 (3.1, 3.8)	3.6 (3.2, 4.0)	-0.1 (-0.5, 0.2)	-0.1 (-0.4, 0.2)	5.5 (4.9, 6.2)	5.7 (5.2, 6.4)	-0.2 (-0.8, 0.4)	0.0 (-0.6, 0.5)
**Denmark**								
2019								
Total	45.6 (45.1, 46.2)				49.6 (49.1, 50.3)			
With Disability	3.4 (2.9, 3.9)				5.0 (4.4, 5.7)			
2020								
Total	45.6 (45.2, 46.2)	45.8 (45.3, 46.4)	-0.3 (-0.8, 0.2)	0.1 (-0.3, 0.6)	49.7 (49.2, 50.3)	49.8 (49.2, 50.4)	-0.2 (-0.8, 0.4)	0.2 (-0.4, 0.7)
With Disability	3.5 (3.0, 4.0)	3.5 (3.0, 3.9)	0.0 (-0.5, 0.4)	0.0 (-0.4, 0.5)	5.1 (4.4, 5.8)	5.1 (4.5, 5.8)	-0.1 (-0.7, 0.6)	0.1 (-0.6, 0.7)
2021								
Total	45.5 (45.0, 46.1)	45.9 (45.4, 46.6)	-0.4 (-0.9, 0.1)	0.0 (-0.5, 0.5)	49.5 (48.9, 50.1)	49.9 (49.3, 50.6)	-0.3 (-0.9, 0.2)	-0.1 (-0.6, 0.5)
With Disability	3.5 (3.0, 4.0)	3.6 (3.1, 4.0)	-0.1 (-0.6, 0.4)	0.0 (-0.5, 0.4)	5.1 (4.4, 5.7)	5.2 (4.5, 5.9)	-0.1 (-0.8, 0.5)	0.0 (-0.7, 0.6)
2022								
Total	45.4 (44.9, 46.0)	46.1 (45.6, 46.8)	-0.7 (-1.2, -0.2)	0.0 (-0.5, 0.5)	49.4 (48.8, 50.0)	50.1 (49.4, 50.7)	-0.7 (-1.4, -0.1)	0.1 (-0.6, 0.7)
With Disability	3.5 (3.0, 4.0)	3.7 (3.1, 4.1)	-0.2 (-0.7, 0.3)	0.0 (-0.5, 0.5)	5.0 (4.4, 5.7)	5.3 (4.6, 6.0)	-0.3 (-0.9, 0.4)	0.0 (-0.6, 0.7)
**Estonia**								
2019								
Total	39.1 (38.7, 39.6)				47.8 (47.4, 48.3)			
With Disability	3.2 (2.9, 3.5)				6.1 (5.5, 6.7)			
2020								
Total	38.7 (38.2, 39.2)	39.4 (38.9, 39.9)	-0.1 (-0.6, 0.4)	-0.6 (-1.1, -0.1)	47.5 (47.0, 47.9)	47.9 (47.5, 48.4)	-0.1 (-0.6, 0.3)	-0.3 (-0.8, 0.1)
With Disability	3.2 (2.9, 3.4)	3.3 (3.0, 3.6)	0.0 (-0.3, 0.3)	-0.1 (-0.4, 0.2)	6.0 (5.4, 6.6)	6.1 (5.6, 6.8)	0.0 (-0.6, 0.5)	-0.1 (-0.7, 0.5)
2021								
Total	37.2 (36.7, 37.7)	39.7 (39.2, 40.2)	-1.0 (-1.5, -0.4)	-1.5 (-2.1, -1.0)	46.2 (45.8, 46.7)	48.1 (47.6, 48.7)	-1.3 (-1.8, -0.8)	-0.6 (-1.1, -0.1)
With Disability	2.8 (2.6, 3.1)	3.4 (3.1, 3.7)	-0.2 (-0.5, 0.0)	-0.3 (-0.6, 0.0)	5.6 (5.0, 6.1)	6.3 (5.7, 6.9)	-0.5 (-1.0, 0.1)	-0.2 (-0.8, 0.4)
2022								
Total	37.8 (37.2, 38.3)	40.0 (39.4, 40.5)	-0.4 (-1.0, 0.2)	-1.8 (-2.4, -1.2)	46.9 (46.4, 47.4)	48.3 (47.8, 48.9)	-0.6 (-1.2, -0.1)	-0.8 (-1.3, -0.2)

With Disability	3.0 (2.7, 3.3)	3.5 (3.2, 3.8)	-0.1 (-0.4, 0.2)	-0.4 (-0.7, -0.1)	5.9 (5.3, 6.5)	6.4 (5.8, 7.0)	-0.3 (-0.9, 0.3)	-0.2 (-0.8, 0.4)
**France**								
2019								
Total	45.6 (45.1, 46.1)				51.5 (51.0, 52.0)			
With Disability	3.9 (3.5, 4.3)				6.4 (5.6, 7.1)			
2020								
Total	45.0 (44.4, 45.4)	45.7 (45.2, 46.2)	-1.1 (-1.7, -0.6)	0.3 (-0.2, 0.9)	51.0 (50.5, 51.4)	51.6 (51.1, 52.1)	-1.0 (-1.5, -0.5)	0.5 (0.0, 1.0)
With Disability	3.7 (3.3, 4.2)	4.0 (3.5, 4.4)	-0.3 (-0.7, 0.1)	0.1 (-0.3, 0.5)	6.1 (5.4, 6.8)	6.4 (5.7, 7.2)	-0.5 (-1.2, 0.2)	0.2 (-0.5, 0.9)
2021								
Total	45.2 (44.7, 45.7)	45.9 (45.3, 46.4)	-1.0 (-1.6, -0.5)	0.4 (-0.2, 0.9)	51.3 (50.8, 51.7)	51.6 (51.1, 52.1)	-1.2 (-1.7, -0.7)	0.8 (0.3, 1.4)
With Disability	3.8 (3.4, 4.3)	4.0 (3.6, 4.5)	-0.3 (-0.7, 0.1)	0.1 (-0.4, 0.5)	6.3 (5.5, 7.0)	6.5 (5.7, 7.2)	-0.5 (-1.2, 0.2)	0.3 (-0.4, 1.0)
2022								
Total	45.4 (44.9, 45.9)	46.0 (45.4, 46.5)	-0.5 (-1.1, 0.0)	-0.1 (-0.6, 0.5)	51.2 (50.6, 51.6)	51.7 (51.2, 52.2)	-0.7 (-1.2, -0.2)	0.2 (-0.3, 0.7)
With Disability	3.9 (3.4, 4.4)	4.1 (3.6, 4.6)	-0.2 (-0.6, 0.2)	0.0 (-0.5, 0.4)	6.2 (5.5, 7.0)	6.6 (5.8, 7.3)	-0.4 (-1.1, 0.4)	0.0 (-0.7, 0.8)
**Germany**								
2019								
Total	44.1 (43.6, 44.5)				49.1 (48.6, 49.6)			
With Disability	3.9 (3.5, 4.4)				5.9 (5.2, 6.6)			
2020								
Total	43.8 (43.3, 44.3)	44.1 (43.6, 44.6)	-0.4 (-0.9, 0.1)	0.0 (-0.5, 0.5)	48.9 (48.4, 49.3)	49.2 (48.6, 49.7)	-0.3 (-0.9, 0.2)	0.0 (-0.5, 0.6)
With Disability	3.8 (3.4, 4.3)	4.0 (3.6, 4.4)	-0.1 (-0.5, 0.3)	0.0 (-0.4, 0.4)	5.8 (5.2, 6.4)	5.9 (5.3, 6.7)	-0.1 (-0.7, 0.5)	0.0 (-0.6, 0.6)
2021								
Total	43.3 (42.8, 43.8)	44.2 (43.7, 44.8)	-0.9 (-1.4, -0.4)	0.0 (-0.6, 0.5)	48.6 (48.0, 49.1)	49.3 (48.7, 49.8)	-1.0 (-1.5, -0.4)	0.3 (-0.3, 0.8)
With Disability	3.8 (3.4, 4.2)	4.0 (3.6, 4.5)	-0.3 (-0.6, 0.1)	0.0 (-0.4, 0.4)	5.8 (5.1, 6.4)	6.0 (5.4, 6.8)	-0.3 (-1.0, 0.3)	0.1 (-0.5, 0.8)
2022								
Total	43.2 (42.7, 43.8)	44.3 (43.7, 44.9)	-0.5 (-1.0, 0.1)	-0.6 (-1.1, -0.1)	48.3 (47.8, 48.8)	49.4 (48.8, 49.9)	-0.6 (-1.1, 0.0)	-0.5 (-1.0, 0.0)
With Disability	3.8 (3.4, 4.2)	4.1 (3.7, 4.6)	-0.2 (-0.6, 0.2)	-0.2 (-0.5, 0.2)	5.7 (5.0, 6.3)	6.1 (5.4, 6.9)	-0.2 (-0.9, 0.5)	-0.2 (-0.9, 0.5)
**Greece**								
2019								
Total	46.5 (45.9, 47.0)				51.5 (50.9, 52.0)			
With Disability	2.5 (2.2, 3.0)				4.0 (3.5, 4.8)			
2020								
Total	45.9 (45.4, 46.5)	46.6 (46.0, 47.1)	-0.6 (-1.2, 0.0)	-0.1 (-0.7, 0.5)	50.9 (50.3, 51.5)	51.5 (51.0, 52.0)	-0.4 (-1.0, 0.2)	-0.3 (-0.8, 0.3)
With Disability	2.4 (2.0, 2.9)	2.6 (2.2, 3.0)	-0.1 (-0.5, 0.3)	0.0 (-0.4, 0.3)	3.9 (3.1, 4.5)	4.1 (3.5, 4.8)	-0.1 (-0.8, 0.5)	-0.1 (-0.8, 0.5)
2021								
Total	44.7 (44.2, 45.3)	46.7 (46.0, 47.2)	-1.7 (-2.3, -1.1)	-0.3 (-0.9, 0.3)	50.1 (49.5, 50.7)	51.6 (51.0, 52.2)	-1.6 (-2.2, -1.0)	0.1 (-0.5, 0.7)

With Disability	2.3 (1.9, 2.7)	2.6 (2.2, 3.1)	-0.3 (-0.7, 0.0)	0.0 (-0.4, 0.3)	3.7 (3.0, 4.4)	4.1 (3.6, 4.9)	-0.4 (-1.1, 0.2)	0.0 (-0.6, 0.7)
2022								
Total	45.5 (45.0, 46.1)	46.8 (46.2, 47.4)	-1.3 (-1.9, -0.7)	0.0 (-0.6, 0.6)	50.6 (50.0, 51.2)	51.7 (51.1, 52.3)	-1.3 (-1.9, -0.7)	0.1 (-0.4, 0.7)
With Disability	2.4 (2.0, 2.9)	2.7 (2.3, 3.2)	-0.3 (-0.7, 0.1)	0.0 (-0.4, 0.4)	3.8 (3.0, 4.5)	4.2 (3.6, 5.0)	-0.4 (-1.1, 0.3)	0.0 (-0.7, 0.7)
**Hungary**								
2019								
Total	38.7 (38.1, 39.2)				45.6 (45.1, 46.2)			
With Disability	2.5 (2.2, 2.8)				4.2 (3.7, 4.8)			
2020								
Total	37.8 (37.2, 38.3)	38.8 (38.2, 39.4)	-0.6 (-1.1, 0.0)	-0.5 (-1.0, 0.1)	44.9 (44.3, 45.5)	45.7 (45.1, 46.3)	-0.6 (-1.2, 0.0)	-0.2 (-0.8, 0.3)
With Disability	2.3 (2.0, 2.6)	2.5 (2.2, 2.8)	-0.2 (-0.4, 0.1)	-0.1 (-0.3, 0.2)	4.0 (3.5, 4.5)	4.3 (3.8, 4.8)	-0.2 (-0.7, 0.3)	0.0 (-0.5, 0.4)
2021								
Total	36.3 (35.7, 36.8)	39.0 (38.4, 39.6)	-1.8 (-2.4, -1.2)	-0.9 (-1.5, -0.3)	43.7 (43.0, 44.3)	45.8 (45.2, 46.4)	-2.2 (-2.8, -1.6)	0.0 (-0.6, 0.6)
With Disability	2.1 (1.9, 2.4)	2.6 (2.3, 2.9)	-0.4 (-0.6, -0.1)	-0.1 (-0.4, 0.2)	3.8 (3.3, 4.3)	4.3 (3.8, 4.9)	-0.6 (-1.1, -0.1)	0.1 (-0.4, 0.6)
2022								
Total	38.4 (37.8, 38.9)	39.2 (38.5, 39.8)	-0.4 (-1.0, 0.3)	-0.4 (-1.1, 0.2)	45.5 (44.8, 46.2)	45.9 (45.3, 46.6)	-0.6 (-1.2, 0.0)	0.1 (-0.5, 0.7)
With Disability	2.4 (2.2, 2.7)	2.7 (2.4, 3.0)	-0.2 (-0.4, 0.1)	-0.1 (-0.4, 0.2)	4.2 (3.7, 4.7)	4.4 (3.9, 5.0)	-0.2 (-0.7, 0.3)	0.0 (-0.5, 0.5)
**Italy**								
2019								
Total	46.7 (46.2, 47.1)				50.8 (50.4, 51.2)			
With Disability	3.9 (3.4, 4.3)				5.9 (5.3, 6.5)			
2020								
Total	45.4 (45.0, 45.9)	46.7 (46.3, 47.2)	-1.3 (-1.7, -0.9)	0.0 (-0.4, 0.5)	50.0 (49.6, 50.4)	50.8 (50.4, 51.3)	-1.1 (-1.5, -0.7)	0.2 (-0.2, 0.7)
With Disability	3.5 (3.1, 3.9)	3.9 (3.4, 4.4)	-0.4 (-0.9, 0.0)	0.0 (-0.4, 0.4)	5.5 (4.9, 6.1)	5.9 (5.3, 6.6)	-0.5 (-1.1, 0.1)	0.1 (-0.5, 0.6)
2021								
Total	45.9 (45.5, 46.3)	46.8 (46.4, 47.3)	-1.1 (-1.5, -0.7)	0.1 (-0.3, 0.6)	50.3 (49.8, 50.7)	50.9 (50.5, 51.3)	-1.1 (-1.6, -0.7)	0.5 (0.1, 1.0)
With Disability	3.6 (3.3, 4.1)	4.0 (3.5, 4.5)	-0.4 (-0.8, 0.0)	0.1 (-0.4, 0.5)	5.7 (5.1, 6.3)	6.0 (5.4, 6.7)	-0.5 (-1.1, 0.1)	0.2 (-0.4, 0.8)
2022								
Total	46.4 (46.0, 46.9)	47.0 (46.5, 47.5)	-0.7 (-1.1, -0.2)	0.1 (-0.3, 0.6)	50.5 (50.0, 50.9)	51.0 (50.5, 51.4)	-0.8 (-1.2, -0.3)	0.3 (-0.1, 0.7)
With Disability	3.8 (3.4, 4.3)	4.1 (3.6, 4.6)	-0.3 (-0.8, 0.1)	0.1 (-0.4, 0.5)	5.7 (5.1, 6.4)	6.0 (5.4, 6.8)	-0.4 (-1.0, 0.2)	0.1 (-0.5, 0.7)
**Netherlands**								
2019								
Total	46.7 (46.2, 47.2)				50.0 (49.4, 50.6)			
With Disability	3.4 (2.9, 3.9)				4.8 (4.2, 5.6)			
2020								
Total	45.9 (45.4, 46.4)	46.8 (46.3, 47.3)	-0.7 (-1.2, -0.3)	-0.1 (-0.6, 0.3)	49.5 (49.0, 50.1)	50.0 (49.4, 50.6)	-0.6 (-1.2, 0.0)	0.1 (-0.5, 0.7)

With Disability	3.2 (2.7, 3.7)	3.5 (3.0, 4.0)	-0.2 (-0.7, 0.3)	-0.1 (-0.5, 0.4)	4.7 (4.0, 5.3)	4.9 (4.3, 5.6)	-0.2 (-0.8, 0.4)	0.0 (-0.6, 0.6)
2021								
Total	45.9 (45.5, 46.4)	46.9 (46.4, 47.4)	-0.6 (-1.1, -0.1)	-0.4 (-0.8, 0.1)	49.5 (48.9, 50.1)	50.1 (49.4, 50.7)	-0.6 (-1.2, 0.0)	0.0 (-0.7, 0.6)
With Disability	3.3 (2.8, 3.8)	3.5 (3.0, 4.0)	-0.2 (-0.7, 0.3)	-0.1 (-0.6, 0.4)	4.7 (4.1, 5.4)	4.9 (4.3, 5.7)	-0.2 (-0.8, 0.4)	0.0 (-0.6, 0.6)
2022								
Total	46.5 (46.0, 47.0)	47.0 (46.5, 47.5)	-0.1 (-0.6, 0.5)	-0.5 (-1.0, 0.0)	49.7 (49.1, 50.2)	50.2 (49.5, 50.8)	-0.1 (-0.7, 0.6)	-0.4 (-1.1, 0.2)
With Disability	3.5 (3.0, 4.0)	3.6 (3.1, 4.1)	-0.1 (-0.6, 0.5)	-0.1 (-0.6, 0.4)	4.8 (4.1, 5.5)	5.0 (4.4, 5.7)	0.0 (-0.7, 0.6)	-0.2 (-0.8, 0.5)
**Poland**								
2019								
Total	39.7 (39.2, 40.2)				47.6 (47.0, 48.1)			
With Disability	3.0 (2.6, 3.3)				5.2 (4.6, 5.9)			
2020								
Total	38.1 (37.6, 38.6)	39.9 (39.3, 40.4)	-0.6 (-1.1, 0.0)	-1.2 (-1.7, -0.6)	46.5 (46.0, 47.1)	47.7 (47.1, 48.2)	-0.6 (-1.1, 0.0)	-0.6 (-1.2, 0.0)
With Disability	2.6 (2.4, 3.0)	3.0 (2.7, 3.3)	-0.1 (-0.4, 0.2)	-0.3 (-0.6, 0.0)	4.8 (4.2, 5.5)	5.3 (4.7, 5.9)	-0.2 (-0.8, 0.3)	-0.2 (-0.8, 0.4)
2021								
Total	37.3 (36.8, 37.8)	40.0 (39.4, 40.6)	-1.4 (-1.9, -0.8)	-1.4 (-2.0, -0.8)	45.5 (44.8, 46.0)	47.8 (47.2, 48.4)	-1.6 (-2.2, -1.0)	-0.7 (-1.3, -0.1)
With Disability	2.5 (2.3, 2.8)	3.1 (2.7, 3.4)	-0.3 (-0.6, 0.0)	-0.3 (-0.6, 0.0)	4.5 (4.0, 5.2)	5.3 (4.7, 6.0)	-0.6 (-1.1, 0.0)	-0.2 (-0.8, 0.4)
2022								
Total	39.5 (38.9, 40.0)	40.2 (39.6, 40.8)	-0.3 (-0.9, 0.3)	-0.4 (-1.0, 0.2)	47.3 (46.6, 47.8)	47.9 (47.3, 48.5)	-0.4 (-1.0, 0.2)	-0.2 (-0.9, 0.4)
With Disability	2.9 (2.6, 3.3)	3.1 (2.8, 3.5)	-0.1 (-0.4, 0.2)	-0.1 (-0.4, 0.2)	5.1 (4.5, 5.8)	5.4 (4.8, 6.1)	-0.2 (-0.8, 0.4)	-0.1 (-0.7, 0.5)
**Portugal**								
2019								
Total	44.6 (44.0, 45.0)				51.0 (50.2, 51.6)			
With Disability	3.6 (3.2, 4.1)				5.9 (5.1, 6.7)			
2020								
Total	43.7 (43.1, 44.3)	44.7 (44.1, 45.1)	-0.7 (-1.3, -0.1)	-0.2 (-0.8, 0.4)	50.2 (49.7, 50.8)	51.1 (50.3, 51.7)	-0.7 (-1.3, 0.0)	-0.2 (-0.8, 0.5)
With Disability	3.5 (3.0, 4.0)	3.7 (3.3, 4.1)	-0.1 (-0.6, 0.3)	0.0 (-0.5, 0.4)	5.6 (4.9, 6.4)	5.9 (5.2, 6.8)	-0.2 (-0.9, 0.6)	-0.1 (-0.8, 0.7)
2021								
Total	43.8 (43.2, 44.5)	44.8 (44.2, 45.4)	-1.2 (-1.8, -0.6)	0.2 (-0.4, 0.9)	50.3 (49.8, 50.9)	51.2 (50.4, 51.8)	-1.4 (-2.1, -0.8)	0.5 (-0.1, 1.2)
With Disability	3.5 (3.1, 4.1)	3.8 (3.3, 4.2)	-0.3 (-0.7, 0.2)	0.0 (-0.4, 0.5)	5.7 (4.9, 6.4)	6.0 (5.2, 6.9)	-0.4 (-1.2, 0.3)	0.1 (-0.6, 0.9)
2022								
Total	44.3 (43.7, 45.0)	45.0 (44.4, 45.6)	-0.6 (-1.3, 0.1)	-0.1 (-0.8, 0.6)	50.4 (49.9, 51.1)	51.4 (50.6, 52.0)	-0.8 (-1.4, -0.1)	-0.2 (-0.9, 0.5)
With Disability	3.7 (3.2, 4.2)	3.8 (3.4, 4.3)	-0.1 (-0.6, 0.3)	0.0 (-0.5, 0.5)	5.7 (5.0, 6.5)	6.1 (5.3, 7.0)	-0.2 (-1.0, 0.5)	-0.1 (-0.8, 0.7)
**Slovenia**								
2019								
Total	44.1 (43.7, 44.5)				49.7 (49.3, 50.1)			

With Disability	3.4 (3.1, 3.8)				5.5 (5.0, 6.0)			
2020								
Total	43.0 (42.6, 43.4)	44.3 (43.8, 44.7)	-1.2 (-1.6, -0.8)	-0.1 (-0.5, 0.3)	48.6 (48.2, 49.0)	49.8 (49.4, 50.2)	-1.1 (-1.5, -0.7)	-0.1 (-0.5, 0.3)
With Disability	3.0 (2.7, 3.4)	3.5 (3.1, 3.8)	-0.3 (-0.7, 0.0)	-0.1 (-0.4, 0.3)	5.0 (4.5, 5.6)	5.6 (5.0, 6.1)	-0.4 (-0.9, 0.1)	-0.1 (-0.6, 0.4)
2021								
Total	42.8 (42.4, 43.3)	44.5 (44.0, 45.0)	-1.3 (-1.8, -0.8)	-0.4 (-0.8, 0.1)	49.0 (48.5, 49.4)	50.0 (49.5, 50.4)	-1.4 (-1.9, -1.0)	0.5 (0.0, 0.9)
With Disability	3.1 (2.8, 3.4)	3.5 (3.2, 3.9)	-0.4 (-0.7, 0.0)	-0.1 (-0.4, 0.3)	5.3 (4.7, 5.8)	5.6 (5.1, 6.2)	-0.5 (-1.1, 0.0)	0.2 (-0.4, 0.7)
2022								
Total	43.9 (43.4, 44.5)	44.7 (44.2, 45.2)	-0.5 (-1.0, 0.0)	-0.3 (-0.8, 0.2)	49.4 (48.9, 49.8)	50.1 (49.6, 50.5)	-0.7 (-1.1, -0.2)	-0.1 (-0.5, 0.4)
With Disability	3.4 (3.0, 3.7)	3.6 (3.3, 4.1)	-0.2 (-0.5, 0.2)	-0.1 (-0.4, 0.3)	5.4 (4.8, 6.0)	5.7 (5.2, 6.3)	-0.3 (-0.8, 0.3)	0.0 (-0.6, 0.5)
**Spain**								
2019								
Total	45.9 (45.4, 46.4)				51.2 (50.7, 51.6)			
With Disability	4.1 (3.6, 4.6)				6.7 (6.1, 7.5)			
2020								
Total	44.7 (44.2, 45.1)	45.9 (45.5, 46.4)	-1.2 (-1.7, -0.7)	0.0 (-0.5, 0.4)	50.1 (49.6, 50.5)	51.2 (50.8, 51.6)	-1.1 (-1.5, -0.7)	-0.1 (-0.5, 0.4)
With Disability	3.7 (3.3, 4.1)	4.2 (3.7, 4.6)	-0.4 (-0.9, 0.0)	0.0 (-0.5, 0.4)	6.2 (5.5, 6.9)	6.8 (6.2, 7.6)	-0.5 (-1.1, 0.1)	-0.1 (-0.7, 0.6)
2021								
Total	43.4 (42.8, 43.8)	46.1 (45.6, 46.6)	-0.9 (-1.4, -0.4)	-1.8 (-2.3, -1.3)	49.0 (48.6, 49.5)	51.3 (50.9, 51.8)	-1.0 (-1.5, -0.6)	-1.3 (-1.7, -0.8)
With Disability	3.3 (2.9, 3.7)	4.2 (3.7, 4.7)	-0.3 (-0.8, 0.1)	-0.6 (-1.1, -0.2)	5.7 (5.1, 6.4)	6.9 (6.3, 7.7)	-0.5 (-1.1, 0.2)	-0.7 (-1.3, 0.0)
2022								
Total	44.0 (43.4, 44.4)	46.2 (45.7, 46.8)	-0.5 (-1.1, 0.0)	-1.7 (-2.2, -1.1)	49.6 (49.2, 50.1)	51.5 (51.0, 51.9)	-0.7 (-1.2, -0.2)	-1.1 (-1.6, -0.6)
With Disability	3.5 (3.1, 3.9)	4.3 (3.8, 4.8)	-0.3 (-0.7, 0.2)	-0.6 (-1.0, -0.1)	6.0 (5.3, 6.7)	7.0 (6.3, 7.8)	-0.4 (-1.0, 0.3)	-0.6 (-1.3, 0.1)
**Sweden**								
2019								
Total	47.8 (47.3, 48.3)				51.3 (50.8, 51.9)			
With Disability	3.6 (3.1, 4.2)				5.0 (4.4, 5.7)			
2020								
Total	47.1 (46.6, 47.7)	47.9 (47.4, 48.5)	-1.0 (-1.6, -0.5)	0.2 (-0.3, 0.7)	50.8 (50.3, 51.4)	51.4 (50.9, 52.0)	-0.8 (-1.3, -0.2)	0.2 (-0.3, 0.7)
With Disability	3.4 (2.9, 3.9)	3.6 (3.2, 4.3)	-0.2 (-0.7, 0.3)	0.0 (-0.5, 0.5)	4.8 (4.1, 5.6)	5.1 (4.4, 5.8)	-0.3 (-1.0, 0.4)	0.0 (-0.7, 0.7)
2021								
Total	47.7 (47.2, 48.3)	48.1 (47.5, 48.6)	-0.8 (-1.3, -0.2)	0.4 (-0.2, 0.9)	51.4 (50.8, 52.0)	51.5 (51.0, 52.1)	-0.7 (-1.3, -0.2)	0.6 (0.1, 1.1)
With Disability	3.6 (3.1, 4.2)	3.7 (3.2, 4.4)	-0.2 (-0.7, 0.3)	0.1 (-0.4, 0.6)	5.1 (4.3, 5.8)	5.2 (4.5, 5.8)	-0.3 (-1.0, 0.4)	0.2 (-0.5, 0.9)
2022								
Total	48.0 (47.5, 48.6)	48.3 (47.7, 48.8)	-0.7 (-1.2, -0.1)	0.4 (-0.2, 0.9)	51.3 (50.8, 52.0)	51.6 (51.1, 52.3)	-0.7 (-1.2, -0.1)	0.4 (-0.2, 0.9)
With Disability	3.7 (3.2, 4.3)	3.8 (3.3, 4.5)	-0.2 (-0.7, 0.3)	0.1 (-0.4, 0.6)	5.1 (4.4, 5.9)	5.3 (4.6, 6.0)	-0.3 (-1.0, 0.4)	0.1 (-0.6, 0.9)

**Switzerland**								
2019								
Total	48.8 (48.2, 49.3)				52.5 (51.9, 53.0)			
With Disability	3.4 (3.0, 4.0)				4.9 (4.2, 5.6)			
2020								
Total	47.8 (47.3, 48.4)	48.9 (48.3, 49.4)	-1.2 (-1.7, -0.6)	0.1 (-0.4, 0.6)	51.9 (51.3, 52.5)	52.5 (52.0, 53.1)	-0.9 (-1.5, -0.4)	0.2 (-0.3, 0.8)
With Disability	3.2 (2.7, 3.7)	3.5 (3.0, 4.0)	-0.3 (-0.7, 0.2)	0.0 (-0.5, 0.4)	4.7 (4.0, 5.4)	5.0 (4.3, 5.7)	-0.3 (-0.9, 0.4)	0.0 (-0.6, 0.7)
2021								
Total	48.4 (48.0, 49.0)	49.0 (48.5, 49.5)	-0.6 (-1.1, -0.1)	0.0 (-0.5, 0.6)	52.4 (51.8, 53.0)	52.6 (52.1, 53.2)	-0.6 (-1.2, -0.1)	0.4 (-0.1, 1.0)
With Disability	3.4 (2.9, 3.9)	3.6 (3.1, 4.1)	-0.1 (-0.6, 0.3)	0.0 (-0.5, 0.5)	5.0 (4.2, 5.7)	5.0 (4.3, 5.8)	-0.2 (-0.9, 0.5)	0.1 (-0.6, 0.8)
2022								
Total	48.5 (48.0, 49.1)	49.2 (48.6, 49.7)	-0.3 (-0.8, 0.3)	-0.4 (-1.0, 0.1)	52.2 (51.7, 52.8)	52.7 (52.2, 53.3)	-0.3 (-0.9, 0.3)	-0.2 (-0.8, 0.3)
With Disability	3.5 (2.9, 3.9)	3.6 (3.2, 4.2)	-0.1 (-0.5, 0.4)	-0.1 (-0.6, 0.4)	4.9 (4.2, 5.7)	5.1 (4.4, 5.9)	-0.1 (-0.8, 0.6)	-0.1 (-0.8, 0.6)
**United Kingdom**								
2019								
Total	45.3 (45.1, 45.4)				49.2 (49.1, 49.3)			
With Disability	4.7 (4.6, 4.8)				6.3 (6.2, 6.3)			
2020								
Total	43.8 (43.6, 44.0)	45.3 (45.1, 45.4)	-1.6 (-1.8, -1.4)	0.1 (-0.1, 0.3)	48.2 (48.1, 48.3)	49.2 (49.0, 49.3)	-1.2 (-1.4, -1.1)	0.2 (0.1, 0.4)
With Disability	4.3 (4.3, 4.4)	4.7 (4.6, 4.8)	-0.4 (-0.5, -0.3)	0.0 (0.0, 0.1)	6.0 (5.9, 6.1)	6.3 (6.2, 6.4)	-0.4 (-0.5, -0.3)	0.1 (0.0, 0.2)
2021								
Total	43.9 (43.7, 44.1)	45.3 (45.1, 45.5)	-1.3 (-1.6, -1.1)	-0.1 (-0.3, 0.2)	48.3 (48.2, 48.5)	49.2 (49.0, 49.4)	-1.3 (-1.5, -1.2)	0.5 (0.3, 0.6)
With Disability	4.4 (4.3, 4.5)	4.8 (4.7, 4.9)	-0.4 (-0.5, -0.3)	0.0 (-0.1, 0.1)	6.1 (6.0, 6.2)	6.4 (6.3, 6.5)	-0.5 (-0.5, -0.4)	0.2 (0.1, 0.3)
2022								
Total	44.5 (44.2, 44.7)	45.4 (45.1, 45.7)	-0.5 (-0.8, -0.2)	-0.4 (-0.7, -0.2)	48.6 (48.4, 48.8)	49.2 (49.0, 49.4)	-0.6 (-0.8, -0.4)	-0.1 (-0.2, 0.1)
With Disability	4.6 (4.5, 4.7)	4.8 (4.7, 4.9)	-0.2 (-0.3, -0.1)	-0.1 (-0.2, 0.0)	6.2 (6.1, 6.3)	6.5 (6.4, 6.6)	-0.2 (-0.3, -0.2)	0.0 (-0.1, 0.1)

Figure J: Life-expectancy at age 35 by gross domestic product per capita, 2019-2022

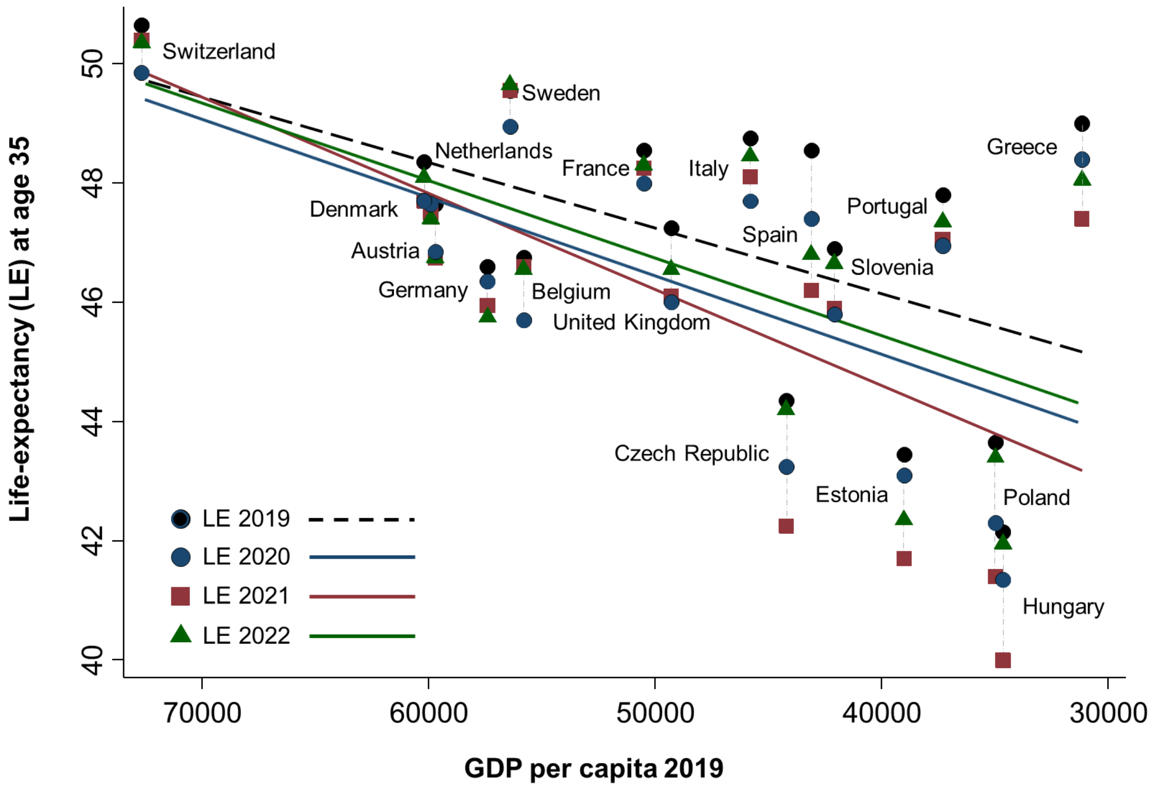

References

1. Borsch-Supan A, Brandt M, Hunkler C, Kneip T, Korbmacher J, Malter F, et al. Data Resource Profile: the Survey of Health, Ageing and Retirement in Europe (SHARE). Int J Epidemiol. 2013;42(4):992-1001. Epub 2013/06/20. doi: 10.1093/ije/dyt088. PubMed PMID: 23778574; PubMed Central PMCID: PMCPMC3780997.
2. Börsch-Supan A. Survey of Health, Ageing and Retirement in Europe (SHARE) Wave 1. Release version: 7.1.0. SHARE-ERIC. Data set. DOI: 10.6103/SHARE.w1.710. 2020.
3. Börsch-Supan A. Survey of Health, Ageing and Retirement in Europe (SHARE) Wave 2. Release version: 7.1.0. SHARE-ERIC. Data set. DOI: 10.6103/SHARE.w2.710. 2020.
4. Börsch-Supan A. Survey of Health, Ageing and Retirement in Europe (SHARE) Wave 4. Release version: 7.1.0. SHARE-ERIC. Data set. DOI: 10.6103/SHARE.w4.710. 2020.
5. Börsch-Supan A. Survey of Health, Ageing and Retirement in Europe (SHARE) Wave 5. Release version: 7.1.0. SHARE-ERIC. Data set. DOI: 10.6103/SHARE.w5.710. 2020.
6. Börsch-Supan A. Survey of Health, Ageing and Retirement in Europe (SHARE) Wave 6. Release version: 7.1.0. SHARE-ERIC. Data set. DOI: 10.6103/SHARE.w6.710. 2020.
7. Börsch-Supan A. Survey of Health, Ageing and Retirement in Europe (SHARE) Wave 7. Release version: 7.1.1. SHARE-ERIC. Data set. DOI: 10.6103/SHARE.w7.711. 2020.
8. Banks J, Batty, GD., Coughlin, K., Dangerfield, P., Marmot, M., Nazroo, J., Oldfield, Z., Steel, N., Steptoe, Wood, M., Zaninotto, P. (2019). English Longitudinal Study of Ageing: Waves 0-9, 1998-2019. [data collection]. 33rd Edition. UK Data Service. SN: 5050, http://doi.org/10.5255/UKDA-SN-5050-20.
9. Chertkow H, Nasreddine Z, Joanette Y, Drolet V, Kirk J, Massoud F, et al. Mild cognitive impairment and cognitive impairment, no dementia: Part A, concept and diagnosis. Alzheimers Dement. 2007;3(4):266-82.
10. Massoud F, Belleville S, Bergman H, Kirk J, Chertkow H, Nasreddine Z, et al. Mild cognitive impairment and cognitive impairment, no dementia: Part B, therapy. Alzheimers Dement. 2007;3(4):283-91.
11. Ahmadi-Abhari S, Guzman Castillo M, Bandosz P, Shipley MJ, Muniz Terrera G, Singh-Manoux A, et al. Temporal trend in dementia incidence since 2002 and projections for prevalence in England and Wales to 2040: modelling study. BMJ. 2017;358:j2856.
12. Bandosz P, Ahmadi-Abhari S, Guzman-Castillo M, Pearson-Stuttard J, Collins B, Whittaker H, et al. Potential impact of diabetes prevention on mortality and future burden of dementia and disability: a modelling study. Diabetologia. 2020;63(1):104-15. Epub 20191115. doi: 10.1007/s00125-019-05015-4. PubMed PMID: 31732789; PubMed Central PMCID: PMCPMC6890625.
13. Guzman Castillo M, Ahmadi-Abhari S, Bandosz P, Shipley MJ, Capewell S, Singh-Manoux A, et al. Forecasted trends in disability and life expectancy in England and Wales up to 2025: a modelling study. Lancet Public Health. 2017;2(7):e307-e13.
14. Caporali A, Garcia J, Couppié É, Poniakina S, Barbieri M, Bonnet F, et al. The demography of COVID-19 deaths database, a gateway to well-documented international data. Sci Data. 2022;9(1):93. Epub 20220322. doi: 10.1038/s41597-022-01191-y. PubMed PMID: 35318326; PubMed Central PMCID: PMCPMC8940928.
15. Human Mortality Database. University of California BU, and Max Planck Institute for Demographic Research (Germany). Available at www.mortality.org.
16. Pascariu MD, Dańko MJ, Schöley J, Rizzi S. ungroup: An R package for efficient estimation of smooth distributions from coarsely binned data. Journal of Open Source Software. 2018;3(29). doi: https://doi.org/10.21105/joss.00937.
17. UK Office for National Statistics. Updated estimates of coronavirus (COVID-19) related deaths by disability status, England: 24 January 2020 to 9 March 2022. Available from: https://www.ons.gov.uk/peoplepopulationandcommunity/birthsdeathsandmarriages/deaths/articles/coronaviruscovid19relateddeathsbydisabilitystatusenglandandwales/24january2020to9march2022.
18. Global Burden of Disease Collaborative Network. Global Burden of Disease Study 2019 (GBD 2019) Results. Seattle USIfHMaEI, 2020. Available from http://ghdx.healthdata.org/gbd-results-tool.
19. World Health Organization. Available from: https://covid19.who.int/WHO-COVID-19-global-data.csv
