# Supplementary material for: Direct and indirect impacts of the COVID-19 pandemic on life expectancy and person-years of life lost with and without disability: A systematic analysis for 18 European countries, 2020–2022
Source: PLoS Med. 2025 Mar 11;22(3):e1004541. doi: 10.1371/journal.pmed.1004541 (PMC11896051; doi:10.1371/journal.pmed.1004541)
Supplement: Attachment — Submitted filename: Response to Reviewers.pdf [file pmed.1004541.s002.pdf]

Comments from the academic editor:

The academic editor thought that the manuscript looked interesting and relevant. She commented that, whilst the reviewers raised many points, none doubted the value of the work. She was supportive of taking your manuscript forward to the next step.

Comments from the reviewers:

Reviewer #1: The authors attempted to assess the direct and indirect impacts of the COVID-19 pandemic on life expectancy and years of life lost with and without disabilities in 18 European countries, 2020-2022. The authors have been quite successful in achieving their objective. In fact, I only have two comments.

Thank you for your time to review our manuscript and your valuable feedback.

1. As the authors state on page 3 of the Supplementary material, they estimate transition probabilities by fitting Cox proportional hazards regression models. However, the authors do not test the fulfillment of the proportional hazards assumption. They should test it (both globally and individually for each predictor) and also provide the corresponding Kaplan-Meier curves.
   If it is not fulfilled, they should use methods other than the Cox model.

Our Response: Thank you for your feedback. The proportional hazards assumptions for all Cox-proportional hazards regression analyses were tested using Kaplan-Meier curves and plots of Schoenfeld residuals. The assumptions were met for all of the regressions and the Schoenfeld residuals showed random patterns over time for all Cox-regressions. The Cox-regression analyses were conducted to derive transition probabilities for incidence of disease and disability, the calendar trends in transition probabilities, and hazard ratios of death from

each state. The outputs from the Cox-regression models were then used as input data to inform the Markov models. The corresponding standard errors were used in Monte-Carlo simulations to derive uncertainty intervals.

For the Markov model for each country, 17 Cox-proportional hazards regression models were fitted (please see Figure 1 for an illustration of transition probabilities) and we modelled data for 18 countries. Please kindly note given the 17 Cox models for each country, and 18 countries, providing all corresponding Kaplan-Meier curves would result in 306 Figures for unstratified models, and an additional 712 Figures for analysis stratified by sex. All Cox models were fitted after checking the assumptions of the model are met. We would be happy to provide any specific Kaplan-Meier curves related to particular Cox-regression models that you would like to inspect. If the Editors would like to have all Kaplan-Meier Figures added to Supplementary material (now renamed S1 Appendix), we would be happy to provide the Figures.

2. In the Discussion, the authors point out, as a limitation, that 'another source of error is the accuracy of projections for future mortality rates which is inherently unpredictable' and that 'to partly account for uncertainty in projections in mortality and incidence of disease we accompanied our estimates with 95% uncertainty intervals derived from Monte-Carlo simulations'.
   I think this is good, but insufficient. The authors should discuss uncertainty in sufficient detail. That is, for example, are the intervals wider in different countries? Are the intervals different over time? etc.

Our Response: Thank you for pointing out that our methods to account for uncertainty have not been presented in sufficient detail. We have added details in response to your queries. Please see the manuscript Methods Page 13, line 279 and the S1 Appendix Methods section SM6 on page 13.

"*Probabilistic sensitivity analysis*

To explore the impact of parameter uncertainty on model outputs, we conducted a probabilistic sensitivity analysis using Monte Carlo simulation. The procedure entails sampling from specified distributions for the input parameters that were used in the model for each data cycle. We calculated 500 iterations to estimate 95% uncertainty intervals for output variables. Uncertainty in model parameters increases over time and varies between countries. Further details of the probabilistic sensitivity analysis can be found in S1 Appendix pp 13-14 and the statistical formula are provided in S1 Appendix pp 3-8."

*"**SM6: Probabilistic Sensitivity Analysis (Monte Carlo simulation)***

To derive uncertainty intervals for all outputs we ran Monte Carlo simulations with 500 iterations. Each input parameter of the Markov models, including baseline prevalence estimates, transition probabilities, and the calendar effects for transition probabilities, is entered as a normal distribution with mean and standard errors, which are derived from the methods described above. In each iteration of the Monte Carlo simulation, each input parameter is randomly selected from these normal distributions. The 2.5 and 97.5 percentile of the distribution of outputs obtained from 500 iterations constitute the 95% uncertainty intervals. For further details, please see mathematical calculations presented in the Table above under the "Statistical Methods" section.

The width of the 95% uncertainty intervals depend on the standard error of the input values. The standard errors of the transition probabilities increase with calendar years since baseline, due to multiplication of transition probabilities by calendar effects in each year (mathematical calculations presented above). This roughly translates to standard errors for transition probabilities increasing by a factor of the square root of years since baseline. All input data and the standard errors for the input parameters were derived from country specific data. The standard errors for input estimates were larger in countries where data is derived from smaller sample sizes. Also, in countries or age-groups where there are larger year-on-year fluctuations in the incidence of disease, disability, or mortality rates (i.e., there is larger deviation in observations from the line of best fit) the standard errors from the transition probabilities obtained from the Cox-regression models were larger, leading to wider uncertainty intervals."

Reviewer #2: Our knowledge of the impact of the pandemic on mortality is still fragmentary, despite a large number of publications on COVID-19. The manuscript aims to provide useful information by providing estimates of person-years of life lost (PYLL) and quantifying them by disability status. The authors use an excess mortality approach with the baseline mortality level estimated using the multi-state Markov chain model. Nevertheless, I have some doubts about the outcome.

We thank you for your time reviewing our manuscript and for your valuable feedback which has helped us improve the manuscript.

1. Relatively old data (last year 2017) is used to estimate disability-free proportion. The assumption that the pandemic didn't change any trends in disability (especially at most affected old ages) is relatively strong and not necessarily correct.

Our Response: We thank you for your feedback. We understand the original version of our manuscript was unclear regarding this point. We did not make the assumption of no change in disability trends following the pandemic in our modelling. As you have pointed out, changes in disability trends during and after the pandemic would affect projections for prevalence of disability and years of life expected to be lived by disability status following the onset of the pandemic for living persons. However, we assessed the life-years lost due to excess deaths in 2020-2022, defined as the years of life with and without disability that would have been expected to be lived if the pandemic had not happened and pre-pandemic trends had continued, that were "lost" due to premature deaths during 2020-2022. Changes in disease and disability trends after the onset of the pandemic would not affect years of life that would have been expected to be lived if the pandemic had not happened. We have clarified this point in the manuscript page 10, line 201 (quoted section below).

"*The PYLL due to the pandemic related excess deaths in 2020-2022 were defined as the years of life that would have been expected to be lived if the pandemic had not happened. We therefore ran and compared outputs from three Markov models. Model 1: To estimate person-years of life with and without disability that would have been expected to be lived in the absence of the pandemic, we included transition probabilities and expected mortality rates following pre-*

pandemic trends as model inputs. Model 2: To estimate loss in years of life due to registered COVID-19 deaths, we adjusted expected mortality rates from Model 1 for the additional registered deaths due to COVID-19 by age and sex for the years 2020-2022. Model 3: To estimate total loss in person-years of life due to deaths from any cause that would not have been expected under pre-pandemic trends, we applied mortality rates by disease and disability status that were derived from observed all-cause mortality rates by age and sex for the years 2020 to 2022. The difference in person-years of life between models 2 and 3 with model 1 provide an estimate for PYLL due to COVID and non-COVID deaths based on numbers of years that would have been expected to be lived with and without disability if premature deaths directly or indirectly related to the pandemic had not occurred and the pre-pandemic trends had continued. Further details are provided in S1 Appendix pages 3-14."

We assumed that higher incidence of disease and disability during the pandemic would lead to higher mortality rates. To ensure we accurately account for disproportionately higher mortality rates amongst persons with disease and disability during 2020-2022, we conducted careful analyses of mortality rates by disease and disability status. Please see the revised version for details of the methods to derive expected and observed mortality rates by disease and disability status which are explained in the methods section subtitled "*Analyses by disability status"* on page 11, line 236, with details presented in S1 Appendix pages 11 and 12 in sections subtitled "*Mortality rates by disease and disability status"* and "*Assessment of the reliability of the method for estimation of mortality rates by disability status"*. The methods to assess mortality by disability status are discussed in the discussion page 17, line 405 to 424.

In response to your feedback regarding the use of data collected to 2017, we have added a paragraph to clarify and discuss availability of data from ELSA and SHARE studies to year 2017. Please see the revised manuscript page 21, lines 527-541.

"*Life-years lost for excess deaths in 2020-2022 were calculated based the counter-factual scenario in which the pandemic did not happen. Our modelling strategy accounts for changes in mortality, incidence of cardiovascular disease, cognitive impairment, and disability over time by incorporating calendar trends based on pre-pandemic trends in the calculations. We found that in all countries, the trends in mortality and incidence of disease and disability followed linear or log-linear trends over time over the two decades prior to the pandemic. We*

thus assumed the most likely scenario in the absence of the pandemic would have been the continuation of these trends. To obtain incidence and pre-pandemic trends in incidence of disease and disability, we used data from the ELSA and SHARE studies for the years 2002-2017 that were available at time of analysis. Although, it would have been ideal to use data that spans to the year 2019, incidence of disease and mortality have shown steady trends over time. Therefore, obtaining pre-pandemic trends based on data collected to the year 2017 rather than 2019 is unlikely to have introduced a major bias in our findings. We accounted for uncertainty in future projections for input parameters, including the incidence of disease, disability and mortality rates, in Monte Carlo simulations."

2. The period used to fit the model (since 1998) might be too long. This would be OK for mid- and long-term forecasts. The short-term forecast used to estimate the expected level of mortality in 2020-2022 is more sensitive to recent changes in mortality trends.

Our Response: Thank you for your suggestion. We found that the mortality rates by age-group and sex in each country followed log-linear trends during 1998-2019. We used the fitted smoothed lines to predict the expected change in mortality rates for each year compared to the previous year as described in S1 Appendix page 10, section "*Projections for expected mortality rates*". Since mortality rates by age and sex followed log-linear patterns over time (please see S1 Appendix Figure B, pp 20-25), the length of observation period used for deriving the estimates does not have a remarkable effect on short term predictions of expected mortality rates. However, using a shorter observation period would underestimate the uncertainty in the predictions. As there are less fluctuations in mortality rates in a shorter observation period, the resulting standard errors for predictions of expected mortality rates would be smaller, consequently leading to narrower uncertainty intervals.

As demonstrated in S1 Appendix Figure B, the expected rate of change in mortality rates from 2019 to 2020-2022 is small and in line with average changes from 2017 to 2019. To quantitatively demonstrate the derived expected mortality rates in 2020-2022 compared to those observed in the preceding years, we present below "as an example" the observed

mortality rates for 2017-2019 and expected mortality rates for 2020-2021 in woman aged 65-69, from 3 countries that had that the highest, median, and lowest person-years of life lost per capita in 2020-2022.

**Country**	**Mortality rates in women aged 65-69**					
	2017-observed	2018-observed	2019-observed	2020-expected	2021-expected	2022-expected
**Estonia**	0.01036	0.01175	0.00957	0.00948	0.00941	0.00936
**United Kingdom**	0.00966	0.00965	0.00934	0.00933	0.00932	0.00927
**Sweden**	0.00856	0.00856	0.00772	0.00762	0.00751	0.00733

The difference in observed mortality rates for 2019 and estimates for those expected for 2020-2022 are reflected in estimates of expected life-expectancy in the absence of the pandemic (Figure 7 of the manuscript, and S1 Appendix Table J), and the average change in life-expectancy over time displayed in S1 Appendix Figure J. The changes in life-expectancy expected in the absence of the pandemic are small, showing the projected mortality rates for the years 2020-2022 are very close to rates observed in 2019.

3. In 2020, the number of COVID-19 deaths by country is not comparable between countries. At the beginning of the pandemic, information on deaths from COVID-19 was biased by large differences in approaches to testing SARS-CoV-2 and recoding of COVID-19 as a cause of death. While some countries tend to attribute to COVID-19 all or nearly all deaths of those with positive tests for the virus, others applied more conservative approaches with an emphasis on pre-existing co-morbidities.

Our Response: Thank you for raising this point, which we fully agree with. Please see the manuscript Discussion page 18, lines 435-473 where this issue is described and the relevant implications for our findings are discussed. Below we provide some excerpts from the discussion on the topic:

"Distinguishing between deaths attributable to COVID-19 and those unrelated is challenging due to variations in cause-of-death determination and registration systems across European countries [32]. Varying testing rates and differences in criteria for designation of COVID-19 as cause of death over time and location have complicated estimation of the direct and indirect impacts of the pandemic and cross-country comparisons".

"There was a notable burden of PYLL not explained by registered COVID-19 deaths." "We observed a decline in PYLL due to COVID-19 deaths after 2021, coinciding with the introduction of vaccines. However, PYLL due to other causes continued to increase from 2020 to 2022 in most of the 18 countries. As the pandemic progressed, most countries improved testing coverage, and expanded the definition and data sources for COVID-19 case of death determination, making under-registration of COVID-19 as cause of death unlikely to explain the increasing PYLL due to non-COVID mortality after 2021".

"We observed lower-than-expected non-COVID mortality rates at different time points in Sweden, France, Italy, Belgium, Switzerland, Denmark, and the UK. This finding aligns with previous findings [2, 33-35]. Several reports indicate the possibility that COVID-19 containment measures, such as wearing masks and social distancing, may be linked to reduction in other causes of death, such as the flu, pneumonia or infectious diseases which share similar prevention strategies [33-36]. On the other hand, non-COVID-19 deaths in patients with underlying terminal conditions, such as malignant cancers, may have been misclassified as COVID-19 deaths among those with a concurrent positive test [33-35]. Analysis of COVID-positive death certificates collected by the Italian National Statistics Institute in the municipality of Rome found other causes of death, such as cardiovascular diseases, malignant tumors, and diseases of the respiratory system, in 20% of the certificates [34]. Our observation of lower-than-expected non-COVID mortality rates could therefore be partly explained by overestimation of COVID-19 deaths or misclassification of deaths due to other causes as COVID-19 deaths, consequently underestimating non-COVID mortality. Moreover, countries employing broad definitions for COVID-19, including clinical symptoms and high-risk contacts without PCR test confirmation, may have led to an overestimation of COVID-19 deaths at certain time-points [35]. To address this challenge, we quantified PYLL using two extreme scenarios: 1) all reduction in non-COVID deaths was genuine, and 2) all this reduction resulted from attributing some non-COVID deaths to COVID-19. Assuming the

true impact lies between these two extreme scenarios, we estimate that on average between 22% (scenario 1) and 31% (scenario 2) of the excess life years lost during 2020-2022 were due to non-COVID mortality, with significant variation among countries."

4. The importance of GDP seems to be overestimated by the authors. For example, several publications noted an East-West gradient in pandemic losses in 2021. It corresponds to the same gradient in mortality. The correlation between GDP and mortality was described by Presto many years ago but it doesn't explain why Western countries had lower losses in 2021. In addition, the correlation between GDP and losses doesn't work in 2020. In this light, factors such as vaccination coverage and trust in science and government seem to be more important.

Our Response: Thank you for your suggestion. We examined the socio-economic differences in life-years lost between countries as one of the study aims. We used GDP per capita as a simple proxy summary metric for this purpose.

We agree that vaccination coverage, geographical distribution, trust in science, and compliance with policy guideline between countries are important determinants.

According to your suggestion, we have included an analysis of the effect of vaccination coverage on life-years lost between countries, please see the revised Figure 6 and S1 Appendix Figure H. We observed an inverse association between vaccination coverage and person-years of life lost from both COVID and non-COVID registered causes of death. This observation may be explained by vaccination coverage being a proxy of the quality of health services in the country. Of interest, we found that the deviations in the person-years of life lost compared to what is expected from the countries' GDP mirrored the vaccination coverage. Please see Results page 15, lines 352-357.

"*Total PYLL per capita was inversely and independently associated with the countries' GDP per capita and average doses of vaccine administered per adult population (Figure 6). Countries with higher GDP per capita and higher vaccination rates experienced lower PYLL due to both COVID and non-COVID causes (S1 Appendix Figure H, p 43). The deviation in*

the countries' PYLL from that explained by GDP per capita mirrored the average doses of administered COVID19 vaccines per adult population (Figure 6)."

Figure 6: Associations between person-years of life lost per 1000 population over the years 2020-2022 with Gross Domestic Product per capita and vaccination coverage

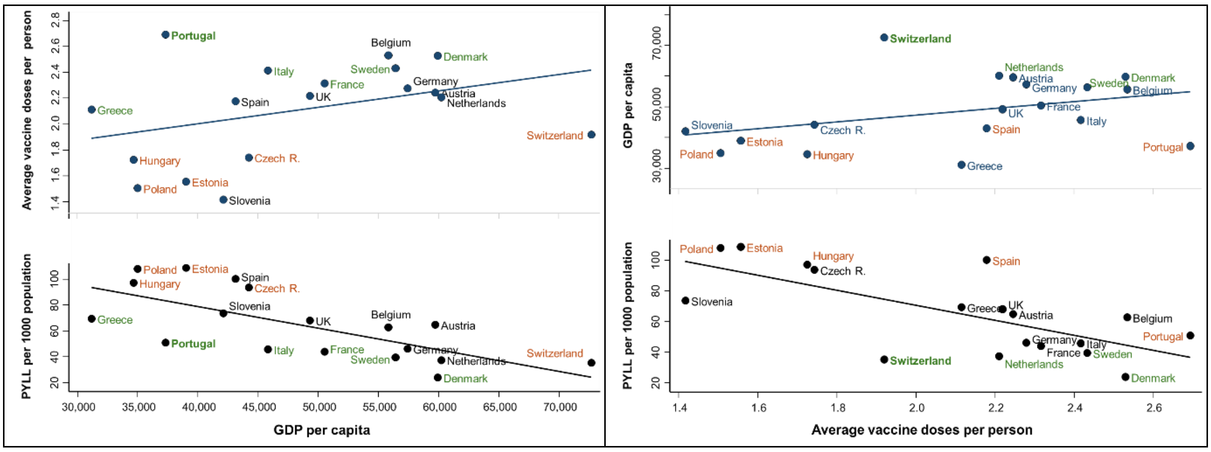

S1 Appendix Figure H: Person-years of life lost per 1000 population over the years 2020-2022 by vaccination coverage and 2019 Gross Domestic Product per capita

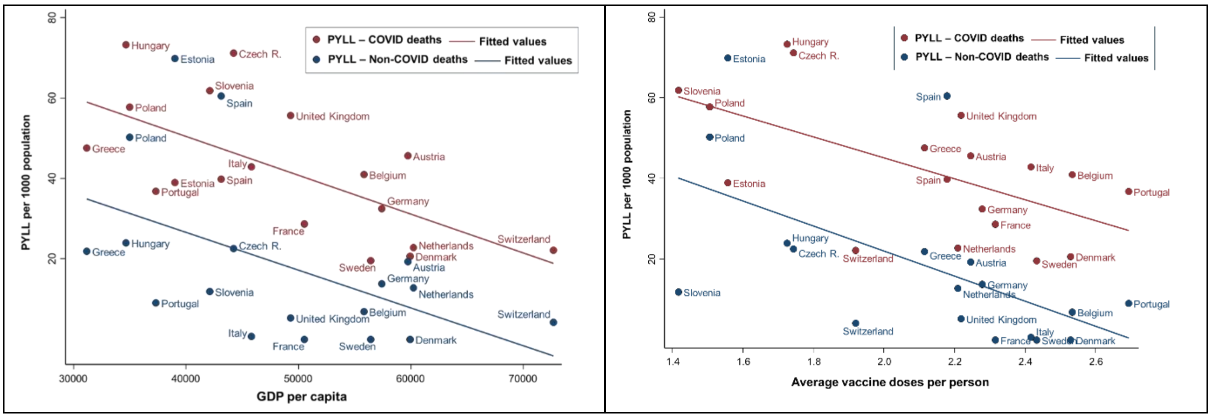

The association between PYLL and vaccination coverage is discussed in the Discussion page 20, lines 486-495:

"We found COVID vaccination coverage was inversely associated with PYLL, independent of GDP. Several countries, notably Portugal, Italy, Denmark and Sweden, had lower PYLL than countries with similar GDP per capita and the lower PYLL mirrored higher vaccination coverage in these countries. On the other hand, several countries including Poland, Estonia,

Hungary, Czech Republic and Switzerland, had higher PYLL than countries with similar GDP per capita, also mirroring the lower vaccination coverage in these countries. COVID vaccination coverage was inversely associated with PYLL related to non-COVID deaths as well as those associated with COVID deaths, indicating that vaccination coverage may be a marker of the quality and reach of healthcare in the country, as well as the compliance of the population with government health policy and guidelines."

It is difficult to quantify the East - West trend in life-years lost, as several groups of countries are on the same geographical longitude, and we do not see a clear trend. Please see Figure below.

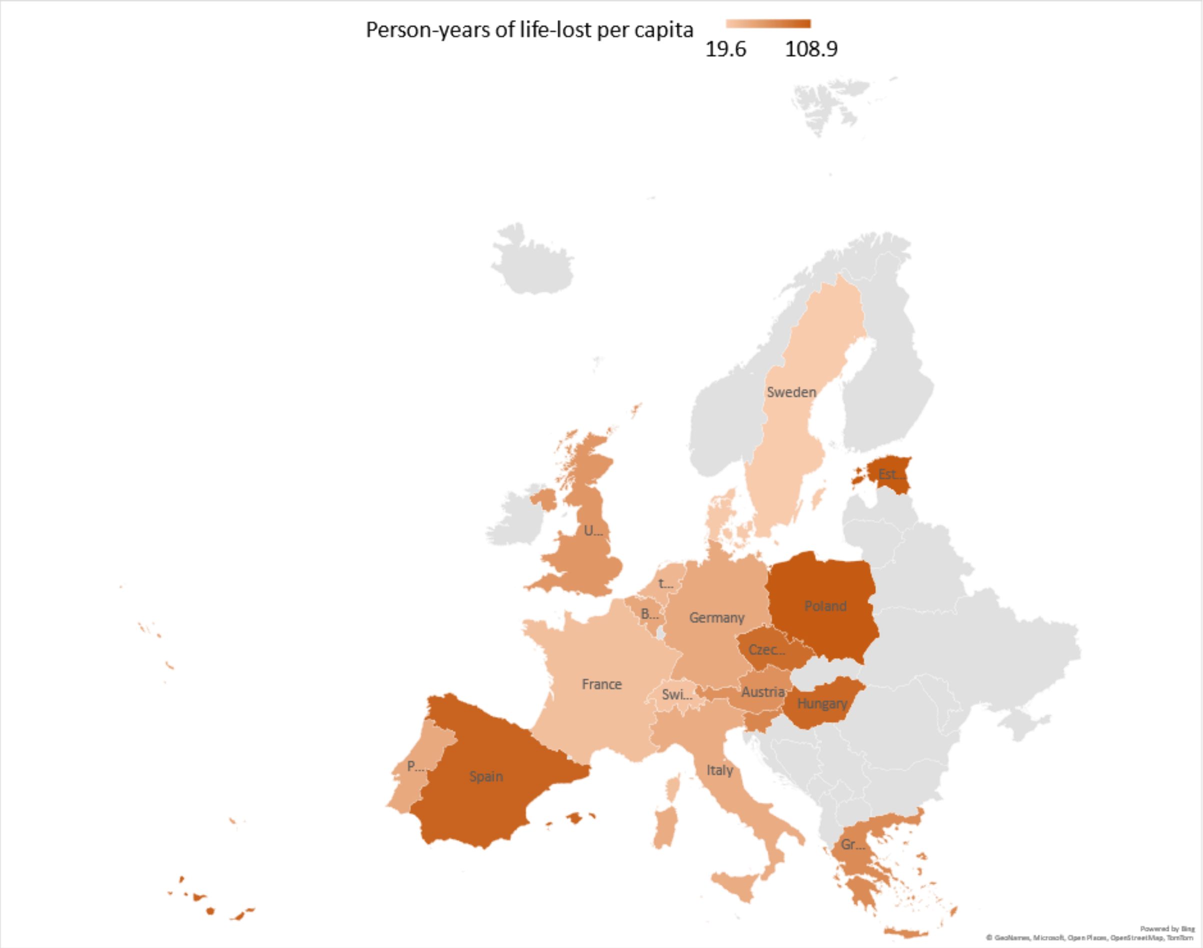

To objectively examine and quantitatively report the association of life-years lost with the population trust in science and government advice, we would require objective summary metrics for these parameters that are assessed using similar methods in all countries. Unfortunately, these data were not available to us at present. However, in the Discussion page 20, lines 486-495 (quoted text above) we have noted that the inverse association of vaccination coverage with PYLL due to non-COVID deaths may be an indicator of the compliance of the population with health policy and guidelines as well as the quality and reach of the health system.

> Finally, the description of the model in the Supplementary material is not detailed enough and is poorly structured. It is impossible to evaluate the model and its possible limitations.

Our Response: We thank you for bringing this issue to our attention. The Supplement material was intended to provide additional detail to the Methods described in the manuscript. We agree it would be useful for the Supplement to provide a stand-alone description of the Markov methods in detail. In our revision, we restructured the Supplement (renamed S1 Appendix in line with journal requirements) and included the statistical and mathematical formula for each step of the Markov model calculations accompanied with descriptions in S1 Appendix pages 3-14. Each step of the Markov model is signposted to the section where the methods to derive inputs or outputs are described under appropriate explanatory subheadings. The description is followed by a Table outlining all model assumptions and limitations (S1 Appendix Table A). Further discussions on the limitations of the Markov models are provided in the manuscript Discussion.

We hope that the revision addresses your concern regarding this point. We are happy to provide further clarifications if any sections are not clear.

Reviewer #3:

1- While the use of a Markov model is appropriate for modeling longitudinal transitions, could the authors provide more justification for choosing this model over other potential approaches (e.g., Cox proportional hazards models for time-to-event data or microsimulation models)? Even though the Markov model captures state transitions well, a brief explanation for this choice, particularly given the model's memoryless property (i.e., transitions depend only on the current state and not on past history), would be beneficial.

Our Response: We thank you for your time reviewing our manuscript and for your valuable feedback which has helped us improve the manuscript.

With regards to the modelling strategy, the Markov modelling strategy was adopted as the method that would enable us to calculate the person-years of life lost whilst accounting for dynamic trends in disease incidence and mortality over time, and in balance with computational intensity. To account for dynamic trends in disease and mortality, which act as competing risks for one another (for example, declining cardiovascular disease incidence and mortality leads to higher life expectancy and larger numbers at risk of disability at old age), we used a multi-state modelling strategy. Although we have used Cox-proportional hazard models and time series analyses to derive transition probabilities and calendar trends in mortality rates that were incorporated in the Markov models, these methods alone would not enable us to integrate trends in incidence of CVD, dementia, disability which interact as competing risks.

In the choice between micro-simulation and macro-simulation (Markov modelling) strategies, we decided based on data availability and computational intensity. In the Markov models, we included 8 states to capture progression of the population to disability from the most prevalent determinants. Using the Markov strategy, the population was divided to 130 strata (by sex and single year of age ranging from 35 to 100) in 8 health states for each country, whilst microsimulation would have required running on population samples of up to 18,000 people in each country. We did not implement microsimulations due to the exponential increase in computational intensity which would render the project unfeasible.

With regards to the memory-less property of Markov models, we accounted for this attribute in our methods and data sources to derive input data. The transition probabilities were derived from SHARE and ELSA which are broadly representative samples of the population

after applying survey weights. Therefore, estimates for risks of dementia, cardiovascular disease, disability and death obtained from SHARE and ELSA for each single year of age and sex strata reasonably represent a weighted average of risk levels across the spectrum of the severity of these conditions, comorbidities, and risk factors. Using the same data sources, our results are not likely to be different from a microsimulation approach. By age and sex, the weighted average transition probability multiplied by the number of people in a health state (our Markov models) is mathematically equivalent to the sum of the individualized transition calculations (achieved in microsimulation) using the same data sources (please see S1 Appendix Table A).

We designed the Markov models with the health states to best account for the purposes of the study. We therefore included health states in the model which were drivers or competing risks for disability as described in the methods page 8, lines 128-137. Notwithstanding between country variations, around a half of disability co-occurred with cardiovascular disease, about 25% with dementia, and 25% with other causes. We therefore have 4 health states in the model that represent disability co-occurring with cardiovascular disease (CVD), with dementia, with CVD and dementia, or with other causes (including disability with no registered underlying cause). Please see Figure 1 for details. In the revised version we have added further details, including the mathematical equations, for the Markov method in S1 Appendix pages 3-8.

In summary, we found the Markov strategy was the most Suitable method to achieve project aims, account for dynamic changes in disease trends, and interactions between competing risks, whilst being computationally feasible for a project of this magnitude.

2- The manuscript outlines the assumptions of the model clearly. However, I suggest including a brief discussion of the limitations these assumptions might impose, especially regarding the transition probabilities derived from datasets that predate the pandemic.

Our Response: We are pleased to hear the description of assumptions has been clear and thank you for raising the issue with the dates of data collection. We understand the original version of our manuscript was unclear regarding the disease trends incorporated in the

models. We have clarified this point in the revised Methods page 10, lines 201-222. In summary, we defined the person-years of life lost due to excess mortality during 2020-2022 as the years of life that would have been expected to be lived if the pandemic had not happened. In the absence of the pandemic, the most likely scenario would have been the continuation of previously observed trends. We used data available from ELSA and SHARE that were collected from 2002 to the year 2017 to obtain transition probabilities and calendar trends to estimate the life-years that would have been expected to be lived if the pandemic had not happened and the pre-pandemic trends had continued. Although it would have been ideal to have data that spans to the year 2019 for this purpose, given that incidence of disease and mortality had shown steady trends over the two decades preceding the pandemic, availability of data to the year 2017 rather than 2019 is unlikely to have a remarkable impact on our findings. We have acknowledged this limitation of the study in the discussion page 21, lines 527-541.

With regards to the limitations of the study resulting from the assumptions underlying the Markov models, we have provided details of how we checked that the assumptions are reasonably met in the second column of S1 Appendix Table A. All input data were entered as a range of values (denoted by mean and standard error) to account for parameter uncertainty in the assumptions made, and the corresponding uncertainty intervals derived from Monte Carlo simulations have been presented for all outputs. The limitations of the study related to the underlying assumptions of the model are discussed in the manuscript Discussion, please see paragraphs starting from lines 405, 504, 527, and 542.

Methods page 10, lines 201-222:

"*The PYLL due to the pandemic related excess deaths in 2020-2022 were defined as the years of life that would have been expected to be lived if the pandemic had not happened. We therefore ran and compared outputs from three Markov models. Model 1: To estimate person-years of life with and without disability that would have been expected to be lived in the absence of the pandemic, we included transition probabilities and expected mortality rates following pre-pandemic trends as model inputs. Model 2: To estimate loss in years of life due to registered COVID-19 deaths, we adjusted expected mortality rates from Model 1 for the additional registered deaths due to COVID-19 by age and sex for the years 2020-2022. Model 3: To estimate total loss in person-years of life due to deaths from any cause that would not have*

been expected under pre-pandemic trends, we applied mortality rates by disease and disability status that were derived from observed all-cause mortality rates by age and sex for the years 2020 to 2022. The difference in person-years of life between models 2 and 3 with model 1 provide an estimate for PYLL due to COVID and non-COVID deaths based on numbers of years that would have been expected to be lived with and without disability if premature deaths directly or indirectly related to the pandemic had not occurred and the pre-pandemic trends had continued. Further details are provided in S1 Appendix pages 3-14.

To accurately estimate the expected prevalence of disability and mortality in the absence of the pandemic (Model 1), all transition probabilities entered in the Markov models were calendar-year specific following pre-pandemic trends in incidence of disease, disability, and mortality. The calendar effects for age-sex-specific trends in incidence of cardiovascular disease, cognitive impairment/dementia, and functional impairment (disability), were quantified using ELSA and SHARE data. Further details are provided in S1 Appendix (pp 12-13).”

Discussion page 21, lines 527-541:

“Life-years lost for excess deaths in 2020-2022 were calculated based on the counter-factual scenario in which the pandemic did not happen. Our modelling strategy accounts for changes in mortality, incidence of cardiovascular disease, cognitive impairment, and disability over time by incorporating calendar trends based on pre-pandemic trends in the calculations. We found that in all countries, the trends in mortality and incidence of disease and disability followed linear or log-linear trends over time over the two decades prior to the pandemic. We thus assumed the most likely scenario in the absence of the pandemic would have been the continuation of these trends. To obtain incidence and pre-pandemic trends in incidence of disease and disability, we used data from the ELSA and SHARE studies for the years 2002-2017 that were available at time of analysis. Although, it would have been ideal to use data that spans to the year 2019, incidence of disease and mortality have shown steady trends over time. Therefore, obtaining pre-pandemic trends based on data collected to the year 2017 rather than 2019 is unlikely to have introduced a major bias in our findings. We accounted for uncertainty in future projections for input parameters, including the incidence of disease, disability and mortality rates, in Monte Carlo simulations.”

3- The use of population numbers and mortality rates from sources like STMF and the WHO is appropriate, and the explanation of these inputs is clear. However, it would be helpful to explain more clearly how country-specific variations in data availability and quality were handled, as these differences could affect the robustness of the model's outputs.

Our Response: We have responded to points 3 and 4 together. Please see the response to point 4 below.

4- The use of ELSA and SHARE data to estimate transition probabilities is well justified. However, the manuscript would benefit from including sensitivity analyses that account for variability in these inputs, as the reliability of data may differ between countries.

Our Response: We are pleased that you agree with our choice of data sources and thank you for your queries regarding the between country differences in the reliability of data. We aimed to use data sources that were harmonized and standardized to enable between country comparisons. We used data from the ELSA and SHARE studies as the data collection protocol is exactly the same between countries. We only included countries where this data was available for at least two data collection waves, leading to list of the 18 countries. We do not have any reason to suspect that the reliability of the ELSA and SHARE data varies between countries.

Similarly, we used the same data sources for mortality registrations across countries and mortality data was available from STMF and WHO sources similarly for all countries. It has previously been shown that European countries all have reliable and high-quality all-cause mortality registrations (please see reference 33 of the revised manuscript: *Mathers CD, Fat DM, Inoue M, Rao C, Lopez AD. Counting the dead and what they died from: an assessment of the global status of cause of death data. Bull World Health Organ. 2005;83(3):171-7)*. There are indeed differences in assignment of COVID-19 as cause of death between countries, which we have discussed as a limitation of the study in the revised Discussion page 18, lines 435-462. However, with regards to all-cause mortality registrations and accordingly our calculations of total person-years of life lost, we have no evidence of variation in the quality of all-cause mortality registrations between the studied countries.

Accordingly, as all input data, including the transition probabilities and prevalence estimates for each country were derived from data collected in that country using the same protocols across all the studied countries, the variability in the transition probabilities derived from different countries are likely to represent between-country differences. When comparing incidence of disease and mortality rates between the countries, we did not find any unexpected patterns of disease incidence or mortality, or any differences in country estimates of incidence or prevalence of disease that were incompatible with previous studies.

Regardless of the similarity of the data collection methods, the uncertainty intervals vary between countries due to differences in sample size of the studies and fluctuations in disease and mortality rates over time. Please see our response to point 5 below for further details regarding uncertainty intervals and how that is different between countries.

We have highlighted the point regarding between country comparisons in the discussion page 20, lines 504-516:

"*To enable between-country comparisons, we included countries where the required input data for the Markov modelling strategy were available and collected following standardized and harmonized protocols. ELSA and SHARE studies have recruited broadly representative samples of the population aged 50 and over, and their cohabiting partners, and the data collection protocols were the same between countries. We only included countries if ELSA or SHARE data were collected at two or more time-points, resulting in the list of the 18 countries. Although there are between country differences in identification and registration of COVID-19 as cause of death between countries, it has previously been shown that the countries included in this study have reliable all-cause mortality registrations [32], ensuring the comparability of our estimates of total person-years of life lost between countries. Although data sources followed similar data collection strategies, the uncertainty intervals vary between countries due to differences in sample size of the studies and fluctuations in disease and mortality rates over time (details provided in S1 Appendix page 14).*"

5- The authors mention Monte Carlo simulations to generate uncertainty intervals, but providing a more detailed explanation of how variation in transition

probabilities across different countries was managed would enhance the transparency of the model.

Our Response: Thank you for your feedback. We have now added details of the Monte Carlo simulation in the Table of mathematical calculations in S1 Appendix pages 3-8, and explained the Monte Carlo simulation in the Methods Page 13, lines 279-286 and S1 Appendix methods section SM6 on page 13.

Methods page 13, lines 279-286: "*Probabilistic sensitivity analysis*

To explore the impact of parameter uncertainty on model outputs, we conducted a probabilistic sensitivity analysis using Monte Carlo simulation. The procedure entails sampling from specified distributions for the input parameters that were used in the model for each data cycle. We calculated 500 iterations to estimate 95% uncertainty intervals for output variables. Uncertainty in model parameters increases over time and varies between countries. Further details of the probabilistic sensitivity analysis can be found in S1 Appendix pp 13-14 and the statistical formula are provided in S1 Appendix pp 3-8."

S1 Appendix Methods: *"**SM6: Probabilistic Sensitivity Analysis (Monte Carlo simulation)***

To derive uncertainty intervals for all outputs we ran Monte Carlo simulations with 500 iterations. Each input parameter of the Markov models, including baseline prevalence estimates, transition probabilities, and the calendar effects for transition probabilities, is entered as a normal distribution with mean and standard errors, which are derived from the methods described above. In each iteration of the Monte Carlo simulation, each input parameter is randomly selected from these normal distributions. The 2.5 and 97.5 percentile of the distribution of outputs obtained from 500 iterations constitute the 95% uncertainty intervals. For more details, please see mathematical calculations presented in the Table above under the "Statistical Methods" section.

The width of the 95% uncertainty intervals depend on the standard error of the input values. The standard errors of the transition probabilities increase with calendar years since baseline, due to multiplication of transition probabilities by calendar effects in each year (mathematical calculations presented above). This roughly translates to standard errors for transition probabilities increasing by a factor of the square root of years since baseline. All input data and the standard errors for the input parameters were derived from country specific data. The

standard errors for input estimates were larger in countries where data is derived from smaller sample sizes. Also, in countries or age-groups where there are larger year-on-year fluctuations in the incidence of disease, disability, or mortality rates (i.e., there is larger deviation in observations from the line of best fit) the standard errors from the transition probabilities obtained from the Cox-regression models were larger, leading to wider uncertainty intervals."

6- The use of Monte Carlo simulations to estimate uncertainty intervals is appropriate given the model's complexity and reliance on probabilistic transitions. However, it could be helpful to explain why 500 iterations were chosen and whether higher or lower numbers of iterations were tested to assess their impact on the stability of the uncertainty estimates.

Our Response: Thank you for your query. There are no robust guidelines, that we are aware of, to determine the number of iterations in Monte Carlo simulations, and the choice appears to be rather arbitrary. In these analyses, there was very little change in uncertainty intervals in the models run with ~200-300 iterations and models run with 1000 iterations. We ran models with 500 iterations, to strike a balance between accuracy and computational feasibility.

7- The estimation of PYLL (person-years of life lost) with and without disability is a strength of the analysis. However, given the lack of direct data on disability-related mortality in many countries, the assumptions used to estimate these inputs should be better justified. It would be useful to explain why hazard ratios from the UK were applied across other countries.

Our Response: Thank you for your feedback and for bringing the lack of clarity in this point to our attention. The hazard ratios for each country were derived from the SHARE data collected in that country. The hazard ratios for the UK (derived from ELSA) were **not** applied to other countries.

We have elaborated on the methods, assumption, and the validity and reliability of the methods to derive hazard ratios and mortality rates by disability status in the revised manuscript. The methods and reliability of the methods to derive expected and observed

mortality rates by disease and disability status are now explained in the Methods page 11, line 236, in the section subtitled "*Analyses by disability status",* with further details presented in S1 Appendix pages 11 and 12 in sections subtitled "*Mortality rates by disease and disability status"* and "*Assessment of the reliability of the method for estimation of mortality rates by disability status"*.

For brevity, we only quote excerpts of these sections here:

"We assumed the relative risk, or the hazard of mortality with disability to the hazard of all-cause mortality remains proportional over time. This assumption translates to increases in mortality rates with disability being a multiplication of the hazard ratio larger than increases observed in all-cause mortality. The method therefore is likely to account for older persons and those with disability being disproportionately affected over the pandemic".

To test this assumption and for illustration purposes (S1 Appendix Figure C), we present the estimated and expected mortality rates with and without disability for men and women aged 60 and 80 in Poland, the United Kingdom, and Switzerland where excess mortality during 2020-2022 was higher, average levels, or lower among the 18 studied countries. Our estimates of the increase in mortality rates from 2017 to the 2020-2022 pandemic years were substantially higher for mortality with disability than mortality without disability, and in Poland compared to the United Kingdom and Switzerland. The increase in mortality rates with disability were larger for persons aged 80 than the younger age-group, and larger for men than for women."

S1 Appendix Figure C: Comparison of mortality rates expected in the absence of the pandemic with estimated actual mortality rates with and without disability over 2020-2022.

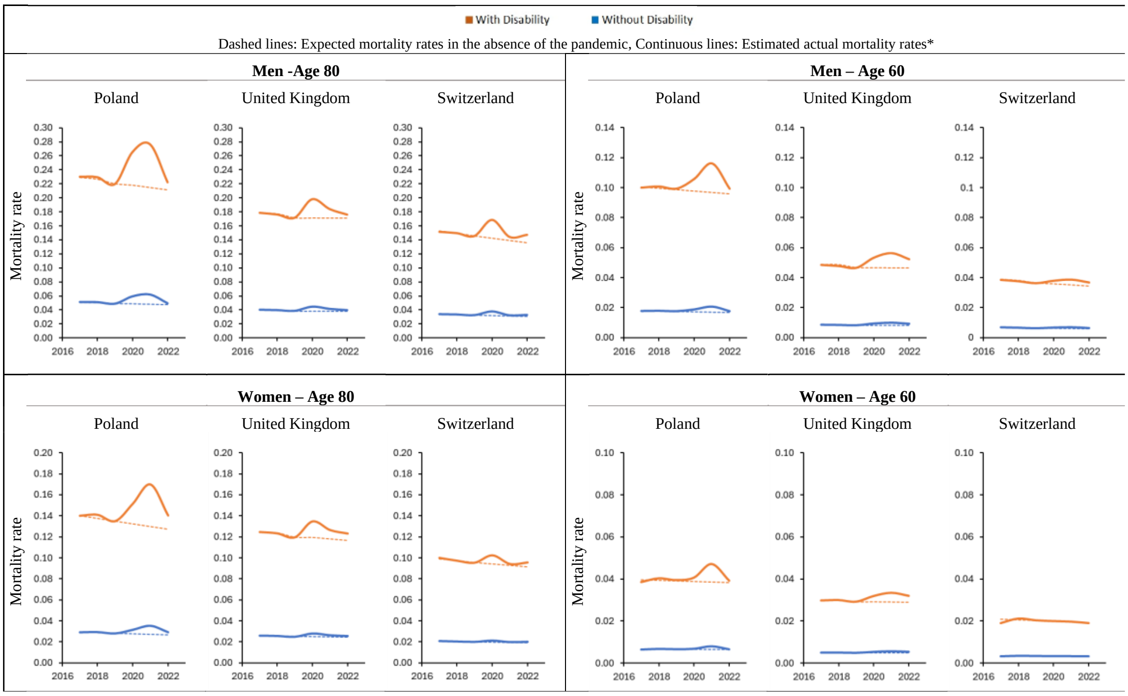

Of note, we found that using our method for estimating mortality rates by disability, on average across all countries, over half of the "excess" deaths in 2020-2022 were in persons with disability (please see S1 Appendix Table F). The estimates of excess mortality by age and disability shows the proportional hazards assumption has captured the disproportionate effect of excess mortality amongst older people and those with disability.

"We defined excess mortality as the difference between observed all-cause mortality rates than those expected if the pandemic had not happened, and pre-pandemic trends had continued. We examined the proportion of excess deaths that would be associated with disability in our calculations. In all 18 countries combined, we estimated 65% of excess deaths in the 80+ age group to be with disability. Disability accounted for 44 % of excess mortality in the 65-79 age group, 24% of excess mortality in the 50-64 age group, and 11% in 35-49 age group. The proportion of excess deaths by disability status by age-group and country are presented in S1 Appendix Table F".

S1 Appendix Table F: Proportion of excess deaths in 2020-2022 that was estimated to occur with disability by age-group.

	Proportion of excess mortality associated with disability by age-group			
	**35-49**	**50-64**	**65-79**	**80-100**
**All 18 countries**	11 %	24 %	44 %	65 %
**Switzerland**	5 %	13 %	31 %	54 %
**Sweden**	7 %	17 %	38 %	56 %
**Denmark**	9 %	21 %	42 %	61 %
**France**	9 %	21 %	43 %	66 %
**The Netherlands**	7 %	18 %	38 %	54 %
**Italy**	5 %	15 %	41 %	71 %
**Portugal**	14 %	27 %	45 %	55 %
**Germany**	13 %	27 %	47 %	68 %
**Belgium**	16 %	32 %	52 %	69 %
**United Kingdom**	18 %	35 %	50 %	60 %
**Austria**	6 %	19 %	43 %	68 %
**Greece**	2 %	16 %	39 %	55 %
**Slovenia**	7 %	17 %	41 %	67 %
**Czech Republic**	13 %	26 %	49 %	67 %
**Hungary**	19 %	26 %	38 %	63 %
**Spain**	5 %	16 %	45 %	75 %
**Poland**	18 %	30 %	42 %	67 %
**Estonia**	15 %	29 %	50 %	69 %

Data on mortality rates by disability status are scarce. We only found data on mortality rates by disease and disability using a different definition than the one applied in this study from the UK Office for National Statistics (ONS). Comparisons indicated our estimates of mortality by disability based on the proportional hazard assumption are unlikely to have been underestimated (please see S1 Appendix Figure D).

"To evaluate the reliability of this assumption with independent data, we compared model outputs with observed all-cause and COVID-19 mortality rates by disability status over the pandemic years in the UK where such data were available. A report from the Office for National Statistics provides age-standardized all-cause and COVID-19 mortality rates by disability status between January 2020 and March 2022 [17]. Disability status was defined using the self-reported responses to the 2011 Census question; "Are your day-to-day activities limited because of a health problem or disability which has lasted, or is expected to last, at least 12 months? - Include problems related to old age" (Yes, limited a lot; Yes, limited a little; and No). We compared these estimates with age-standardized mortality rates by disease and disability status obtained from our model for the UK. Given the differences in definitions and ascertainment, we did not expect the estimates for mortality to be an exact match. Comparison

of the estimates indicate our mortality estimates by disability status over the pandemic years are unlikely to be underestimated (S1 Appendix Figure D)".

S1 Appendix Figure D: Age-standardized all-cause and COVID-19 mortality rates in men and women in 2020 and 2021 in the United Kingdom by disease and disability status, comparing the model outputs with those reported by the Office for National Statistics

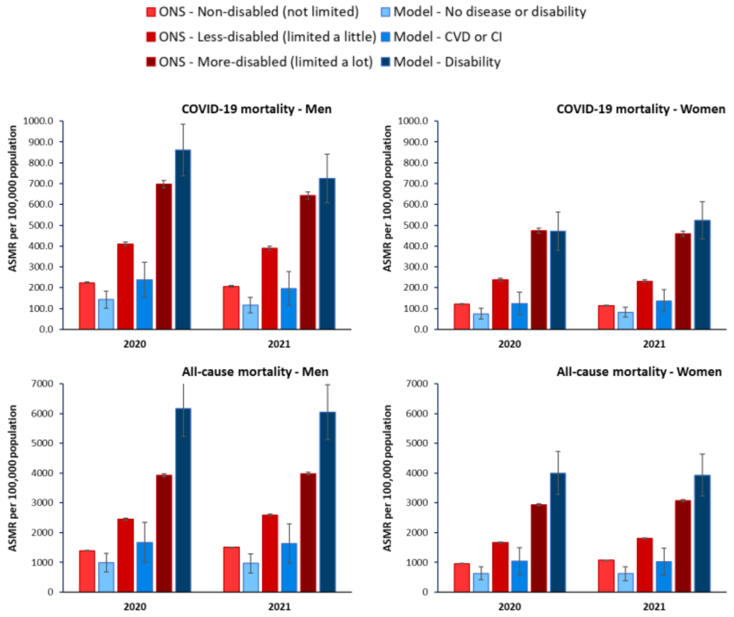

The validity and reliability of the methods to assess mortality by disability status are discussed in the discussion page 17, line 405 to 424.

"A limitation of this study is the lack of observed mortality data by disability status during the pandemic. We estimated mortality by disease and disability over the pandemic years based on hazard ratios obtained in the preceding years. A concern with this method is that the pandemic may have disproportionately affected the rates of death among those with disability, especially among the older age groups. Assuming the hazard of mortality with disability to the hazard of all-cause mortality remains proportional over time, the estimated increases in mortality rates with disability would be a multiplication of the hazard ratio larger than increases observed in all-cause mortality by age and sex. We consequently estimated considerably larger increases in mortality rates with disability than without disability, especially in the older age-groups and in countries where excess mortality was higher in 2020-2022. Among the 18 countries, using this method, we estimated that on average over half (55%) of excess deaths were in persons with disability. By age-group, we estimated 65% of excess deaths in the 80+ age-group, 44% in the 65-79, 24% in the 50-64, and 11% in the 35-49 age groups were in persons with disability.

The estimates of excess mortality by age and disability in S1 Appendix Table F shows the proportional hazards assumption has likely captured the disproportionate effect of excess mortality amongst older people and those with disease and disability. Comparison of our mortality estimates for 2020-2022 for the UK with those obtained from the UK Office for National Statistics [28] presented in S1 Appendix Figure D, indicates our estimates of mortality by disability based on the proportional hazard assumption are unlikely to have been underestimated.

We defined the person-years of life lost due to excess mortality during 2020-2022 as the years of life that would have been expected to be lived if the pandemic had not happened. A large proportion of PYLL with disability is therefore accounted for by people who were living without disability at the time of death, but were predicted to have developed disability in the future if the pandemic had not resulted in premature death. As total mortality rates are captured by observed data, given over half of the excess mortality occurred in persons with disability in our calculations, and a prevalence of ~10% for disability at population level, it is unlikely that errors in estimation of mortality rates among those with disability would be large enough to materially change the conclusions of this study with regards to proportions of PYLL with and without disability."

8- The two extreme scenarios—either assuming all reductions in non-COVID deaths were real or assuming they resulted from misclassification as COVID-19 deaths—offer a reasonable approach to managing uncertainty in cause of death. However, the analysis could benefit from more discussion of the likely midpoint between these extremes, as well as an exploration of intermediate assumptions and how they might impact the final results.

Our Response: Thank you for your suggestion. As criteria for assignment of COVID-19 as cause of death and testing rates changed over time within each country, and due to co-occurrence of other causes of deaths with COVID19, with the data that is available, unfortunately, it is not possible to provide a reasonably precise estimate of the likely midpoint estimate to disentangle the PYLL due to COVID19 deaths from those related to other causes. Providing the median of the range as an estimate of PYLL due to COVID/non-COVID related mortality for all countries may be misleading as it may not sufficiently present the

uncertainty in the estimates of PYLL by cause of death. We therefore presented the results of PYLL by registered COVID /non-COVID deaths as a range between two extreme scenarios to account for the uncertainty in these estimates.

9- The incorporation of time-specific transition probabilities based on pre-pandemic trends enhances the dynamic nature of the model. However, since the COVID-19 pandemic significantly disrupted healthcare access, it would be useful to discuss how these disruptions were accounted for in the model beyond mortality rates (e.g., access to preventive care or management of chronic diseases).

Our Response: Thank you for raising an interesting point with regards to the non-mortality impact of healthcare access. For this study, we only estimated the mortality impact of the pandemic in terms of person-years of life lost due to premature deaths that occurred during 2020-2022. For excess deaths that would not have been expected under pre-pandemic trends in 2020-2022, we calculated the life-years that would have been expected to be lived if the pandemic did not happen and pre-pandemic trends had continued. Disrupted provision of healthcare and preventative measures and related changes in disease and disability trends after the onset of the pandemic, do not affect years of life that would have been expected to be lived if the pandemic had not happened.

Persons may have experienced higher incidence of disease and disability due to healthcare disruptions. Estimating the non-mortality impact of the pandemic related healthcare disruptions (for example on future disease prevalence) for persons who survived the pandemic were beyond the scope of this study. Disruptions to healthcare access, prevention, and chronic disease management are likely to result in higher-than-expected mortality rates beyond 2022. However, with the data that is available, it is not possible to quantitatively predict the magnitude of excess deaths in the future. We were therefore only able to assess life-years lost for deaths that occurred in 2020-2022 where observed mortality rates are available. We found that although life-years lost due to COVID declined after 2021 parallel to introduction of vaccines, non-COVID related life-years lost continued to increase to 2022. Disrupted healthcare access and decrease in prevention efforts are likely to be part of the explanations for the person-years of life lost that were not explained by registered COVID-19 deaths, and the impact is likely to continue to the future.

Please see the discussion page 19, lines 463-473, where this point is discussed:

"*We observed a decline in person-years of life lost (PYLL) due to COVID-19 deaths after 2021, coinciding with the introduction of vaccines. However, PYLL due to other causes continued to increase from 2020 to 2022 in most of the 18 countries." ... "These findings along with the higher proportion of non-COVID-related PYLL in older age groups, underscore the potential long-term implications of the pandemic's indirect impacts, including reduced or delayed provision of care and services for non-COVID-related conditions, as well as the effects of lockdowns and epidemic containment measures on lifestyle behaviours. These effects may have disproportionately affected older people and countries with lower GDP per capita."*

Unfortunately, we are not able to disentangle the impact of disrupted healthcare provision from the impact of other factors during the pandemic, as granular data to evaluate the effects of each factor separately are not available. We agree that the impact of the disruptions in healthcare access at the height of the pandemic are likely to continue to the future, and we would likely observe higher than expected non-COVID deaths (resulting in life-years lost) for several years after 2022. Estimating the mortality impact after 2022 was beyond the scope of this study.

10- Conducting sensitivity analyses using Monte Carlo simulations adds robustness to the findings, but additional information on the distribution assumptions for the parameters would be helpful. For example, are transition probabilities assumed to follow a normal or log-normal distribution?

Our Response: Thank you for this query. We agree that sufficient detail with regards to the Monte Carlo simulation was not provided. We have now included a section in S1 Appendix Pages 3-8 to describe the mathematical formula for the calculations and show the transition probabilities were assumed to follow a normal distribution for each single year of age and sex strata, denoted with a mean and standard error. The details of the Monte Carlo simulations are presented in S1 Appendix page 13, under subsection "***SM6: Probabilistic Sensitivity Analysis (Monte Carlo simulation)***"

"To derive uncertainty intervals for all outputs we ran Monte Carlo simulations with 500 iterations. Each input parameter of the Markov models, including baseline prevalence

estimates, transition probabilities, and the calendar effects for transition probabilities, is entered as a normal distribution with mean and standard errors, which are derived from the methods described above."

Please see subsection SM6 on S1 Appendix page 13 for further details regarding the Monte-Carlo simulations, and S1 Appendix pages 3-8 for mathematical calculations.

11- The authors mention that the methods and assumptions used in this Markov model have been validated for the UK. It would strengthen the manuscript to elaborate on any cross-country validation performed to ensure the assumptions are valid across the 18 European countries, given the variations in healthcare systems, demographic structures, and responses to COVID-19. If such validation was not feasible, this limitation should be explicitly stated.

Our Response: Thank you for raising this point. Epidemiological data from independent studies to enable such validations for all countries were not available. We have added a paragraph to the discussion to elaborate on this point. Please see page 21, lines 517-526:

"We have previously tested the validity of the Markov method in prediction of the prevalence of cardiovascular disease, dementia, and mortality in the UK against data obtained from independent studies [19, 20, 28] and in this analysis, compared mortality by disability status with data from the ONS for the UK. We were not able to conduct similar validation exercises for disease prevalence for other countries due to lack of data availability. It should be noted however that the data sources used in modelling data for all countries were harmonious, and although rates vary between countries, the shapes of the distributions and trends over time of the parameters were similar between countries. We therefore assumed that the Markov method would reasonably be applicable for other countries using country specific data that were collected from harmonized and standardized studies"

Please see S1 Appendix Figure E for trends in cardiovascular disease and dementia over time in the 18 countries.

12- The manuscript could benefit from a more explicit discussion of how missing data (if any) were handled in the model. Given that longitudinal data sources like ELSA and SHARE may contain incomplete information, it is important to explain how this was addressed (e.g., through imputation, exclusion, or other methods).

Our Response: Thank you for your query. The Cox-proportional hazards models to obtain transition probabilities were adjusted for age and sex only so that the obtained transition probabilities would be mathematically equivalent to the weighted average of transition probabilities across levels of risk factors, comorbidities, and severity of the condition. With recruitment of refreshment samples and application of survey weights, the SHARE and ELSA study samples were broadly representative of the population aged 50+ at each wave. We therefore conducted analysis according to the number of waves that participants attended the study until being censored. In these studies, data were collected from the participants, proxy informants (for example for those with cognitive impairment or disability), or self/proxy-reported doctor diagnosis of diseases. Missing values for the variables required to conduct the analysis were negligible.

Please see S1 Appendix page 9, subsection ***SM2: Country-specific transition probabilities between states representing CVD, cognitive impairment, dementia and disability by age and sex***

"The baseline age-sex-specific probability of transition from state$_i$ to state$_j$ in the model (hereafter referred to as transition probability (P_{ij})) for the mid-point of the data collection timeframe of each SHARE (/ELSA) study were obtained by fitting a Cox proportional hazards regression model with state$_j$ as outcome and terms for age, sex, interaction of age and sex, country, interaction terms for country with age and sex, and a variable defining the initial state (state$_i$)." .... "Transitions from wave n to wave n+1 in ELSA or SHARE were pooled together so that each individual contributed as many observations as corresponded to the number of 2-, 4- or 6-year epochs in which they participated in the study until being censored".

13- Given that the study spans the period from 2020 to 2022, which included multiple waves of COVID-19 and the emergence of different variants (e.g., Alpha, Delta, Omicron), it would be helpful to discuss how, if at all, these evolving factors were

considered in the modeling. Was the pandemic treated as a uniform event, or did time-varying effects of different variants impact mortality and healthcare access?

Our Response: Thank you for raising this point. We agree modelling the effect of different COVID variants would be an interesting topic. To be able to disentangle life-years lost by COVID variants, we would need precise data for numbers of death by age and sex for each variant at population level by year and country. Unfortunately, such data is not available, and we were only able to investigate COVID-19 deaths as one entity.

14- The model assumes discrete health states with transitions between them. It would be useful to clarify if and how interactions between health states (e.g., cardiovascular disease co-occurring with dementia) were handled in the model. Can individuals occupy multiple health states simultaneously, and how might this affect transitions and mortality risk?

Our Response: Thank you for bringing the lack of clarity regarding this point to our attention. We have presented the health states in the Markov models and transitions between them in Figure 1. A participant can occupy one health state at a time. We have four different states for disability to account for co-occurrence of conditions: disability co-occurring with 1. cardiovascular disease (CVD), 2. with dementia, 3. with both CVD and dementia, or 4. disability without CVD, cognitive impairment or dementia.

Co-occurrence of cognitive impairment and disability is defined as dementia (compatible with DSM-IV criteria). Persons can transition from a state of CVD or cognitive impairment to a state of both CVD and cognitive impairment. We found that once persons develop disability the remaining life-expectancy is relatively short, and we do not have many transitions from disability with one condition to disability with another condition. Therefore, all such transitions are captured in the transitions to and between CVD and cognitive impairment to both these conditions (i.e., transition probabilities from CVD or cognitive impairment to both were calculated based on total numbers that make that transition with or without co-occurrence with disability).

In S1 Appendix page 3 where we present mathematical formula, we have listed the health states in the Markov models:

"S_1: Disease-free state

S_2: Cardiovascular disease, no disability

S_3: Cardiovascular disease and cognitive impairment, no disability

S_4: Cognitive impairment, no disability

S_5: Cardiovascular disease and disability

S_6: Cardiovascular disease with cognitive impairment and disability (CVD+dementia+disability)

S_7: Cognitive impairment with disability (dementia+disability)

S_8: Disability, no cardiovascular disease or cognitive impairment

S_9 : COVID deaths

S_{10}: Non-COVID deaths"

15- The manuscript mentions the indirect impacts of the pandemic due to disruptions in healthcare services. It would be beneficial to expand on whether other external shocks (e.g., changes in policy or healthcare infrastructure strain) were modelled, particularly in relation to non-COVID causes of death. For example, were the lagged effects of disruptions (e.g., delayed cancer screenings) on long-term mortality, which may not fully manifest within the 2020-2022 window, considered?

Our Response: Please see our response to point 9 above. We investigated the life-years lost due to excess deaths that occurred during 2020-2022 by comparing Markov models that used mortality rates following pre-pandemic trends that were expected in the absence of the pandemic and Markov models that included observed mortality rates and those adjusted for registered COVID19 deaths during 2020-2022. We agree that the impact of the disruptions in healthcare access at the height of the pandemic are likely to continue to the future, and we would likely observe higher than expected non-COVID deaths (resulting in life-years lost) for

several years after 2022. Estimating the mortality impact after 2022 was beyond the scope of this study.

16- The model stratifies by age groups (35+, 65+, etc.), but it might be helpful to discuss whether age-cohort effects (i.e., differences between generations) were considered. For instance, older cohorts may have different health risks and exposures compared to younger cohorts. Even though the model is time-based, it could be valuable to discuss how aging dynamics and cohort-specific vulnerabilities were factored in or could affect the results.

Our Response: Thank you for your feedback. We have indeed accounted for cohort effects in our analyses. The cohort effects are accounted for by implementation of calendar effects for transition probabilities such that all transition probabilities are calendar-year specific for each age and sex, by country. We found age-specific incidence of cardiovascular disease, dementia, and mortality rates declined over time, and incidence of disability without cardiovascular disease or dementia slightly increased over time all following log-linear patterns in all countries (please see S1 Appendix Figures B and E). Implementation of the calendar effects ensures that we account for, for example, a lower incidence of CVD or dementia for an 80 year old person in any particular year compared to a person who was 80 years old several years before.

We have clarified this point in our methods description. Please see S1 Appendix pages 12 and 13 under section "SM4: Calendar effects for incidence of cardiovascular disease, dementia, and disability"

"To account for cohort effects, and to obtain calendar year specific transition probabilities at each age by sex and country, the effect of calendar time was imposed on the transition probabilities for each year since baseline to obtain transition probabilities for that age at the next calendar year.

The calendar decline in age-specific cardiovascular incidence, mortality, and incidence of dementia observed in ELSA and SHARE data was parallel across the 18 countries modelled in the present study. The parallel calendar declines were similarly observed in data obtained from Institute of Health Metrics and Evaluation [18] (S1 Appendix Figure E). Cox proportional hazard models with age, sex, and calendar year with interaction terms with country were fitted

to obtain the calendar effect for the transition probabilities to each of the health states. Calendar effects were assumed to become smaller in magnitude over time, similar to the calendar effects observed for the decline in mortality rates".

17- The mention of a bespoke R package developed by one of the authors is a positive step toward reproducibility. However, it would be useful to clarify whether this code will be made publicly available, as doing so would enhance the transparency of the modeling process.

Our Response: The R package was developed for a different project that was funded until mid-2020. Unfortunately, we do not have the resources to produce the R package in a publishable format with sufficient guidelines for users. To ensure reproducibility, we have provided a detailed step by step calculations for the Markov method in S1 Appendix pages 3-8. The Analysis can therefore be reproduced using any statistical software, including using the publicly available "markovchain" package in R.

The bespoke R package used in this study would produce the same results as would any other package following the same analytic strategy presented in S1 Appendix pages 3-8. The R package was developed for a prior collaborative project for feasibility of analysis and to ensure the different teams involved at different institutions worked with the same base model.

18- The Supplementary material contains valuable information such as Tables and Figures that aid in understanding the modeling assumptions and results. It is important to ensure that the main manuscript clearly directs readers to these Tables and Figures, especially for those who may not read the Supplementary material in full detail.

Our Response: Thank you for your suggestion. We have checked to ensure we have referred to the Supplementary material (now renamed S1 Appendix in line with Journal requirements) appropriately in the methods and results section.

Any attachments provided with reviews can be seen via the following link:

-- --- General editorial requests:

(Note: not all will apply to your paper, but please check each item carefully)

Thank you for raising the points below. We have implemented all elements that were applicable to this study.

* We ask every co-author listed on the manuscript to fill in a contributing author statement, making sure to declare all competing interests. If any of the co-authors have not filled in the statement, we will remind them to do so when the paper is revised. If all statements are not completed in a timely fashion this could hold up the re-review process. If new competing interests are declared later in the revision process, this may also hold up the submission. Should there be a problem getting one of your co-authors to fill in a statement we will be in contact. Please do not add or remove authors without first discussing this with the handling editor. You can see our competing interests policy here: http://journals.plos.org/plosmedicine/s/competing-interests.

* Please upload any Figures associated with your paper as individual TIF or EPS files with 300dpi resolution at resubmission; please read our Figure guidelines for more information on our requirements: http://journals.plos.org/plosmedicine/s/Figures. While revising your submission, please upload your Figure files to the PACE digital diagnostic tool, https://pacev2.apexcovantage.com/. PACE helps ensure that Figures meet PLOS requirements. To use PACE, you must first register as a user. Then, login and navigate to the UPLOAD tab, where you will find detailed instructions on how to use the tool. If you encounter any issues or have any questions when using PACE, please email us at PLOSMedicine@plos.org.

* Please ensure that the paper adheres to the PLOS Data Availability Policy (see http://journals.plos.org/plosmedicine/s/data-availability). For data residing with a third party, authors are required to provide instructions with contact information (web or email address) for obtaining the data. Please also note that a study author cannot be the contact person for any data. Please direct readers to a non-author institutional point of contact, such as a data access or ethics committee. Providing a durable point of contact ensures data will be accessible even if an author changes email addresses, institutions, or becomes unavailable to answer requests.

* We expect all researchers with submissions to PLOS in which author-generated code underpins the findings in the manuscript to make all author-generated code available without restrictions upon publication of the work. In cases where code is central to the manuscript, we may require the code to be made available as a condition of publication. Authors are responsible for ensuring that the code is reusable and well documented. Please make any custom code available, either as part of your data deposition or as a Supplementary file. Please add a sentence to your data availability statement regarding any code used in the study, e.g. "The code used in the analysis is available from Github [URL] and archived in Zenodo [DOI link]" Please review our guidelines at https://journals.plos.org/plosmedicine/s/materials-software-and-code-sharing and ensure that your code is shared in a way that follows best practice and facilitates reproducibility and reuse. Because Github depositions can

be readily changed or deleted, we encourage you to make a permanent DOI'd copy (e.g. in Zenodo) and provide the URL.

FORMATTING - GENERAL

* Abstract: Please structure your abstract using the PLOS Medicine headings (Background, Methods and Findings, Conclusions). Please combine the Methods and Findings sections into one section.

* Thank you got providing an Author Summary. Please note that this is expected to be short, non-technical and the aim is to make your research accessible to a wide audience that includes both scientists and non-scientists. The Author Summary should immediately follow the Abstract in your revised manuscript. Ideally each sub-heading should contain 2-3 single sentence, concise bullet points containing the most salient points from your study. We ask that you revise the section entitled 'What Did the Researchers Do and Find?' to be shorter and use less technical language. Also, in the final bullet point of 'What Do These Findings Mean?', please include the main limitations of the study in non-technical language. Please see our author guidelines for more information: https://journals.plos.org/plosmedicine/s/revising-your-manuscript#loc-author-summary.

FIGURES AND TABLES

* Please provide titles and legends for all Figures and Tables (including those in Supporting Information files).

* Please define all abbreviations used in each Figure/Table (including those in Supporting Information files).

* Please consider avoiding the use of red and green in order to make your Figure more accessible to those with color blindness.

SUPPLEMENTARY MATERIAL

* Please cite your Supporting Information as outlined here: https://journals.plos.org/plosmedicine/s/supporting-information

REFERENCES

* PLOS uses the numbered citation (citation-sequence) method and first six authors, et al.

* Please ensure that journal name abbreviations match those found in the National Center for Biotechnology Information (NCBI) databases (http://www.ncbi.nlm.nih.gov/nlmcatalog/journals), and are appropriately formatted and capitalised.

* Where website addresses are cited, please include the complete URL and specify the date of access (e.g. [accessed: 12/06/2023]).

* Please also see https://journals.plos.org/plosmedicine/s/submission-guidelines#loc-references for further details on reference formatting.

OBSERVATIONAL STUDIES

* Please ensure that the study is reported according to the appropriate reporting guideline (available from: https://www.equator-network.org/reporting-guidelines) and include the completed checklist as Supporting Information. Please add the following statement, or similar, to the Methods: "This study is reported as per the [insert reporting guideline name and abbreviation here] (S1 Checklist)." When completing the checklist, please use section and paragraph numbers, rather than page numbers.

* For all observational studies, in the manuscript text, please indicate: (1) the specific hypotheses you intended to test, (2) the analytical methods by which you planned to test them, (3) the analyses you actually performed, and (4) when reported analyses differ from those that were planned, transparent explanations for differences that affect the reliability of the study's results. If a reported analysis was performed based on an interesting but unanticipated pattern in the data, please be clear that the analysis was data driven.

* Please state in the Methods section whether the study had a prospective protocol or analysis plan. If a prospective analysis plan (from your funding proposal, IRB or other ethics committee submission, study protocol, or other planning document written before analyzing the data) was used in designing the study, please include the relevant document(s) with your revised manuscript as a Supporting Information file to be published alongside your study and cite it in the Methods section. A legend for this file should be included at the end of your manuscript. If no such document exists, please make sure that the Methods section transparently describes when analyses were planned, and when/why any data-driven changes to analyses took place. Changes in the analysis, including those made in response to peer review comments, should be identified as such in the Methods section of the paper, with rationale.
