# Supplementary material for: Direct and indirect impacts of the COVID-19 pandemic on life expectancy and person-years of life lost with and without disability: A systematic analysis for 18 European countries, 2020–2022
Source: PLoS Med. 2025 Mar 11;22(3):e1004541. doi: 10.1371/journal.pmed.1004541 (PMC11896051; doi:10.1371/journal.pmed.1004541)
Supplement: Attachment — Submitted filename: 0 Response to Reviewers and Editors R2.pdf [file pmed.1004541.s003.pdf]

Response to Editors' and Reviewers' comments:

Requests from Editors:

> Thank you for submitting your revised manuscript. As you can see, the reviewers were positive about your amendments and we are pleased to be moving forward with your submission. One of the reviewers (Reviewer 3) provided some additional suggestions. In your next revision, we ask that you kindly provide a point-by-point response to these comments as well as the editorial requests outlined below.

We are pleased to hear you would like to move forward with our submission. We have revised the manuscript to implement the requests from the Editors and address the comments from the reviewers. Below we provide a point-by-point response. We hope the revised version meets the requirements for publication.

(1) Data Availability Statement

> Thank you for providing a Data Availability Statement. However, we kindly ask that you provide some extra information for the benefit of our readers. For data residing with any third party, authors are required to provide instructions with contact information (web or email address) for obtaining the data. Please provide this information with respect to the ELSA and SHARE studies. Please also note that a study author cannot be the contact person for any data. Please direct readers to a non-author institutional point of contact, such as a data access or ethics committee. Providing a durable point of contact ensures data will be accessible even if an author changes email addresses, institutions, or becomes unavailable to answer requests.

Thank you for your feedback. We have amended the data availability statement accordingly.

(2) Author Summary:

> We ask that you kindly amend your Author Summary a little more. Please use bullet points. (Ideally each sub-heading should contain 2-3 single sentence, concise bullet points containing the most salient points from your study.) Please provide an expansion of GDP at first use in the Author Summary. The section entitled 'What Did the Researchers Do and Find?' would benefit from being shortened further. We also ask if the authors could revise the first 2 sentences of the last section to include a more concrete example of a study limitation. It may be best to

remove the first 2 sentences entirely, start this section with the sentence ' The increasing PYLL…' and write about a study limitation towards the end of this section instead. The authors could say something along the lines of 'Limitations of this study include the lack of mortality data by disability status that was available during the pandemic and the subsequent reliance on estimates.'

Thank you for your suggestions. We have revised the Author Summary accordingly and we hope the revised version is suitable.

Requested Editorial corrections:

1. Please ensure that the funders and grant numbers match between the Financial Disclosure field and the Funding Information tab in your submission form. Note that the funders must be provided in the same order in both places as well. Currently, the grant from HORIZON EUROPE European Institute of Innovation and Technology is not mentioned in the Financial Disclosure.

Thank you for your note. The funding from European Institute of Innovation and Technology is included in the financial disclosures.

> *"SAA was supported by institutional funding from Imperial College London and the European Institute of Innovation and Technology, EIT-Health (210920)".*

2. Please upload a file showing your changes either highlighted or using a track changes function in your word processor. This document should be uploaded as the item type 'Revised Article with Changes Highlighted.' This will enable a swift editorial assessment of your manuscript. If you are submitting a PDF please ensure that the changes are clearly highlighted

Thank you. The revised article with tracked changes is uploaded with the submission.

Comments from Reviewers:

Reviewer #1:

The authors have responded very well not only to my comments but also to those of the other reviewers. In addition, they have included many of them in the new version of the manuscript. I have no further comments.

Thank you for your feedback. We are pleased to hear we have been able to address your concerns and feedback.

Reviewer #3:

Thank you for thoroughly addressing the comments and suggestions I provided in my initial review. The revisions have significantly improved the clarity, depth, and methodological rigor of the manuscript. I appreciate the detailed responses to each point, particularly the explanations regarding the modelling choices, data handling, and statistical analyses. Below are some additional comments that, if addressed, could further strengthen the manuscript.

We thank you for reviewing the revised version of the manuscript. We are pleased to hear we have been able to address your concerns and suggestions.

- While the Markov model uses age- and sex-specific strata, the authors could discuss how granularity in the inputs (e.g., socioeconomic or regional differences within countries) might impact the outputs.

Thank you for your suggestion. The model inputs were derived separately for each country using data from SHARE and ELSA studies. With application of survey weights, the participants in the SHARE and ELSA studies were broadly representative samples of the population. Therefore, the derived inputs represent a weighted average across the regional and socioeconomic strata of the population. Given the representative nature of the data from ELSA and SHARE studies (with application of survey weights), the calculations are mathematically equivalent to those if the analyses were stratified by socioeconomic status, region, or other risk factor strata. Therefore, model outputs would not be affected. We have revised the S1 Appendix and added the below paragraph to the S1

Appendix Methods section on page 1 to clarify this point, which is also discussed in the S1 Appendix Table A.

> *"SHARE study participants were randomly drawn from those aged 50+ in population registers and their cohabiting partners [1-7], ….. For each study in SHARE and ELSA, refreshment samples are recruited at each biannual wave after baseline to maintain representativeness of the study population. With application of survey weights, therefore, estimates of disease incidence and prevalence from these studies represent a weighted average across strata of disease severity, associated comorbidities, and socioeconomic status of the population (Table A)."*

> - While the authors chose 500 iterations for Monte Carlo simulations, it may be useful to present results of convergence diagnostics or explore whether higher iterations (e.g., 1000) would meaningfully change uncertainty intervals.

Thank you for your query. In testing the models to find the optimal number of iterations, we found that higher numbers of iterations resulted in narrower uncertainty intervals, and therefore, the presented uncertainty intervals for models with 500 iterations would not underestimate the width of the uncertainty intervals compared to models run with 1000 iterations. To avoid overestimation of the level of precision of the presented results, all model outputs are presented in thousands. We found the point estimate and uncertainty intervals would only change in the decimal places when the number of iterations exceeded 200-300 for each country. Higher numbers of iterations did not change the presented results in thousands. We therefore ran and presented results from models with 500 iterations, to strike a balance between accuracy and computational feasibility.

> -Provide additional justification for the use of normal distributions for transition probabilities. For some events (e.g., rare transitions), log-normal or beta distributions might be more suitable. Explain why normal distributions were deemed appropriate in this context.

Thank you for bringing the lack of clarity regarding this point to our attention. We do not make assumptions about the distribution of the transition probabilities in our calculations. The point estimates for age- and sex-specific transition probabilities by country were derived from Cox proportional hazards regression models, which are free from assumptions regarding the underlying distribution of the outcome. Detailed descriptions are provided in the S1 Appendix page 9 in the

section subtitled: "***SM2: Country-specific transition probabilities between states representing CVD, cognitive impairment, dementia and disability by age and sex***". To account for uncertainty in derived estimates for transition probabilities and other model inputs, we assume *the error* around each transition probability by single year of age and calendar year *is normally distributed* with the standard errors calculated as denoted in the Table of mathematical calculations in S1 Appendix pages 3-8. We have revised S1 Appendix page 13 to clarify this point:

> "***SM6: Probabilistic Sensitivity Analysis (Monte Carlo simulation)***
>
> *To derive uncertainty intervals for all outputs we ran Monte Carlo simulations with 500 iterations. To account for the uncertainty in model inputs, each input parameter of the Markov models, including baseline prevalence estimates, transition probabilities, and the calendar effects for transition probabilities by country, sex, single year of age, and where applicable, calendar year, is entered as a normal distribution with the standard errors derived from the methods described above. In each iteration of the Monte Carlo simulation, each input parameter is randomly selected from these normal distributions. The 2.5 and 97.5 percentile of the distribution of outputs obtained from 500 iterations constitute the 95% uncertainty intervals".* (S1 Appendix page 13)

Notations are provided in the Table of mathematical calculations in S1 Appendix pages 3-8, where the calculations for the point estimates and standard errors for model inputs by sex, single year of age and calendar year are provided:

$P_{i,j,a,0} \sim N(\omega_{i,j,a,0}, (\delta_{i,j,a,0})^2)$ where $P_{i,j,a,0}$ is the transition probability from state i to state j for age a at time 0. Transition probabilities are calculated by country and sex.

$C_{i,j} \sim N(\theta_{i,j}, (\nu_{i,j})^2)$ where $C_{i,j}$ is the calendar effect for the probability of transition from state i to state j

Since $P_{i,j,a,t} \sim N(\omega_{i,j,a,t}, (\delta_{i,j,a,t})^2)$ and $C_{i,j} \sim N(\theta_{i,j}, (\nu_{i,j})^2)$ -> The standard error for $P_{i,j,a,t}$ is approximately

$\sqrt{(\delta_{i,j,a-1,t-1})^2*(\nu_{i,j})^2+(\delta_{i,j,a-1,t-1})^2*(C_{i,j})^2+(P_{i,j,a-1,t-1})^2*(\nu_{i,j})^2}$, where $P_{i,j,a,t}$ is the transition probability from state i to state j for age a at time t.

- Given that pre-pandemic data trends were assumed to be linear or log-linear, a brief explanation of how non-linear patterns (if present) were tested and ruled out would strengthen the methodology.

Thank you for your suggestion. In our analyses of ELSA and SHARE data, consistent with other independent population-based studies and reports, time trends in mortality rates, and those for incidence of cardiovascular disease, dementia, and disability by age and sex were observed to be linear or log-linear over the two decades prior to the pandemic. Please see S1 Appendix Figure B (pp 20-25) for trends in mortality rates and S1 Appendix Figure E (page 33) for trends in incidence of cardiovascular disease and dementia by country. The modelling is based on the assumption that the most likely scenario in the absence of the pandemic would have been the continuation of pre-pandemic trends. We did not find evidence of non-linear trends in incidence of disease or mortality rates from SHARE or ELSA data or from external data sources and reports. In the absence of quantitative evidence for non-linear trends, there are countless hypothetical scenarios to model from which, given the highly hypothetical nature of such modelling, are unlikely to be informative. Indeed, it is not certain that pre-pandemic trends would have continued in the absence of the pandemic, and we have accounted for the uncertainty in the calendar trends in incidence of disease and mortality in Monte-Carlo simulations and have presented the resulting uncertainty intervals. Of note, the standard errors of the transition probabilities increase with time, reflecting the uncertainty in future projections increases over time. Details are provided in the S1 Appendix Table A on Page 16 under the "**Calendar trends**" subtitle.
